# Engineering Lewis Acidity in Zeolite Catalysts by Electrochemical Release of Heteroatoms during Synthesis

**DOI:** 10.1021/acs.chemmater.3c00552

**Published:** 2023-06-01

**Authors:** Gleb Ivanushkin, Michiel Dusselier

**Affiliations:** Center for Sustainable Catalysis and Engineering CSCE, Faculty of Bioscience Engineering, KU Leuven, B-3000 Leuven, Belgium

## Abstract

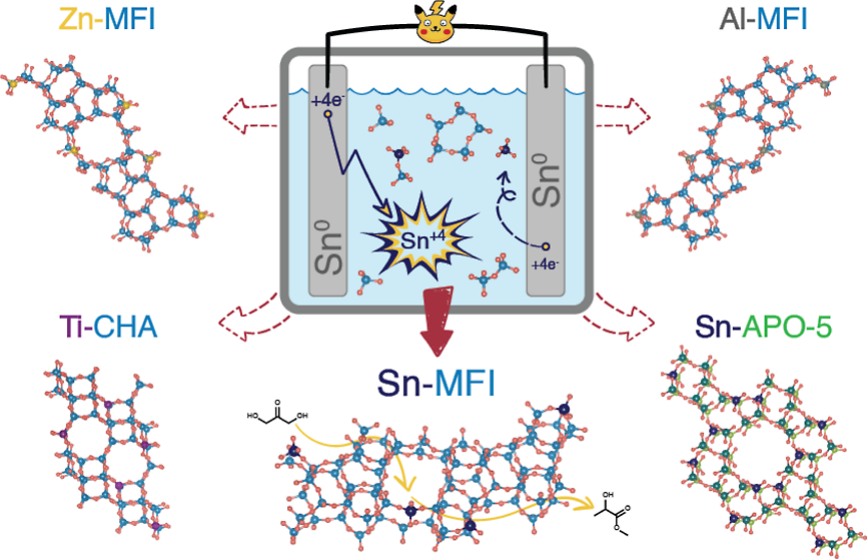

The creation of heteroatom nodes in zeolite frameworks is a challenging but rewarding pathway to superior materials for numerous catalytic applications. Here, we present a novel method for precise control over heteroatom incorporation by in situ anodic release of a desired metal during hydrothermal zeolite synthesis. The generic character of the technique and the applicability of the new synthesis reactor are shown across 3 zeolite structures crystallized and 4 electrode metals in two pH zones and by offering access to a new mixed-metal zeolite. The timed and voltage-controlled metal release offers a minimized interference between the metal precursor state and critical events in the zeolite’s crystallization. A mechanistic study for Sn-MFI revealed the key importance of controlled release: while keeping its concentration lower than in batch, a lot more Sn can be incorporated into the framework. The method grants access to 10× increased framework Lewis acid site densities (vs batch controls) for the most relevant stannosilicates. As a proof, the electro-made materials demonstrate higher productivity than their classic counterparts in lactate catalysis. This innovative approach effectively expands the synthesis space of zeolites.

## 1 Introduction

Zeolites are microporous materials that consist of connected silica and alumina tetrahedra. As performance materials in catalysis and separation, they hold levers over millions of tons of products, next to guiding the transition to a circular carbon economy. Introducing tetrahedral heteroatoms other than Al, such as B, Sn, Fe, Zn, and so forth, enhances or modifies their (Lewis) acidity, creating a confined single-atom catalyst.^1^ These so-called zeotypes (e.g., Sn-BEA or Ti-MFI) and especially stannosilicates have revolutionized biomass conversion and peroxide oxidations.^2,3^

Heteroatom-containing zeolites (meaning exclusively isomorphous substitution) can be synthesized by hydrothermal synthesis or by post-synthetic modification, each with their own advantages. In a classic bottom-up synthesis, all ingredients are mixed for subsequent crystallization in a closed batch reactor. Several powerful strategies have been reported (e.g., fluoride mineralization^4−6^ or dry gel conversion^7−9^), offering higher incorporation efficiencies. On the other hand, top-down approaches focus on modifying a parent zeolite structure by consecutive vacancy creation and defects healing with metal precursors.^10−16^

Zeolite formation depends on both kinetics and thermodynamics, and the prevalence of one over the other can lead to different active site densities and distributions. Unfortunately, obtaining a targeted active site (heteroatom) density is never straightforward and the heteroatom metal content is often too low for zeotypes, yielding suboptimal catalytic performances (mol_product_ kg_cat_^–1^ h^–1^). This results from a trade-off: as the heteroatom (metal) precursor in a bottom-up crystallization batch heavily interferes with the critical synthesis steps, such as Si solubility, nucleation, and crystal growth, its concentration has to be kept low.^17^ This is prominent in Sn-zeolite syntheses, where a high density of active sites is hard to achieve hydrothermally, and adding more Sn in the batch at the start hinders nucleation. Top-down methods suffer from the level of heteroatom being controlled by (or never decoupled from) the biased vacancies’ creation in the host.^18^ While nonconventional approaches in aluminosilicate zeolite synthesis often lead to exciting synthesis outcomes and new broadly applicable strategies (e.g., the radical-accelerated crystallization^19,20^ or ADOR^21^), novel methods targeting heteroatoms are scarce.

Here, we attain unprecedented levels of control over heteroatom incorporation in siliceous zeolites based on introducing conducting metal electrodes in bulk hydrothermal synthesis mixtures. The electrochemical heteroatom release by anodic oxidation can be precisely timed for minimal interference with zeolite nucleation and allows for unique concentration control during the growth phase. Depending on a new reactor design, this novel method provides zeolite synthesis control by additional degrees of freedom expressed in voltage, frequency, and electrode surface.

Furthermore, the voltage-based feeding of the system with very dilute but active heteroatom precursors allows one to synthesize zeolite catalysts with unprecedented Lewis acid site (LAS) densities. For certain stannosilicates, we show more than 15-fold increase in the framework Sn content versus syntheses in batch, and in catalytic tests, this leads to materials so rich in active sites that they demonstrate high productivities.

Sustainable processes and circular production, for example, fed by waste, CO_2_, and biomass will rely on a wide zeotype diversity (existing structures but with controlled Sn, Zn, or Ti inside) to facilitate new catalytic and adsorbent discoveries, as demonstrated by the track record of Ti-MFI and Zn- and Sn-BEA.^22−24^ Therefore, we illustrate the generic applicability of electrochemical-assisted synthesis by creating stanno-, zinco-, alumino-, and titanosilicates in both mildly acidic and highly basic media across three different zeolite frameworks. On top, we demonstrate a novel mixed-metal zeotype (Zn,Sn-MFI).

## 2 Proof of Concept and Modes of Control: Sn-MFI

**Figure 1:**
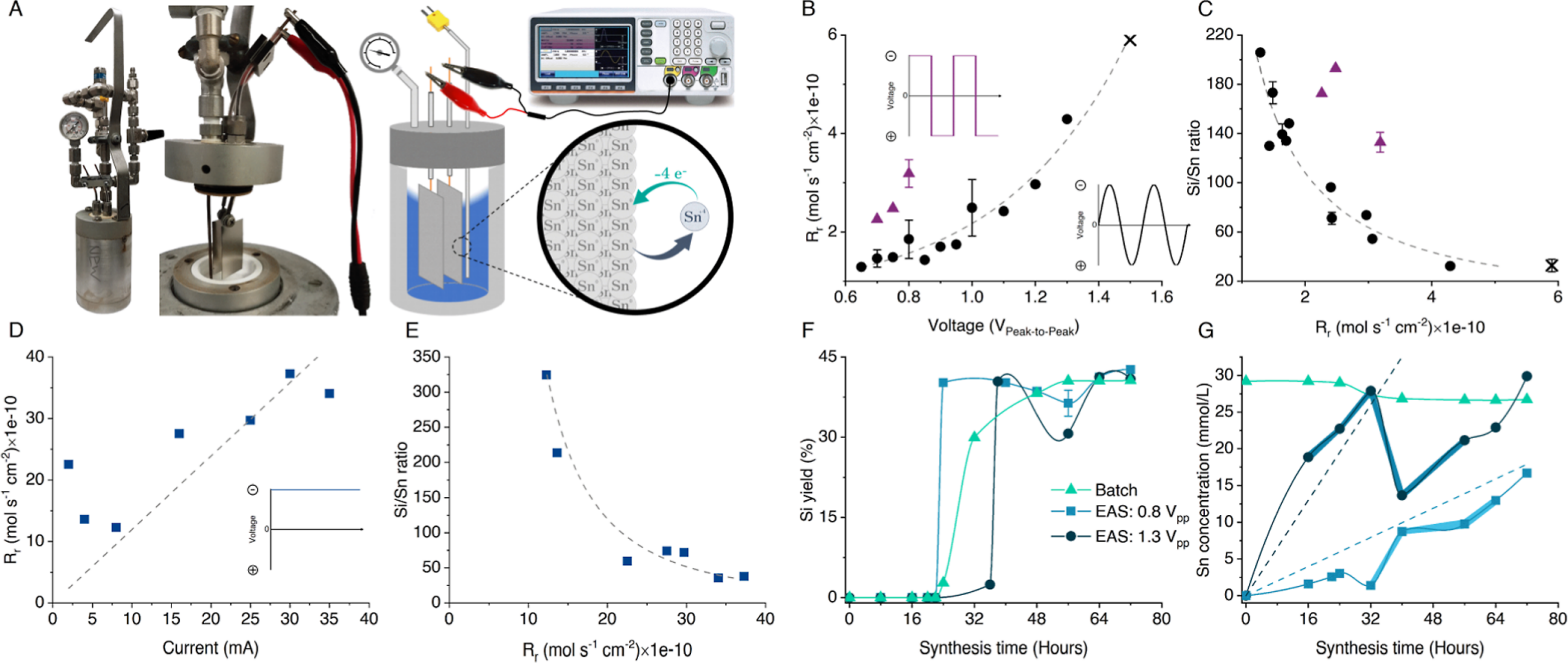
EAS reactor and the primary modes of metal release for incorporation control. (A) EAS reactor showing Sn-release (B) correlation between the applied voltage difference in AC mode for sine (dots) and square (triangles) waveforms and the release rate (*R*_r_) of tin. (C) The same *R*_r_ vs the final Sn-MFIs’ Si/Sn ratio. Crosses show the limits of precise Sn control when fewer firm results were obtained at higher V_PP_. (D) Correlation between applied current in DC/constant current regime and *R*_r_. Due to high *R*_r_, current was applied for 8 h after the first 16 h of synthesis (ST2.8). (E) The same *R*_r_ from D plotted against the final Sn-MFIs’ Si/Sn ratio in DC mode. (F) Sn-MFI crystallization curves in batch (Si/Sn 74) and EAS. (G) Ex situ measured tin concentration profiles for the two EAS syntheses from F and the calculated profile for the batch synthesis. Blue-banded areas on the EAS profiles show the experimental error (ST2.3) from both solid and liquid phase ICP–AES. Dashed lines show a theoretical Sn concentration based on average *R*_r_ values.

Using immersed electrodes in highly basic synthesis media within a temperature range of 80–160 °C and an autogenous pressure requires de novo reactor design (ST1). Figure 1A shows our modern custom-made reactor with voltage, pressure, and internal temperature control.

**Figure 2:**
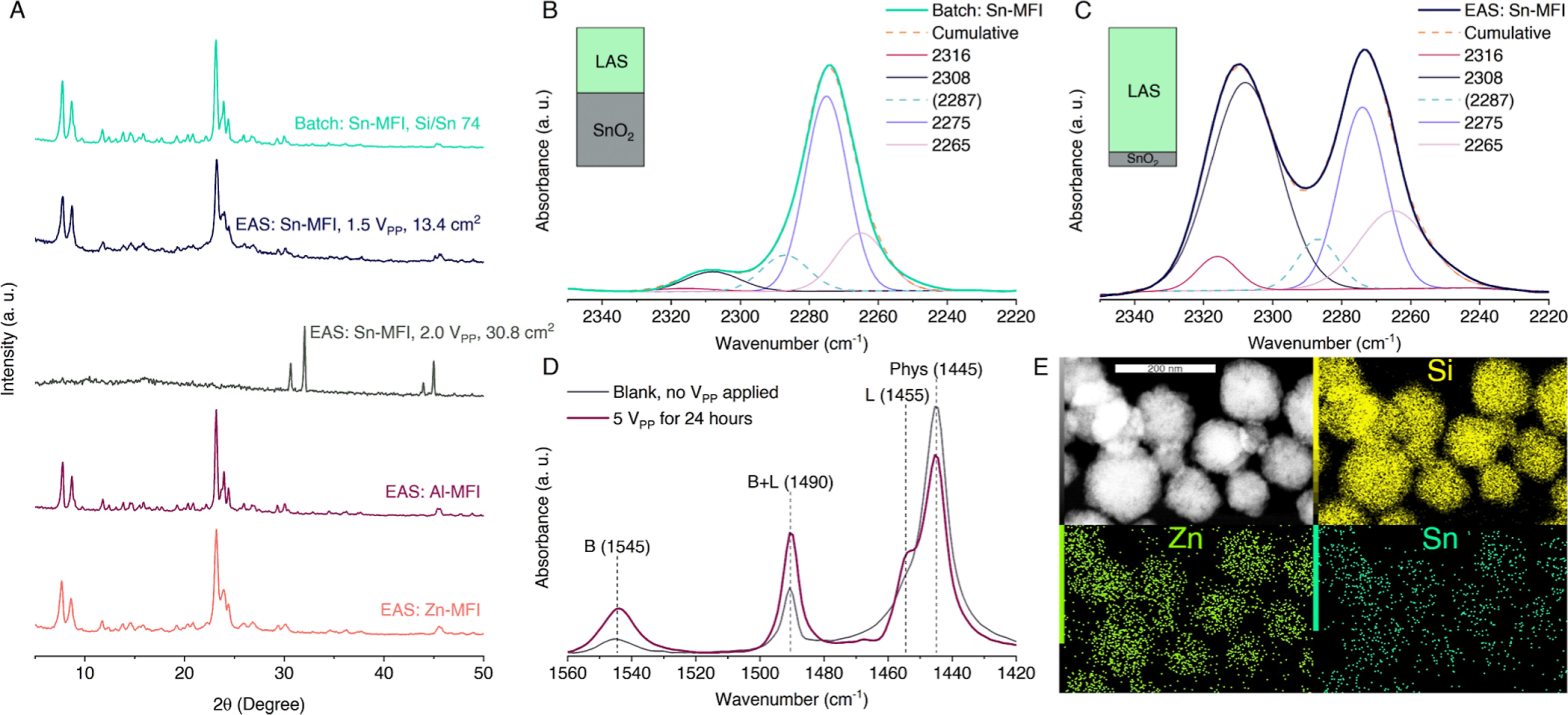
Generic nature of EAS for metallosilicate MFI zeotypes and their characterization. (A) PXRD patterns of Sn-, Al-, and Zn-MFI zeolites showing no structural, metal, or oxide impurities, vs a case with excessive Sn release (gray). CD_3_CN FT-IR on Sn-MFIs synthesized in (B) batch conditions (starting Si/Sn 74) and (C) via the EAS route with 1.5 V_PP_. (D) Pyridine FT-IR on Al-MFIs synthesized in the electro-reactor with and without applied voltage. (E) TEM–EDX images of the novel Zn,Sn-MFI zeotype. Embedded graphs in (B,C) and Figure 3B represent the percentage ratio of framework Sn (LAS) to extraframework Sn (SnO_2_) in each material. The real values are available in Table S11.

A proof of concept was conducted for Sn-MFI synthesis, a material with catalytic applications, such as lactate formation, that depends on tetrahedral Sn atoms (and more are wanted).^25,26^ The choice for Sn-MFI also hinges on the low temperatures and hydroxide media opposed to more tedious fluoride media syntheses common for stannosilicates. Additionally, anodic dissolution requires a conductive solution, mostly absent in dry-gel fluoride media. Benchmark experiments in classic batch autoclaves (Table S1) incorporated a low amount of tin into the resulting material, that is, product Si/Sn ratio of 281 for the lowest Si/Sn gel ratio of 74 (Powder X-ray Diffraction (PXRD)—Figure 2A, CD_3_CN Fourier-Transform Infrared spectroscopy (FT-IR)—Figure 2B).

Controlled tin release from an electrode surface in zeolite synthesis media is demonstrated for alternating bias (“AC”) modes in Figure 1B. The standard tin electrode potential hovers around *E*^0^(Sn^4+^/Sn^0^) = 0.245 V.^27^ This value corresponds to the most stable tin complex in aqueous solutions—Sn(OH)_6_^2-^, found in the high pH range according to the Pourbaix diagram.^28^ For materials synthesized with nominal voltages below or close to this value, that is, 0.6 V_PP_ (pp = peak-to-peak of the AC), no changes in electrode weight after hydrothermal synthesis were found, and resulting Si/Sn ratios were too large to be accurately measured (>Si/Sn 800, Table S2), that is, pure silica(li)te formed. The anodic tin dissolution expressed in release rate (*R*_r_) starts at 0.65 V_PP_ for the sine waveform (Figure 1B, black dots), as measured from electrode weight loss, and nonlinearly increases with V_PP_. The control is precise and reproducible, bearing in mind that an alternating bias was used here, with a very low frequency (50 μHz, 2.78 h for cathode/anode reversal), preventing electrodes from passivating or oxide building up, and, allowing both electrodes to release Sn. Plotting the V_PP_-controlled *R*_r_ versus zeolite synthesis outcome in Figure 1C reveals an excellent control over the Sn content in the final crystalline materials (PXRD—Figure 2A), which can surprisingly attain very low Si/Sn values (up to 32 for MFI with excellent LAS content: Figures 1B and 2C). We call this novel mode of control over metallosilicate heteroatom content “electro-assisted synthesis (EAS)”, where metal incorporation control only requires a push of the button. Moreover, the accessible Si/Sn ratio range is wider, as values lower than in the state-of-art are achieved.^29−31^ If the release is too great, tin concentration can reach a threshold that prevents (or heavily slows) zeolite formation, exposing EAS limits (e.g., ≥1.5 V_PP_). 2 and 4 V_PP_ tests (31 cm^2^, vs standard 13 cm^2^) corroborate this as no crystallization was seen and visible Sn^0^ particles formed in suspension (Figure 2A).^32^ We hypothesize that an excess of ionic tin can migrate and get reduced at the counter electrode.^33^

The second mode of EAS control entails the immersed surface. Experiments with increasing electrode area and fixed voltage show a linearly dropping release rate, thus behaving according to Faraday’s law of electrolysis. Going to larger surfaces slows down the rate per area, but overall, more Sn is released: comparing 22 cm^2^ electrodes to a 7 cm^2^ one, an absolute increase in the release of 42% was found, corresponding to a 49% Sn richer zeolite product (in-depth characterization: Table S2; Figure S5 and S17).

Further fine-tuning constant voltage modes for EAS is possible by using square waves or dedicated timing of releases. In essence, a sine function voltage bias partially dwells below the metal standard redox potential and, therefore, below an EAS onset value (ST3). Square waveform experiments at 0.7–0.8 V_PP_ (Figure 1B, purple triangles) demonstrate, compared to sines, a faster metal release since the bias is always above the threshold. On the contrary, the tin content in the formed zeolites was lower (Figure 1C) due to either too high heteroatom concentrations early on or no time to reach equilibrium tin saturation in the silicon matrix (constant release vs bursts in sine). To overcome this—next to working with sines—one can time the release only during certain crystallization stages (see “understanding EAS”).

Finally, using a constant direct current supply with variable voltage, Faradaic efficiencies were estimated in the range of 57–85% (ST2.5; Table S3). Due to high *R*_r_, the DC-controlled EAS is less straightforward with preserved trends but fewer correlations (Figure 1D,E). In contrast to electrochemical organic production processes (CO_2_ reduction,^34^ NH_3_ synthesis^35^), achieving high Faradaic efficiencies is quite irrelevant for inorganic material synthesis. The consumed EAS electrical energy is less compared to the power needed for heating (ST2.6) and the cost of ingredients. On top, zeolites are labeled “performance chemicals” as they have a huge leverage effect—2 orders of magnitude—for example, on producing (base) chemicals.^36,37^ We also surmise that the method has the potential to translate to larger scales as electroplating and metal electrolysis techniques are frequently encountered in industry in other domains.^38,39^

## 3 Understanding the EAS of Stannosilicates

A high tin content in a zeolite lattice is tough to achieve as the bigger atomic radius of tin disturbs its neighbors, demands a different T–O–T bond angle, and renders the structure less thermodynamically stable, on top of significant kinetic incorporation hurdles.^40^ Usually, these materials require longer synthesis times if they can overcome the thermodynamic barrier.^41^ Mechanistically, 3 explanations for the EAS success (controlled and record high Sn contents) were studied: concentration, reactive anodic species, and timing.

**Figure 3:**
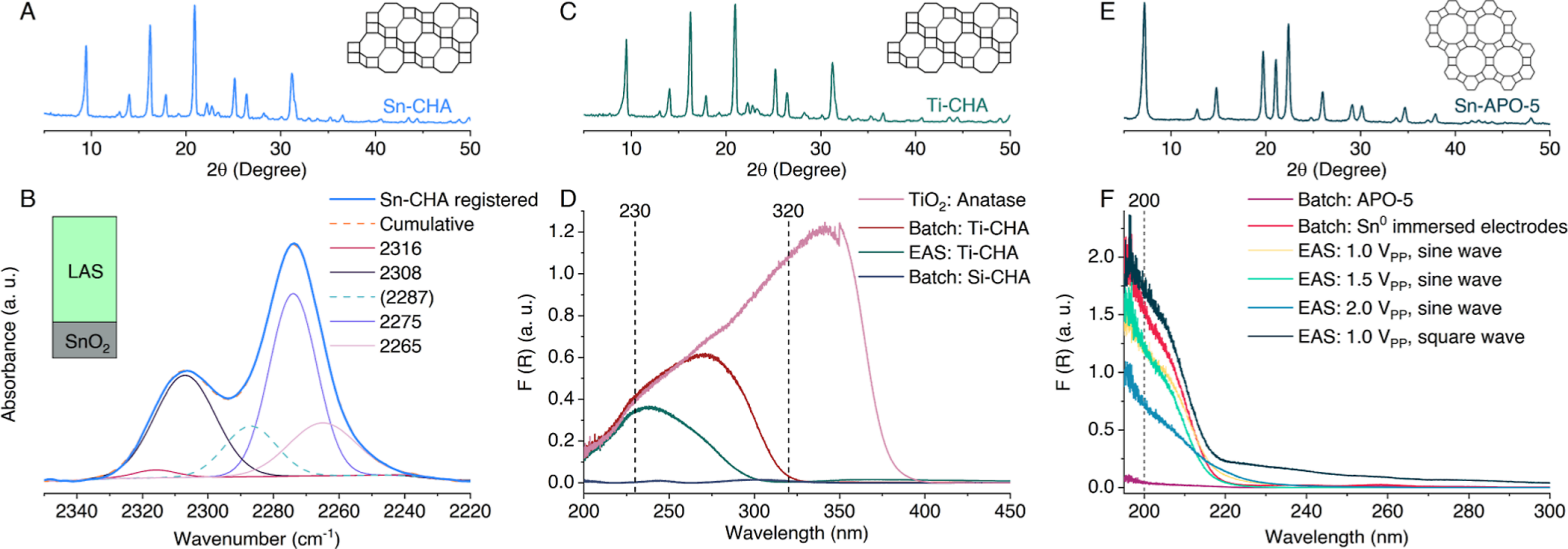
Generic nature of EAS for different topologies and their characterization. PXRD patterns of zeotypes synthesized with EAS: (A) Sn-CHA (24h-2hE-46h), (C) Ti-CHA (24h-2hE-46h), and (E) Sn-APO-5 (1 V_PP_, square wave). (B) CD_3_CN FT-IR spectrum of EAS Sn-CHA zeolite. (D) UV–vis–NIR spectra of as-made (Ti-)CHA zeolites crystallized in batch and EAS, and TiO_2_ and (F) of as-made (Sn-)APO-5 from batch and EAS.

The main difference between EAS and a classic batch synthesis lies in a tuned tin concentration profile. The MFI crystallization behavior of 2 EAS (Figure 1F) and 3 batch syntheses (siliceous, Si/Sn 74 and 149 from SnCl_4_·5H_2_O: Figure S3) was followed to discern nucleation, growth—where the steepest slope resembles maximal uptake of silica and tin—and maturation. While similar *S*-curves are found for EAS and batch, the initiation of crystallization, that is the end of the nucleation stage, appears at a different time as it highly depends on the amount of tin in a liquor.^17,42,43^ EAS offers a unique case as the tin concentration rises from zero, correlating with the average *R*_r_ (ST2.3; Figure 1G). The EAS concentration profiles are interrupted with a drop due to Sn-uptake (smaller in the batch profile) and then increase after. Crucially, while the tin concentration in EAS is always lower than in the batch case, the EAS zeolites are richer in tin, with Si/Sn ratios of 96 and 32 for 0.8 and 1.3 V_PP_, respectively, vs 281 in the batch. Thus, (i) EAS grants access to materials richer in heteroatoms than classic synthesis (here: up to a 10-fold); (ii) less tin precursor is wasted, so the metal efficiency (incorporated vs total soluble) is 7× higher; and (iii), there is less nucleation hindrance (in low concentration) rendering it a faster process.

Since a fast and reliable Sn-MFI with a high metal loading requires a rapid passing of the nucleation, the logical next step is postponing the release based on the siliceous *S*-curve, until right after nucleation, without irreversible crystallization disturbance. EAS was thus performed with 2 or 4 V_PP_ for 8 h (ST2.8) after 16 h of the Si-MFI induction period, yielding two zeolites with Si/Sn ratios of 77 and 45, respectively (Figure S19). Timing EAS saves energy and time, obtains Sn-richer zeolites, and increases tin efficiency, for example, from 5% in batch up to 58% (2 V_PP_).

The nature of in situ-released Sn-species is potentially different from that of hydrolyzed salt precursors, which often play a key role in the outcome.^44,45^ The rapid formation of a crystalline phase with a high Sn content, exclusive to EAS, hints at the release of uniquely active species from electrodes. Batch Si/Sn 125 experiments with ionic or pre-released anodically oxidized tin (ST2.7) did not lead to significant incorporation, resulting in Si/Sn ratios of 540 and 370 for SnCl_4_ and anodic Sn, respectively (Figure S20). Contrary to in situ release, these high ratios are either explained by the concentration effect or a short lifetime of “more active” species, which likely equilibrate fast. Still, these experiments are not a direct proof that the species delivered in EAS are unique or more active. While more evidence for having unique Sn species in EAS is required, the benefits of (and need for) in situ release are indisputable.

## 4 EAS: A Generic Concept for a Record Metal Incorporation

While stannosilicates are arguably the most valuable type of siliceous zeotypes, and MFI is a topology of utmost relevance,^36^ EAS could have a major impact if applicable to other metals and frameworks. The generic nature of EAS leading to metal-incorporated zeolite structures was proven by successfully synthesizing single metal Zn-, Al-MFI, a novel mixed Sn,Zn-MFI, as well as Sn-, Ti-CHA and Sn-APO-5. For all stannosilicates, the framework Sn content achieved is the highest recorded to date from bottom-up hydrothermal synthesis.

Based on well-known Zn-MFI syntheses in batch^46^ and similarities in the electrochemical dissolution between zinc and tin, the sine EAS procedure was performed with 0.8 V_PP_ using zinc electrodes. While the *R*_r_ of Zn is higher than that of Sn, easier incorporation of zinc into zeolite structures also leads to materials richer in metal. Counter to that, zinc in hydroxide media tends to form oxide/hydroxide precipitates, complicating the bulk zeolite elemental analysis when distinguishing these from framework zinc. Unique to EAS, a low metal concentration profile during crystallization and a constant supply can allow zinc to remain in a favorable state for incorporation. A closer look with TEM–EDX (Figure S24A and S25A) at nanocrystals from the low-temperature batch synthesis demonstrates the presence of a second zinc-rich impure phase, whereas the EAS crystals have a single phase with nicely dispersed Zn. A range of Zn-MFI syntheses (5 EAS, 8 controls) and their analysis by various techniques (ST2.9; Table S5; Figure S8, S24 and S25), for example, ion-exchange capacity,^47^ showed some benefits for EAS but also painted a complex picture in need of further investigation. The case for Zn by EAS entails targeting a decent zeolitic Zn content with better framework integration avoiding bulk zinc oxide formation.

Unique to EAS, one can obtain mixed metal-containing zeolites easily. A Sn-electrode and a Zn-electrode were simultaneously placed in the reactor, and a small voltage of 1 V_PP_ (ST2.9) was applied. The resulting pure zeotype had Si/Sn and Si/Zn ratios of 107 and 37, respectively, with both metals finely dispersed in the crystals (TEM–EDX—Figures 2E and S26). This first documented Sn,Zn-MFI hydrothermal synthesis (only post-synthetic Sn,Zn-BEA has been reported^16^) showcases the broad applicability of EAS, offering access to novel combinations and potentially revolutionary Lewis acidic catalysts.

Although aluminum demonstrated slight dissolution in hydroxide media without voltage, a timed EAS (ST2.10) successfully enriched the metal content in Al-MFI. The resulting Si/Al ratios of 143 (5 V_PP_) and 471 (no potential) correspond to the same Brønsted acid sites density increase: 3.1 and 10.9 μmol g^–1^ measured by pyridine FT-IR (Figure 2D). While Al-EAS can work, the release of ions from aluminum electrodes seems few as an oxide layer likely passivates it. We think the main dissolution happened on the non-submerged part of the electrodes (Figure S33), where water condensation took place, and anodic oxidation could occur at neutral pH. Similar issues (and less success) were encountered for iron (ST2.11). Further trials with Al-EAS in more neutral media are ongoing, although tons of classic recipes for aluminosilicates are available.

Large cages confined by small pores and commercial applications make CHA an object of interest.^48,49^ Nevertheless, a dry-gel conversion at 160 °C^5^ is known as the only route to Sn-CHA. Using EAS at 120 °C, we attained Sn-CHA with a little effort and high tin loadings. Two variations were tested: a dosed tin burst by 2 V_PP_ (square wave, 25 μHz) for 2 h, either at the start of the first or second day of synthesis, resulting in Si/Sn ratios of 57 and 68, respectively (PXRD—Figure 3A and S12; CD_3_CN FT-IR—Figures 3B and S21). A similar experiment with 4 V_PP_ at the start had a rapid tin release (*R*_r_ = 314 mol s^–1^ cm^–2^ × 1 × 10^–10^), producing an excessive tin supply, leading to an amorphous material (Si/Sn 14). Remarkably, lower incorporations, Si/Sn 204 and 127, were reached in batch for the chosen fluoride-containing synthesis path with SnCl_4_ and Sn^0^, respectively (Figures S12 and S21; Table S8).

A Ti-CHA attempt was made in the EAS Sn-CHA conditions, resulting in a highly crystalline material with the Si/Ti ratio of 62 (Figure 3C). However, thermochemical titanium dissolution in the batch blank sample led to Si/Ti 21 (Figure S12). In UV–vis–NIR spectroscopy, tetrahedral and octahedral titania units correspond to 230 and 320 nm bands. While EAS Ti-CHA is situated closer to the framework TiO_4_, the blank is more associated with anatase TiO_6_ (Figure 3D).^50^ Although EAS worked, we hypothesize that since the square wave was applied for a time shorter than the frequency shift, the surface of one electrode was blocked with a visibly evidenced oxide layer suppressing further thermo-electrochemical dissolution. A deeper investigation of such layers on dosing Ti via EAS is pending.

Broadening from silicates to substituted aluminophosphates, another class of molecular sieves, further widens the EAS applicability. Sn-APO-5 (topology AFI) was synthesized at 160 °C^51^ in the 1–2 V_PP_ range (PXRD—Figures 3E and S13), granting Al/Sn ratios of 37–81, whereas only slight thermal dissolution happened in the acidic condition (pH 5.1) for the blank sample—Al/Sn 171 (Table S9). Tin incorporation for all EAS materials was proven with UV–vis–NIR spectroscopy, where the 200 nm adsorption band suggests tetra-coordinated Sn (Figures 3F and S14). While classic precursors also work (ST2.12), these attempts strongly confirm the flexibility of EAS for synthetic purposes in neutral and slightly acidic media opposed to the high pH cases above.

These zeotype demonstrations show the versatility of EAS for tailor-made heteroatom-substituted materials by choosing an electrode, a siliceous mixture, and a mineralizing (structure-directing) agent. EAS is essential when media-driven dissolution is either less or uncontrollable (depends on the metal and the harshness of the conditions) and for materials where lower Si/metal ratios are required.

## 5 Catalytic Conversion of Triose Sugars

**Figure 4:**
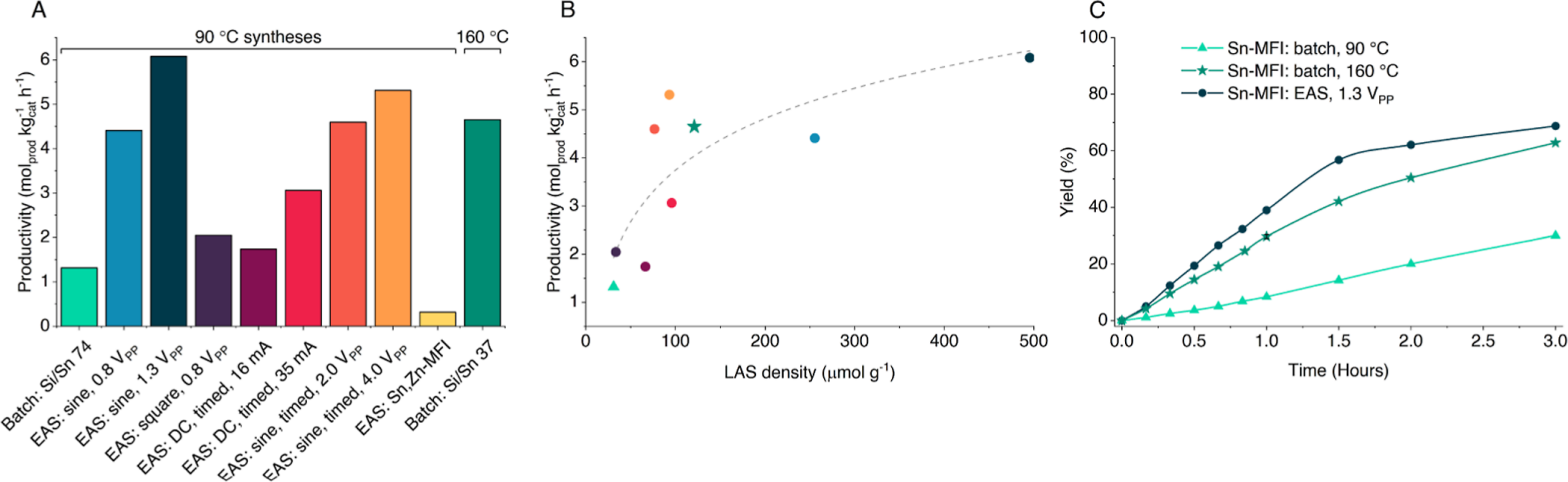
Catalytic performance of Sn-containing zeolites for triose sugar-to-lactates. Conditions: 80 °C, 80 mg of catalyst, 1.25 mmol DHA, 60 mg of naphthalene, 5 mL of CH_3_OH. (A) Catalyst productivity after 1 h of the reaction. (B) Correlation of productivity and framework tin content (LAS—see Table S11) in Sn-MFI zeolites with a guide to the eye. (C) Comparison of ML conversion kinetics over Sn-MFI zeolites.

To showcase increased densities of tin LAS in Sn-MFI zeolites, the conversion of 1,3-dihydroxyacetone (DHA) to methyl lactate (ML) was performed—a reaction where tin oxides are inactive.^26,52^Figure 4A shows that, within a series of samples synthesized at 90 °C, EAS Sn-MFI materials attain higher ML productivity than the mixed metal Sn,Zn-MFI zeolite, or the batch-crystallized Sn-MFI (batch Si/Sn 74, material Si/Sn 281). When we tried to double the amount of tin precursor in a 90 °C batch, no zeolite formed due to the prolonged (hampered) nucleation stage. Therefore, the temperature and time of synthesis were increased to 160 °C and 10 days, and an improved control zeolite was crystallized. This batch-synthesized Sn-rich MFI (batch Si/Sn 37, material Si/Sn 51) demonstrates a similar level of yield to EAS materials synthesized with a sine wave in classic or timed modes. The results can be explained by the tin content of synthesized zeolites (Figure 4B) and, more specifically, by an increased LAS density. For that, CD_3_CN FT-IR analyses (Table S11 and Figure S22; also less-well-resolved solid-state tin NMR: Figure S23) gave insights: for example, a poorer framework incorporation was found for EAS in DC mode, while a higher amount of tin LAS can be achieved with the AC gradual sine wave release. Remarkably, the optimal EAS Sn-MFI shows a better performance than any batch Sn-MFI zeolite (Figure 4C).

## 6 Future Prospects and Conclusions

An entirely new way of making heteroatom-containing zeolites is presented using a novel flexible electrochemical reactor. By performing anodic oxidation, finely controlled by a voltage bias on immersed electrodes, the timing, concentration, and speciation of heteroatom precursors can be tuned in situ during a zeolite crystallization, opposed to only a starting concentration in classic batch synthesis. This EAS allows the production of stannosilicates with record Sn incorporation by keeping tin concentration low and adjusting the release timing to avoid nucleation delays. The concept is generic as the synthesis of Sn-MFI (Si/Sn 32), the hard-to-realize Sn-CHA (62) and Sn-APO-5, was easily performed with very short synthesis times in hydroxide (and acidic) media. These Lewis acidic materials are renowned catalysts for a range of reactions and increasing their acid-site density is of key importance to their productivity. This was evidenced for lactate catalysis, and metal-rich EAS materials can now be explored for a range of reactions. Moreover, the genericity to other metals is shown by the release of Zn, Al, and Ti in the presence of zeolite growth, with framework metal incorporation. Interestingly, the method also opens up unprecedented access to mixed metal zeotypes (Zn/Sn), and one could even think about using timing, here, to access heteroatom zonation (core–shell) in zeolite crystals.

Combining zeolite synthesis with in situ anodic oxidation of metal precursors now enables a range of possibilities for creating advanced materials suitable for various catalytic and other applications. One can, for example, ponder incorporating heteroatoms via gentle EAS for nearly all siliceous syntheses reported. This is a new reactor-based and thus external handle for zeolite synthesis (a novel nonconventional method^53,54^), as opposed to the classic so-called internal “changing of ingredients” based handles. A road to infinite new zeotype and heteroatom content variations has been opened.

## 7 Experimental Section

### 7.1 Synthesis of MFI Zeolite in Hydroxide Media

The general recipe followed has been described in the literature.^55^

In a typical synthesis, 15.806 g of TEOS was mixed in a beaker with 19.276 g of TPAOH and 15.737 g of water. The mixture was stirred until a visibly homogeneous single-phase system is formed, and subsequently, if a procedure required metal from the start (batch) in the zeolite synthesis, a certain amount of metal (Me) salt (tin(IV) chloride, zinc acetate, aluminum nitrate, iron nitrate) was added. After that, stirring proceeded until full TEOS hydrolysis. Final batch compositions were
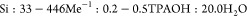


Afterward, the formed liquid gel was transferred into a Teflon-lined stainless-steel autoclave and placed in a convection oven at various temperatures for different times. After crystallization and cooling down, a zeolitic product was separated from a cloudy solution or two-phase mixture by centrifugation (6200 rpm, 5–30 min), washed with Milli-Q water until pH was below 10 with the same method (centrifuge/wash with approx. 50 mL g^–1^), and dried overnight at 60 °C. Dried products were calcined in static air with the following program in a muffle oven: a slow temperature increase from ambient until 550 °C taking 9 h after which it was kept isothermal for 6 h at 550 °C.

A Si/OSDA ratio of 0.2 was used only for synthesis of Si-MFI support for zinc-impregnated samples (Figure S15 and Table S10).

### 7.2 Synthesis of Si-CHA Zeolite Seeds in Fluoride Media

The procedure has been described elsewhere.^56^

In a typical synthesis, 17.186 g of TEOS and 43.559 g of TMAdamOH were mixed together and kept under vigorous stirring. Later, when the two-phase system reached homogeneity, the mixture was stirred constantly at room temperature for the complete TEOS hydrolysis and evaporation of ethanol and water to achieve the batch composition of
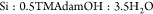
at the end of the full procedure. After evaporation, 2.064 g of HF (Caution: using HF is very dangerous, use adequate safety precautions) was added dropwise as a mineralizing agent in an equimolar amount to OSDA; at the end, the obtained dry gel was milled in a mortar to attain fine particles. Finally, the gel was transferred into a Teflon-lined stainless-steel autoclave, which was kept in a conventional oven at 160 °C for 48 h. After crystallization, the product was treated as described for MFI.

### 7.3 Synthesis of CHA Zeolite in Fluoride Containing Hydroxide Media

The procedure has been described elsewhere.^56^

In a typical synthesis, 8.860 g of (NH_4_)_2_SiF_6_ was mixed in a beaker with 26.258 g of TMAdamOH and 5.850 g of Milli-Q water. After 30 min of vigorous stirring, 11.956 g of EDA was added, causing exothermic heating of the mixture. Another 30 min later, if the synthesis procedure required the addition of tin(IV) chloride, it was added to the solution. At last, after another 30 min, 0.149 g of Si-CHA seeds was added and then the mixture was stirred for another 3 h prior to the synthesis (the seed step is essential for getting a crystalline product—no crystalline phase was found when the stirring ran for less than 3 h). The final batch composition was
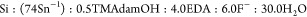


Afterward, the formed liquid gel was transferred into a Teflon-lined stainless-steel autoclave and placed in a conventional oven at 120 °C for 72 h. After crystallization, the product was treated as described for MFI.

Remarkably, the synthesis gel has a big concentration of fluoride ions (which probably assist in the material formation). It caused degradation of some internal metal parts of the electro-reactor.

### 7.4 Synthesis of Sn-APO-5 Aluminophosphate in Acidic Media

The procedure was described elsewhere.^51^

In a typical synthesis, 1.346 g of H_3_PO_4_ was mixed in a beaker with 10.469 g of Milli-Q water. After 10 min of stirring, 0.861 g of aluminum oxide was added, and the system was left under vigorous stirring for 30 min. Then, 0.092 g of tin(II) chloride or 0.127 g of tin(IV) chloride was added to a solution if the synthesis procedure required. At the last step, 1.900 g of *N*,*N*-dicyclohexylmethylamine was added, and the system was left for 30 more min to reach a homogeneous gel. The final batch composition was
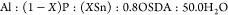
where *X* equals 0.04 in the case of added tin salt. Afterward, the formed liquid gel was transferred into a Teflon-lined stainless-steel autoclave and placed in a conventional oven at various temperatures and times. The product was treated as described for MFI.

### 7.5 Conducting Anodic Metal Oxidation Experiments in the Reactor for EAS

For a synthesis of metal-containing zeolites with various frameworks using the anodic metal oxidation technique, a small excess of a pure-siliceous gel was prepared (recipes above, without metal salt addition). For a general experiment, 34 g of a liquid gel was placed into a 50 mL Teflon-lined custom-made stainless-steel autoclave. Before an experiment, two (in most cases identical) metal plate electrodes (Sn^0^, Zn^0^, Fe^0^, Ti^0^, Al^0^) with a required surface area were washed in acetone, dried, weighed, and attached to the internal pair of nut connectors. Afterward, the EAS reactor was closed and put into a heating mantle at room temperature. In the end, the pair of terminals were attached to the wire connectors on the top part of the custom-made reactor. The synthesis starting time was counted from 60 °C (about 30 min of heating) indicated on the controlling thermocouple. The electricity was turned on simultaneously (time 0) or after several hours/days for a required period (“timing EAS”). Voltage, frequency, and waveform, the crucial parameters of an EAS, were set before the start of an experiment on all electric signal generators. A multimeter is sometimes used to check connectivity. After crystallization, the reactor was cooled in an ice bath and then opened; electrodes were washed in a flow of distilled water, dried, and weighed; a solid zeolitic product was separated from a cloudy solution or two-phase mixture by centrifugation (6200 rpm, 5–30 min), washed with Milli-Q water until pH was below 10 with the same method (centrifuge/wash with approx. 50 mL g^–1^), and dried overnight at 60 °C. For the concentration profiles, a liquid phase was collected after centrifugation without dilution for a further Inductively Coupled Plasma Atomic Emission Spectroscopy (ICP–AES) analysis. Dried products were treated as described for MFI.

For a general experiment, if not specified, a frequency of 50 μHz was applied.

### 7.6 Catalysis

Catalytic conversion of DHA in methanol to ML was performed under autogenous pressure in 10 mL thick-walled glass reactors placed in a preheated metal block at 80 °C. Typically, 0.1126 g of DHA, 80 mg of catalyst, 60 mg of naphthalene (internal standard), and 5.0 mL of methanol were added to a test vial and sealed with a crimp cap. The vessel was then put in the metal block with oil between the glass and metal for heat transfer and vigorously stirred for several hours, followed by quenching in an ice bath for sampling. Products were quantified through gas chromatography–flame ionization detection analysis using a calibration curve with commercial compounds. The gas chromatograph was an Agilent 6890 (Split) with an HP-5 capillary column (30 m × 0.32 mm; film thickness 0.25 μm). Nitrogen was used as carrier gas at a 1 mL/min flow rate. The samples were injected using a split/splitless injector at 523 K with a split ratio of 1/25. The oven temperature program was 333 K, hold for 2 min; ramp to 453 K, rate: 5 K min^–1^; ramp to 543 K, rate: 20 K min^–1^; 543 K hold 3 min. The detector’s temperature was set to 553 K.

## 8 Characterization Methods

### PXRD

PXRD data were collected on a high-throughput STOE STADI P Combi diffractometer in the transmission mode with focus on Ge(111) monochromatic X-ray inlet beams (λ = 1.5406 Å, Cu Kα source).

### Nitrogen Adsorption Isotherms

Nitrogen adsorption isotherms in the relative pressure range of 0–1 bar were measured on a TriStar II 3020 instrument. All the samples were dried at 300 °C for 6 h prior before analysis. The textural properties of all samples were then obtained from the adsorption isotherms. The Brunauer–Emmett–Teller specific surface area was calculated according to the Rouquerol method.^57^ The micropore volume (*V*_mic_) was deducted from the *t*-Plot method.

### ICP–AES

ICP–AES analyses were conducted on a PerkinElmer Optima 3300 DV with signals for Si, Al, Sn, Fe, Zn, Ti, Ni, K, P at 251.6, 380.2, 189.9, 238.2, 213.9, 334.9, 231.6, 766.5, and 213.6 nm, respectively. Samples for the analysis were prepared via 3 different methods:

1. HF method (for all elements, except tin): Before an ICP–AES measurement, 10 mg of samples was dissolved using 0.4 mL of HF and 0.2 mL of aqua regia. After 3 h, they were neutralized using 5.0 mL of boric acid and diluted with 14.4 mL of Milli-Q water. After an additional 2 h, samples were diluted using 0.42 M HNO_3_ in water by a factor of 10. Caution: using HF is very dangerous, use adequate safety precaution.
2. Oven method (mainly for tin): 24 h prior to an ICP–AES measurement (important as silicon tends to sediment from an acidic solution), 50 mg of samples was mixed with 250 mg of lithium borate. Next, the mixture was placed in a graphite crucible and held inside a muffle oven for 10 min at 1000 °C. Then, the formed still hot droplet was transferred to 10 wt % HCl solution in water and stirred for 10 min. After an additional 2 hours, the sample was diluted using the HCl solution of the same concentration by a factor of 5.
3. Liquid fraction analysis (for concentration determination of a released metal after EAS): liquid fraction was separated from a solid product by centrifugation, and later, prior to an ICP–AES measurement, it was diluted using 0.42 M HNO_3_ or 10 wt % HCl (for tin measurement) by a factor of 500.

### FT-IR Spectroscopy

Active acid sites of zeolites were analyzed by FT-IR spectroscopy (Nicolet 6700 spectrometer equipped with a DTGS detector). Samples were pressed into self-supported wafers and degassed under a vacuum at 400 °C for 1 h prior to the adsorption measurements.

#### Pyridine Adsorption

After cooling the cell to 50 °C, 5 mbar of gas-phase pyridine was introduced to the sample cell until saturation. Thermal desorption was performed at 150 °C for 20 min in order to remove the weakly adsorbed pyridine species. The amount of Lewis and Brønsted acid sites was quantified by integrating the area of the absorption bands at 1455 and 1545 cm^–1^, respectively. For Al-MFI zeolites, the absorption extinction coefficients determined by Emeis were used: ε(*B*) = 1.67 cm μmol^–1^ and ε(*L*) = 2.22 cm μmol^–1^.^58^

#### Acetonitrile Adsorption (Titration)

After cooling the cell to 30 °C, small dosing on a pellet was performed by introducing 5 mbar of gas-phase deuterated acetonitrile to the sample cell. After 8 doses of titration, the pressure was changed to 10 mbar and later to 20 mbar. After the appearance of the 2265 cm^–1^ band assigned to the physically adsorbed CD_3_CN, a sample was saturated with the probe molecule for 5 min. The desorption was performed at 30 °C for 5 min in order to remove the weakly adsorbed acetonitrile species. For Sn-containing zeolites, the spectra were deconvoluted in two types of framework-incorporated Sn sites (2316 and 2308 cm^–1^), SnO_2_ (2287 cm^–1^), silanol groups (2275 cm^–1^), and physisorbed or gas-phase CD_3_CN (2265 cm^–1^) with OriginPro software. All bands were fitted using the Gaussian peaks form. For all deconvolutions, a peak center variation of ±3 cm^–1^ was allowed, and fwhm values ranged from 10 to 25 cm^–1^. For Sn-containing zeolites, the molar extinction coefficients for adsorbed acetonitrile determined by Harris et al. were used: ε(2316) = 1.04 cm μmol^–1^, ε(2308) = 2.04 cm μmol^–1^, ε(2287) = 2.13 cm μmol^–1^, and ε(2275) = 0.74 cm μmol^–1^.^59^ Framework tin or LASs were calculated as a sum of both framework-incorporated tin sites bands (2316 and 2308 cm^–1^). Embedded graphs on Figures 2B,C and 3B show a calculated percentage ratio between LAS and extra framework SnO_2_; the calculated CD_3_CN adsorption values can be found in Table S11.

### Diffuse Reflectance Spectroscopy

Diffuse reflectance spectroscopy in the UV–vis–NIR energy range (UV–vis–NIR) was performed with a Varian Cary 5000 UV–vis–NIR spectrophotometer equipped with the internal DRA 2500 accessory at room temperature against a Halon white reflectance standard in the 4000–57000 cm^–1^ energy range.

### Scanning Electron Microscopy (SEM)

SEM was performed on the JEOL JSM-6010LV microscope at an acceleration voltage of 15 kV. Zeolite samples were attached to a piece of carbon tape. Subsequently, a thin Pd/Au (60/40 ratio) layer was deposited on top of the samples to achieve sufficient conductivity.

### Transmission Electron Microscopy (TEM)

TEM of the samples was performed with an aberration-corrected JEOL ARM200F microscope operating at an acceleration voltage of 200 kV and equipped with a cold field emission gun. Dark-field imaging was performed in scanning TEM mode with an annular dark-field detector. Energy-dispersive X-ray (EDX) spectroscopy analysis of Si, O, Sn, and Zn in the samples was carried out utilizing a Centurio EDX detector with a solid angle of 0.98 steradians from a 100 mm^2^ detection area. The samples were prepared via drop-casting a sonicated particle suspension on a holey carbon-coated TEM grid (Cu, 400 mesh, Agar Scientific).

### ^119^Sn Solid-State NMR

^119^Sn solid-state NMR was measured using a Bruker 500 MHz NMR spectrometer and a Bruker 4 mm MAS probe. For dehydration prior to the experiment, a sample was packed into a 4 mm rotor and evacuated at 200 °C for 2 h. Dry oxygen gas was introduced to boost ^119^Sn spin relaxation time and sealed by a kel-F cap tightly before the MAS NMR experiment. The MAS NMR signal was averaged over 5000–10,000 scans with recycle delay time of 10 s. The spinning rate was 12 kHz.

### pH

pH was measured with a Mettler Toledo SevenCompact pH meter with a polymeric InLab Expert Pro-ISM pH electrode. Before measurement, the electrode was calibrated with InLab technical buffer solutions (Mettler Toledo). After each measurement, the electrode was rinsed with Milli-Q water (ρ = 18.2 MΩ cm^–1^, synergy UV purification system). Subsequently, the pH was measured, and the electrode was stored afterward in an InLab KCl storage solution.

## Supporting Information Available

Materials, additional methods, detailed experimental guidelines for syntheses of zeolites and the EAS procedure, and additional in-depth characterization (PDF)
