# Supplementary material for: Engineering Lewis Acidity in Zeolite Catalysts by Electrochemical Release of Heteroatoms during Synthesis
Source: Chem Mater. 2023 Jun 1;35(13):5049–58. doi: 10.1021/acs.chemmater.3c00552 (PMC10339459; doi:10.1021/acs.chemmater.3c00552)
Supplement: Supplementary file 1 — cm3c00552_si_001.pdf [file cm3c00552_si_001.pdf]

Supporting Information

Engineering Lewis acidity in zeolite catalysts by electrochemical release of heteroatoms during synthesis

*Gleb Ivanushkin, Michiel Dusselier**

Table of Contents

Table of Contents 2
Commercial chemicals and materials: 3
Supplementary Text 4
ST1. Set-up (de novo reactor design) for conducting an anodic metal oxidation experiment 4
ST2. A detailed description of EAS experiments 5
ST2.1. Used generators of an electric signal 5
ST2.2. Blank experiments with pure metals as precursors 5
ST2.3. Calculation and measuring tin concentration inside a reactor during an experiment 5
ST2.4. EAS controlled with an area of electrodes 7
ST2.5. Calculation of Faradaic efficiency 7
ST2.6. Energy efficiency calculation for metal-containing zeolites synthesis via EAS route 8
ST2.7. Experimental design for ex-situ tin precursor preparation by anodic oxidation 8
ST2.8. Experiment design for more efficient tin incorporation by the right timing of the metal release 9
ST2.9. EAS of Zn-MFI and its characterization 10
ST2.10. EAS of Al-MFI 11
ST2.11. EAS of Fe-MFI 11
ST2.12. EAS of Sn-APO-5 12
ST3. Time a sine spends below a threshold value, and, ‘average voltage’ for EAS 13
Figures 14
Tables 54
References 68

Commercial chemicals and materials:

Teraethoxisilane or TEOS [$Si(OC_2H_5)_4$, Fischer Scientific, 99+%]
Ammonium hexafluorosilicate [$(NH_4)_2SiF_6$, Fischer Scientific, 98%]
Aluminum oxide [Al_2O_3, Sasol, PURAL SB]
N,N,N,N-tetrapropylammonium hydroxide or TPAOH [$(n\text{-}C_3H_7)_4NOH$, Sachem, 40 wt. % in water]
N,N,N-trimethyl-1-adamantanammonium hydroxide or TMAdamOH [$(CH_3)_3C_{10}H_{15}NOH$, Sachem, 20 wt. % in water]
Ethylenediamine or EDA [$C_2H_8N_2$, Sigma-Aldrich, >99.5%]
N,N-Dicyclohexylmethylamine [$(C_6H_{11})_2NCH_3$, Merck, 97%]
1,3-dihydroxyacetone [$C_3H_6O_3$, Fisher Scientific, >98%]
Methanol [CH_3OH, Acros Organics, 99.9%]
Potassium chloride [KCl, Chem-Lab Analytical, 99.5+%]
Nickel nitrate hexahydrate [$Ni(NO_3)_2 \times 6H_2O$, Sigma-Aldrich, >94.5%]
Lithium metaborate [$LiBO_2$, Sigma-Aldrich, 99.9%]
Tin chloride dihydrate [$SnCl_2 \times 2H_2O$, Honeywell Fluka, >98%]
Tin chloride pentahydrate [$SnCl_4 \times 5H_2O$, Sigma-Aldrich, 98%]
Metal tin powder [Sn, Acros organics, 99.5%]
Metal tin foil [Sn, thickness 0.125 mm, Good Fellow, 99.9%]
Metal titanium foil [Ti, thickness 0.127 mm, Sigma-Aldrich, 99.7%]
Aluminum nitrate nonahydrate [$Al(NO_3)_3 \times 9H_2O$, Chem-Lab Analytical, 99.5+%]
Metal aluminum foil [Al, thickness 0.25 mm, Fisher scientific, 99.997%]
Iron nitrate nonahydrate [$Fe(NO_3)_3 \times 9H_2O$, Fisher scientific, >98%]
Metal iron foil [Fe, thickness 0.25 mm, Good Fellow, 99.5%]
Zinc acetate dihydrate [$Zn(CH_3COO)_2 \times 2H_2O$, Acros Organics, 98%]
Zinc oxide [ZnO, Fisher scientific, >95%]
Metal Zn foil [Zn, thickness 0.25 mm, Alfa Aesar, 99.98%]
Boric acid [H_3BO_3, Chem-Lab, 99.5+%]
Orthophosphoric acid [H_3PO_4, Acros Organics, 85 wt. % in water]
Nitric acid [HNO_3, Acros Organics, 65 wt. % in water]
Hydrochloric acid [HCl, Acros Organics, 37 wt. % in water]
Hydrochloric acid [HCl, Fischer Scientific, 0.1 M standard solution in water]
Hydrofluoric acid [HF, Sigma-Aldrich, 40 wt. % in water]
Milli-Q water

Supplementary Text

ST1. Set-up (de novo reactor design) for conducting an anodic metal oxidation experiment

In Figure 1A, the de novo reactor design is depicted. Inside the main aluminum body, made from Teflon, a lid and a liner are placed. This particular isolation is necessary for the internal electrochemical cell construction. In previous designs of the set-up, it was found that the system with a metal lid tends to cause a short circuit because of the formation of a water bridge between electrodes across the metal lid (integrated with the reactor head). The Teflon lid-head has several holes for connections: thermocouple, pressure gauge, and two more for electrode connectors, made from copper (Stainless steel construction doesn't have a proper level of conductivity). A voltage difference is set on a pair of electrodes by connected terminals from a Function Generator (AC mode) or a Power Supply (DC mode). Waveform or frequency can be changed besides usual voltage control or set point current in DC mode. A Parr heating mantel controls the heating of the set-up with 2 thermocouples: probe and control used to prevent overheating.

Remark: over time, the O-ring in between the reactor head and the Teflon liner deteriorates (even Kalrez ®) and does not hold the pressure created during a high-temperature synthesis (>100 °C), it should be replaced then if this happens, a small leakage in the moment of cooling could happen, which could bring a tiny amount of aluminum (which comes from the main body) inside the reactor.

ST2. A detailed description of EAS experiments

ST2.1. Used generators of an electric signal

Three different types of electricity generators were used in the current work:

- Multi-Channel Function Generator: GW Instek MFG-2200 (MFG)
- Stanford Research Systems Model DS335 (SRS)
- DC Power Supply: Tenma 72-13330

MFG and SRS were used for EAS experiments providing a signal in a sine and square waveform. The DC power supply was used for constant current experiments and Faradaic efficiency calculation. A more specified description of synthesized materials with each type of generator is given in Table S12.

ST2.2. Blank experiments with pure metals as precursors

Dissolution of electrodes due to aggressive media or temperature-related origins was checked via a blank experiment conducted in a classic batch reactor or the electro-reactor. In the case of a batch reactor, in order to keep the liquor-to-metal surface ratio constant (32 ml vs. 13.4 cm^2), only one electrode with a standard area (6.7 cm^2) was placed inside since the volume is two times smaller (15 ml). A blank EAS was run in the electro reactor with attached electrodes but without applied (voltage) potential difference.

For Sn-CHA, the blank experiment in the classic batch reactor was conducted with a deficiency of synthesis liquor: 5.5 ml vs 6.7 cm^2, and crystalline material with Si/Sn 75 was reached, which obviously reveals a partial electrode dissolution and its incorporation in Sn-CHA material during crystallization. Again, after the blank EAS synthesis, the Sn-CHA zeolite was crystalline but had higher Si/Sn content 127 (PXRD patterns – Figure S12).

A comparison of these two blank methods leads to an insight into EAS application in the situation when electrode dissolution can happens inadvertently thermochemically. Firstly, the liquor-to-metal surface ratio should be appropriately chosen to result in a crystalline metal-incorporated material. Secondly, if the metal incorporation limit in the material lattice was not yet met, in-situ anodic oxidation could supply it additionally, which helps with the synthesis of more metal-rich materials to the edge of their capacity.

EAS is especially essential in situations when dissolution is nonexistent (which clearly depends on harshness (pH, F-presence, Temperature) of the synthesis liquid; or for materials where a much higher Si/Me ratio are required.

ST2.3. Calculation and measuring tin concentration inside a reactor during an experiment

For building tin concentration profiles (Figure 1G), its concentration inside of a reactor was calculated. The reactor was loaded with the same freshly prepared zeolite precursor mixture for each experimental point. After a few hours of crystallization, a reaction was stopped, and products were separated, washed, and analyzed in the way described, e.g., for MFI hydroxide synthesis.

- Classic batch reactor:

For each sample of the S-crystallization curve (Figure 1F), a silicon yield and the Si/Sn ratio of solid were measured. The final tin concentration, i.e., the remaining amount of tin in the solution, is established as the starting point is known, and the tin uptake of a solid was determined by ICP-AES for solid material combined with its yield.

$$[Sn]_\tau = [Sn]_0 - R_{zt} \times \frac{\tau}{V},$$

where $[Sn]_\tau$ – concentration of tin at a certain period of time expressed in mmol L^{-1}, $[Sn]_0$ – starting (batch) tin concentration, R_{zt} – 'rate of zeolitic tin', i.e. tin uptake by zeolite framework per time:

$$R_{zt} = \frac{mole\ of\ formed\ solid\ Si\ matrix}{(\frac{Si}{Sn}\ ratio\ ICP) \times \tau},$$

τ – time of crystallization, V – volume of a reaction mixture.

The values of R_{zt} evaluated at a local (instantaneous) time is not meaningful per se, as it is an average rate measure over the full synthesis time up to the stop of the EAS experiments. For each EAS experiment (both curves at 0.8 and 1.3 V_{PP}), tin concentration was measured ex-situ as follows:

The amount of dissolved tin (concentration) at a point in time is considered from the sum of electrode weight loss minus tin uptake by a solid phase, the latter calculated by ICP-AES and yield:

$$[Sn] = \frac{(R_r * S - R_{zt}) \times \tau}{V},$$

where R_r – release rate of tin, normalized by the area of electrodes, at the point analyzed (each time a separate experiments, where a different time provides a measured R_r and R_{zt}. The volume is always considered constant (31.8 ml, from 34 g of gel with 1.07 g ml^{-1} density).

$$R_r = \frac{\Delta m}{M \times S \times \tau} = \frac{mole\ of\ dissolved\ tin}{S \times \tau},$$

where Δm – electrodes (both) weight loss, M – molar mass of electrode material, S – area of electrodes.

The R_r values in figures 1B, C, D and E are calculated in the same way, but only analyzed after the full synthesis time and thus an average for the whole synthesis. The values used in the EAS tin concentration measurements are analyzed for each point (Figure 1G). Furthermore, the two dashed lines in Figure 1G are based on the average R_r of all these points for a given V_{PP}. These values corresponds well with the full synthesis time R_r values in Figure 1B, C, D, E. The dashed lines nicely approximate the concentration profiles for EAS and indicate that assuming a fairly constant R_r over the whole synthesis time is close to reality.

Using the differentiation operation on concentration profiles, another indirect characteristic can be determined for zeolite synthesis stages. For the classic batch synthesis profile, $\frac{\partial[Sn]}{\partial t} = 0$ during nucleation and growth stages, whether during the growth phase of the crystallization, when tin is consumed, $\frac{\partial[Sn]}{\partial t} < 0$. In the case of the EAS profile, it also becomes negative during crystallization. However, $\frac{\partial[Sn]}{\partial t} > 0$ during the others phases of the S-curve, as the concentration of tin gradually increases due to the electrochemical reaction on the surface of electrodes. A typical ('kink') drop in the concentration profile in EAS clearly shows the very fast growth phase of the S-curve.

To verify the Sn concentration profile (measured + calculated) from R_r and R_{zt}, the actual tin concentrations of the liquid was measured directly by ICP-AES of the separated liquid phase. The values were in close agreement, and the pale-colored bands in Figure 1G are based on error bars determined from combining the values of the R_r+R_{zt} method and the liquid phase method. The points in Figure 1G itself are the ones from the R_r+R_{zt} method, to be directly comparable to the batch experiment values.

Note that R_r values cannot be compared across different synthetic systems (e.g. MFI and CHA) directly, as the R_r could be partially influenced by the synthesis mixture compositions, and especially the nature of the base used, pH, ionic strength, viscosity and ionic metal concentration (cfr. Nernst Planck equation).

ST2.4. EAS controlled with an area of electrodes

For the series of experiments with a different electrode area, electrode pairs were cut from a sheet of tin foil with dimensions 0.5 × 4.0, 1.0 × 4.0, and 1.3 × 5.0 cm. As nut connectors have a solid position in the reactor's hat, and the volume of added siliceous precursor gel was kept unchanged, these electrodes had an immersed area of 6.7, 13.4 (standard), and 22.6 cm^2, respectively. An EAS was conducted at 90 °C with the following characteristics of electric waveform: 0.8 V_{PP}, 50 μHz, 72 hours, sine wave.

Faraday's first law of electrolysis can be expressed in

$$\Delta m = k \times I \times \tau \; and \; with \; j = \frac{I}{S}, \Delta m = k \times j \times S \times \tau,$$

where Δm – weight of the dissolved metal, k – electrochemical equivalent, I – current, τ – time, j – current density, S – area of an electrode, M – molar mass of electrode material (later equation). After performing a few mathematical operations, the R_r parameter can be written in another form:

$$\frac{\Delta m/M}{S \times \tau} = R_r = \frac{k \times j}{M}, \qquad R_r \sim j \sim \frac{1}{S}$$

The area of electrodes is a valuable parameter because an electrochemical reaction takes place on the border of solid and liquid phases. Therefore, a lower current density will be obtained for an electrochemical dissolution, if a wider area of an electrode is chosen. Current density thus correlate with R_r which is normalized by area. However, more Sn will be dissolved from a larger electrode area, due to a higher overall dissolution rate (not area normalized) resulting in a material with higher metal content (Figure S6).

ST2.5. Calculation of Faradaic efficiency

For calculating Faradaic efficiency, a series of timed experiments for EAS of Sn-MFI was conducted, similar to ST2.8. The constant current mode of the DC Power Supply was used instead of the SRS generator. In a usual experiment, a pure siliceous gel was loaded in a Teflon reactor with two Sn-made electrodes. After a reactor was closed and kept under reaction temperature for 16 hours, terminals were connected, and DC electricity was applied in the constant current mode (CC). After 8 hours of electrochemical reaction, electricity was turned off, and terminals were disconnected; a sample was held for further crystallization for 48 hours (72 hours in total). After the synthesis, the formed solid product and liquid phase were collected, and tin concentration in both phases was defined by ICP-AES analysis. The applied signal and voltage was written down by the PC, which allowed to establish an amount of current passed through the system (Figure S2).

Faradaic efficiency was calculated from the formula:

$$F_{eff} = \frac{C_{Sn} \times V_{sol} \times z \times F}{I \times t} \times 100\%$$

where C_{Sn} – tin concentration (sum of released Sn, i.e. Sn concentration in liquid and that taken up in the solid product (correct to a concentration); V_{sol} – a volume of loaded siliceous gel, z – a number of electrons spent for a reaction (the reaction of Sn^0 to Sn^{4+} was considered [z=4]); F – Faradaic constant; I – applied constant current; t – time.

Faradaic efficiency for the anodic oxidation of tin was calculated in the range of 57 – 85 % (Table S3). These numbers of Faradaic efficiency are obtained from a high voltage applied for generating a current threshold value. Running constant current experiments require variable and quite high voltages, which causes a set of electrochemical side reactions such as water splitting and likely OSDA oxidation (darker color of solution after synthesis) to occur. For EAS, constant voltage modes are clearly better, but there it is hard to measure current and efficiency easily.

While we consider Sn^0 oxidation to Sn^{+4} as the main anodic reaction, on the counter electrode, we surmise the Hydrogen Evolution Reaction (HER) in hydroxide conditions to predominate: $2H_2O + 2e^- \rightarrow H_2 + 2OH^-$ (since Faradaic efficiency does not reach 100%, other side reaction are also possible). We noticed a slight pressure raise on the reactor's pressure gauge and the characteristic sound when opened. From our calculations, regarding the prediction of the amount of formed H_2 based on 100% Faradaic efficiency for Sn oxidation and parallel HER, we found out that less than 3 mmol of H_2 are formed, when the tin amount equaled the Si/Sn ratio of 50 is dissolved, causing a pressure increase up to 2.4 bars.

ST2.6. Energy efficiency calculation for metal-containing zeolites synthesis via EAS route

During the EAS of zeolites, consumed energy (in kW h) was measured by EAS RS Pro Energy Meter 178-5376. The meter was plugged between the power source of all reactor components: heating, control and generator. Measurements were completed for MFG and SRS systems at different conditions. Data was recorded after every 24 hours for the MFG system, and after 8 or 16 hours, in the case of the SRS generator. In addition, an average spent energy for heating was estimated by measuring power consumption of the whole system when the generator was shut down and deducted from a period during which EAS was in active use.

On average, a required power for heating at 90 °C measured during 72 hours experiments was 29.6 W. On the other hand, by calculation of generators consumption, MFG required 25.9 W (for only 1 channel working in the following mode: 1.0 V_{PP} signal in sine wave with 50 μHz frequency), and SRS was tested for three different voltages: 1.0, 2.0 and 4.0 V_{PP} resulting in 22.1, 22.0 and 21.4 W, respectfully, showing a very similar behavior on a wide range. Therefore, in a full 72 hours experiment, generators consume about 42.4 and 46.8 % of the total power of the EAS system if used for the full time period (which is not advised, see timing EAS, ST2.8). However, in the timed mode of EAS experiments, the most efficient mode for tin incorporation, SRS demonstrates the lowest amount of energy – 7.8 %.

ST2.7. Experimental design for ex-situ tin precursor preparation by anodic oxidation

To complete the experiment where metal precursors were introduced to a synthesis liquor by ex-situ anodic metal oxidation (i.e., releasing Sn prior to zeolite synthesis), the procedure of a zeolite synthesis in a classic batch reactor was divided into two parallel steps. TEOS hydrolysis and tin dissolution were done separately as the batch composition must remain unchanged. Half of the pre-diluted OSDA solution was used for each of the steps. Complete hydrolysis of TEOS and ethanol evaporation were finished within 3 days at room temperature. After that, the silicon solution was kept under stirring for 4 more days. In the second parallel beaker, a calculated amount of tin chloride pentahydrate was dissolved in the diluted OSDA solution for the final batch composition of Si/Sn 125.

Meanwhile, in the second beaker with half of the OSDA needed for the synthesis, the anodic oxidation of tin was performed at slow speed to obtain a tin solution with the same proportions: 18 hours with 1.5 V_{PP} with stirring. In these conditions, no metallic sediments were observed. As

the dissolved amount of tin was a bit higher than needed, a small amount of the prepared OSDA-H_2O mixture was added. Finally, the hydrolyzed TEOS (with other half of the OSDA) and the tin-OSDA solution were mixed together and kept for two hours under vigorous stirring. Then the formed liquid gel was transferred into a Teflon-lined stainless steel autoclave, which was placed in an oven at 90 °C for 72 hours. The zeolite washing procedure was conducted as usual (e.g., for MFI hydroxide synthesis).

As a result, synthesized Sn-MFI (both crystalline) materials had Si/Sn ratios of 540 and 370 for $SnCl_4$ and anodic Sn used as precursors, respectively (CD_3CN FT-IR – Figure S20).

In addition, another portion of TEOS was hydrolyzed directly with diluted OSDA solution with residues of tin from the EAS. Batch Si/Sn composition was 142. The rest of the conditions remained unchanged. The sample resulted in an MFI zeolite with the Si/Sn framework ration of 590, which makes it comparable with the sample synthesized with $SnCl_4$ salt due to the higher starting Si/Sn batch composition. In other words, the ex-situ EAS tin release did not show any advantages versus a classic synthesis in that case.

Possibly, such a great contrast in tin incorporation for in- and ex-situ results is achieved due to the generation of different species. Classic species from high pH hydrolysis of salts are of the $Sn(OH)_{4+p}{}^{p-}$ kind,[9] whereas according to the Pourbaix diagram at the measured pH of 12.3, EAS species should be hydrated $Sn(OH)_6{}^{2-}$ or $Sn(OH)_3{}^{-}$, depending on the voltage. Likely these species equilibrate fast if not incorporated swiftly into growing zeolite.

ST2.8. Experiment design for more efficient tin incorporation by the right timing of the metal release

Timed EAS procedures for efficient tin dissolution-incorporation ratio was run at 90 °C. In the EAS reactor, the first condition is immersed tin-made electrodes, but no electricity was applied for 16 hours from the beginning, to overcome kinetics of the Sn-MFI crystallization – the same time is required for pure siliceous MFI to pass the nucleation stage (16 h). Secondly, a difference of potentials of 2 and 4 V_{PP} was switched on for 8 hours. After that, the system remained in electricity-free conditions to crystallize for the rest of the zeolite formation time, i.e., 48 hours. Finally, thus after 72 hours of synthesis time, the product was washed and dried as described, e.g., for MFI hydroxide synthesis.

Because timed experiments have shown higher incorporation efficiency for Sn-MFI zeolite synthesis, other frameworks were synthesized using the timing approach. Establishing the very time-consuming experimental crystallization profile (as done for the Si-MFI system) was not feasible for CHA, the metal release was performed along their synthesis time by estimation based on the crystalline product outcome of a pure siliceous system.

The design of timed experiments was as follows (visualized below):

- Sn- and Zn-MFI zeolites: 8 hours of applied voltage (2, 4, or 5 V_{PP}; 50 µHz; sine wave) timed after the first 16 hours of the synthesis procedure
- Al-MFI: 24 hours of applied voltage (5 V_{PP}; 50 µHz; sine wave) timed on the second day (24 hours) of 72 hours synthesis procedure
- Sn- and Ti-CHA zeolites: 2 hours of applied voltage (2 or 4 V_{PP}; 25 µHz; square wave) timed at the beginning of the first or the day of 72 hours synthesis procedure

Scheme of timed experiments:

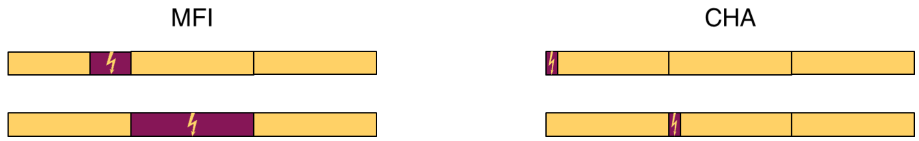

ST2.9. EAS of Zn-MFI and its characterization

Zn-incorporated zeolites were successfully synthesized via the EAS route. Besides the classic mode of EAS at 90 °C, syntheses at 160 °C was performed. Additionally, zinc release was conducted in the timed mode as was described in ST 3.8: For the zinc anodic oxidation experiment, an electric sine wave with an amplitude of 5 V_{PP} (50 µHz) was applied for 8 hours after the first 16 hours of the experiment, the total crystallization time was 72 hours. At last, a mixed tin-zinc material was synthesized using two different electrodes in the classic EAS mode with 1 V_{PP}, 50 µHz, sine wave over 72 hours, and the oxidation was started from the Sn-made electrode (anode at the start). For all conducted experiments, the total immersed electrode area was 13.4 cm^2.

Additionally, a series of zinc-impregnated samples was prepared. Zinc acetate solutions with various concentrations were deposited on three different Si-MFI by incipient wetness impregnation. Samples were kept closed with an hour glass at room temperature for 3 hours, then dried overnight at 80 °C. Dried products were calcined with the following program: a slow temperature increase until 550 °C for 9 hours and a hold for 6 hours at 550 °C (Samples characterization – Figure S15; Table S10).

The following methods were used for the proof of zinc incorporation in a zeolite lattice: ion exchange capacity towards mono- (K^+) and divalent (Ni^{2+}) cations; a bulk ICP-AES analysis for determination of leached zinc from zeolite; TEM EDX; and low-temperature N_2 physisorption.

It was already shown that zincosilicates with CHA structure possess remarkably higher ion-exchange ability for divalent cations, e.g., Ni^{2+}. The ion-exchange was studied using 0.1 M KCl and 0.05 M $Ni(NO_3)_2$ solutions. For a general test, 50 mg of a calcined sample was loaded in a centrifuge tube and filled with 50 ml of solution and a magnetic stirring bar. Afterward, a tube was left for stirring at 600 rpm for 96 hours at room temperature. Then, a sample was centrifuged for 5 minutes at 6200 rpm, washed once with Milli-Q water, and dried overnight at 60 °C.[1]

Similar experiments were conducted for a series of ZnO/Si-MFI and Zn-MFI zeolites; the results are summarized in Table S5. First, impregnated samples possess an insignificant ion-exchange capacity, while a higher number for K^+ exchanges could give information about siliceous defects of a structure. Second, comparing batch samples synthesized at different temperatures of 90 vs. 160 °C demonstrate that ion-exchange capacity decreases drastically: Ni/Zn 0.73 vs. 0.03, respectively. In other words, it suggests Zn-incorporated material can be achieved better using low-temperature conditions in the batch system. Third, all zincosilicates synthesized via EAS indicate the presence of framework zinc cites due to a high ion-exchange capacity. An EAS-assisted synthesis of Zn-MFI in the high-temperature mode also demonstrates partial incorporation of the metal even. The mixed metal Sn,Zn-MFI sample was additionally compared with an ion-exchanged Sn-MFI sample, which has the total Sn content as the sum of Sn+Zn. After a minor data correction, including subtraction of Sn sites ion-exchange capacity, the resulting Ni/Zn ratio of 0.57 fits the obtained trend.

Zinc is known for leaching from a zeolite framework, and the ion-exchange procedure helps distinguish this indirect parameter. The loss of Zn atoms per MFI zeolite cage after ion exchange

was calculated (Table S5) based on the HF digested ICP-AES data of Si/Zn ratio before ion exchange, and an average value between two Si/Zn ratios after K^+ and Ni^{2+} ion-exchange. Impregnated samples show less than 10 % of zinc loss. Likewise, the high-temperature batch synthesis value has the lowest percentage of zinc loss from all hydrothermal synthesized materials, presumably because of a high formation of non-framework zinc species (not sensitive to easy leaching). At the same time, the highest leaching behavior was demonstrated for Zn-incorporated samples obtained in low-temperature batch conditions or with EAS – 39 and 37 %, providing indirect proof of Zn incorporation.

As was already mentioned in the main text, the key advantage of EAS vs. batch synthesis is a low metal concentration profile during crystallization. This and a constant supply allow the metal to remain in a favorable state for zeolite incorporation. The difference was unveiled by TEM EDX for both samples synthesized at 90 and 160 °C. In contrast with the EAS sample, a closer look at nano-crystals after batch synthesis at 90 °C demonstrates the presence of a second zinc-rich impure phase, whereas the EAS crystals have a single phase with equal metals distribution (Figure 2E). In the high-temperature synthesis case, the batch sample shows uneven zinc content between micron-sized crystals and an occurrence of extraneous phase (Figure S24B). However, a similar picture is seen for the EAS synthesis: due to the rapid crystal growth at 160 °C, a slow R_r combined with fast zeolite crystallization results in metal-deficient Zn-MFI. We hypothesize that the silicon source was already spent, and that the onward excess of released zinc could be spent exclusively for an oxide phase formation, which agglomerates on crystal edges (Figure S25B). The formation of the layered phase was confirmed by PXRD (Reflex at 5.8 2θ degrees, Figure S8).

In the end, for the creation of a zeolite with well-dispersed framework zinc, EAS could really help once the kinetic of the growing siliceous phase is mapped and a low, controlled zinc concentration along the synthesis path (EAS zinc release) is applied. In such EAS conditions, with low concentrations of Zn, the effects of media on excessive zinc species and Zn oxide formation should be minimized. The case for Zn opposed to Sn is not directed toward the same objective: while for Sn, as much sites as possible seems desired (avoiding SnO_x) and this is not possible in current syntheses without EAS. For Zn, routes to high loaded Zn-silicates exist, but the challenge lies in controlling the ratio of framework Zn vs external Zn-oxide, at intermediate (more stable) Zn loadings.

ST2.10. EAS of Al-MFI

Al-containing MFI zeolite was synthesized with anodic oxidation of pure Al as electrode material. A couple of aluminum-made electrodes (13.4 cm^2) was attached to internal screw-type connectors, and the metal release was realized in the timed mode (ST2.8): 24 hours of 5 V_{PP} voltage difference with 50 μHz frequency were applied exclusively for 24 hours after the crystallization took place for the first 24 hours (24-24hE-24).

ST2.11. EAS of Fe-MFI

A series of EAS experiments were performed for the anodic oxidation of iron. It was found that its behavior is quite similar to aluminum in hydroxide media.[2] Moreover, the electrode surface is extremely passive and must be partially activated in a weak acid solution prior to a reaction. For that, in a particular case of iron electrodes, before the acetone washing stage, they were exposed to a series of 30 minutes acid washings in 10 wt. % solutions consist of consequent HCl – HNO_3 – HCl treatment. At the end of the EAS reaction, a dark layer of iron oxide was formed on an entire electrode surface only after such initial pretreatment (Figure S34). Remarkably, the comparison

sample of the immersed electrode without applied voltage difference did not show any signs of iron presence neither in the liquid phase after reaction nor in the resulting zeolite sample. Contrary, the EAS sample in most cases (more than 30 trials) shows the presence of iron ions in mother liquor after synthesis, but the incorporation of the metal in the zeolites is much less firm in the used conditions. The formation of iron oxide seems more likely, since an ICP-AES analysis of a solid product shows the presence of iron, but UV-vis-NIR spectroscopy does not reveal characteristic bands for the incorporated metal.

Successful synthesis of Fe-MFI zeolites in a classic batch reactor is quite straightforward and was performed for various temperatures, concentrations, and durations (Figure S10), the Si/Fe ratio of resulting zeotypes usually equals its batch loading. The EAS of iron-containing MFI zeolite was eventually performed by a complex treatment: two temperature regimes combined with in-situ anodic oxidation. Firstly, the EAS with the amplitude of 10 V_{PP} was performed at 80 °C for 4 days, the low temperature to slow down iron oxide formation after its release into a hydroxide-rich solution. Secondly, the synthesis was stopped, and the reactor was opened; resulted products were stirred and equally divided into two parts: both phases of the first half were subjected to ICP-AES analysis (Measured Si/Fe of solid 75 and liquid 230) and to PXRD (Figure S10), and the second half was put in a Teflon-lined stainless steel autoclave for a further crystallization at 160 °C for 2 days. Thirdly, the final solid product of the secondary crystallization was analyzed with the ICP-AES (Measured Si/Fe 160). A UV-vis-NIR study performed on the latter as-made samples shows a vague noisy signal for the direct EAS sample and a firm number of iron-incorporated bands for the second sample (Figure S11). The synthesis of Fe-containing MFI is debatable in the first case, but more firm for the second example with a secondary classic crystallization following the EAS part.

Unfortunately, the main reason why Fe-MFI was formed from the Fe-rich solution after EAS is hard to establish because the secondary crystallization process could also cause it. Overall, for Fe, EAS is not yet successful. We suppose dissolved iron species might not be suitable for in-situ incorporation. Therefore, other synthesis procedures and conditions should be explored for making iron zeolites by EAS.

ST2.12. EAS of Sn-APO-5

Using the same synthesis mixture of EAS experiments, two more materials were reproduced in batch, as described in the literature,[3] for additional benchmarking. Two tin halide salts precursors with different valencies were tested, and both samples demonstrated tin uptake, resulting in Al/Sn ratios of 36 and 27 for Sn^{+4} and Sn^{+2}, precursors respectively, while only the $SnCl_2$ resembles the tetrahedrally coordinated tin band on the UV-vis-NIR spectrum (Figure S14). In fact, the tin (IV) chloride spectrum repeats the pure aluminophosphate behavior, suggesting that only Sn^{+2} salt led to successful Sn incorporation into the Sn-APO-5 structure.

ST3. Time a sine spends below a threshold value, and, 'average voltage' for EAS

As it was discussed in the main part of the paper, the threshold of anodic tin oxidation in MFI gels was found to be at 0.6 V_{PP}, one more aspect of EAS has to be considered using the example of anodic tin dissolution at the 2 V_{PP}. (Figure S1). Since both electrodes are made of tin, oxidation happens consequently from each of them, and the time of dissolution is controlled with the bias changing frequency. The standard form of the AC electric wave is a sinusoidal function. In, in one wavelength, it passes from zero to zero through the maximum and minimum of the function, changing its sign from plus to minus (Figure S1A).In other words, the actual time of each electrode dissolution should be half the period of a sine wavelength in the case of electrodes made from the same material. Using the same frequency, this is also applicable for the square waveform function (Figure S1B), whereas for a DC experiment, dissolution will happen only from one electrode (Figure S1C).

Another important observation for the EAS of Sn-MFI experiments in case of sine vs. square waveforms, was that despite a lower release rate, the sine wave promoted the formation (and exerted better control) of tin-rich material better than the square wave. It was concluded that a higher R_r is not a guarantee (Figure 1) of successful metal incorporation. Either an equilibrium between silicon pre-zeolite matrix and released metal cannot be accessed or the too high R_r brings the initial Sn concentration too high (as in the case of batch syntheses) and blocks or hampers some parts of the zeolite synthesis process incorporating Sn. the form of the square wave has the maximum value all over the reaction time, while the sine wave reaches its maximum (amplitude) only twice per period of the function. There, the actual potential is much lower for the majority of the time. Noticeably, since a metal dissolution threshold exists, this plays an important role for a sine wave as parts of the wave will not instill the anodic oxidation process (even for a V_{PP} well over the 0.6 threshold).

For one, we could estimate the proportion of the time a wave spends above the threshold value or one could, for a better comparison of sine and square waveforms, use an average sine function value for the applied potential. This could be more comparable to the nominal value of the square wave function. For the sine, the corrected average Voltage (V_{av}) can be easily calculated by the following formula:

$$V_{av} = \frac{1}{b-a}\int_{a}^{b} f(x)dx \text{ for the } f(x) = A \times sin(x) = \frac{V_{pp}}{2} \times sin(x) \text{ and } a = 0, b = \pi$$

$$V_{av} = \frac{V_{pp}}{\pi}$$

Figures

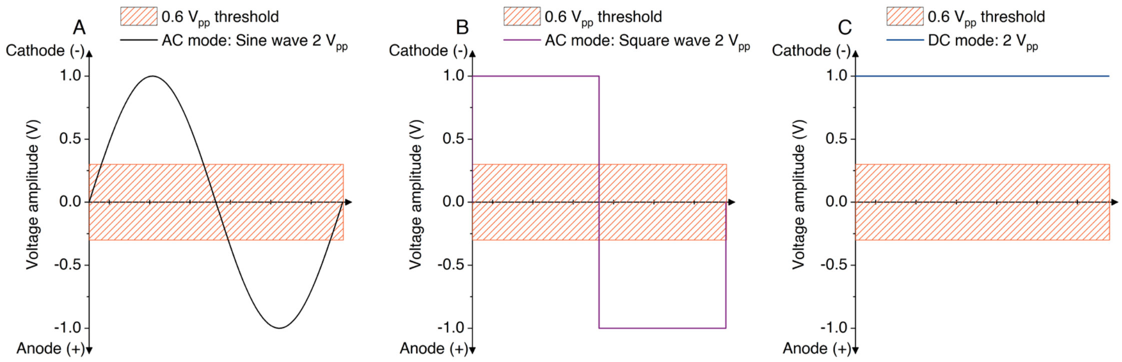

Figure S1. Graphical representation of different electric waves used for EAS.
AC mode in (**A**) sine or (**B**) square waveform, (**C**) theoretical DC mode at constant voltage vs. the experimentally established tin dissolution threshold.

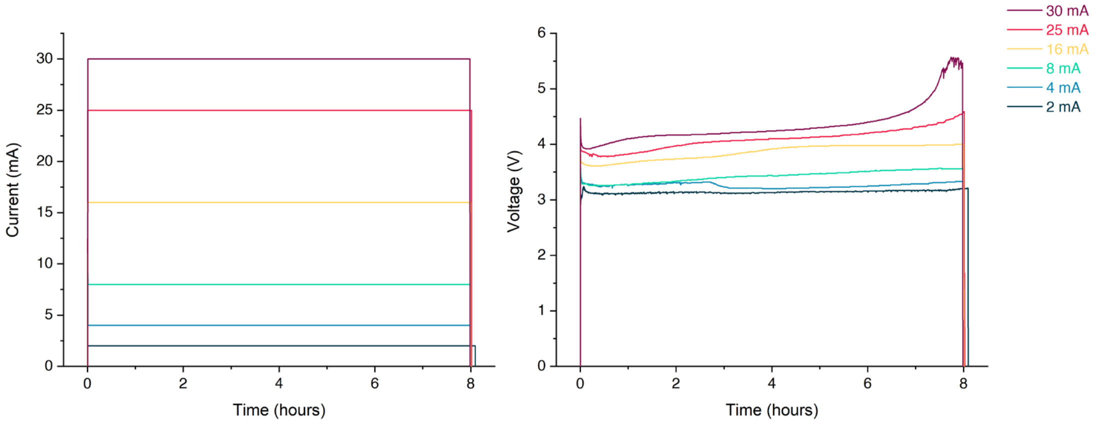

Figure S2. Recorded data of electric signal evolution in the constant current mode of a regular DC-assisted EAS experiment.

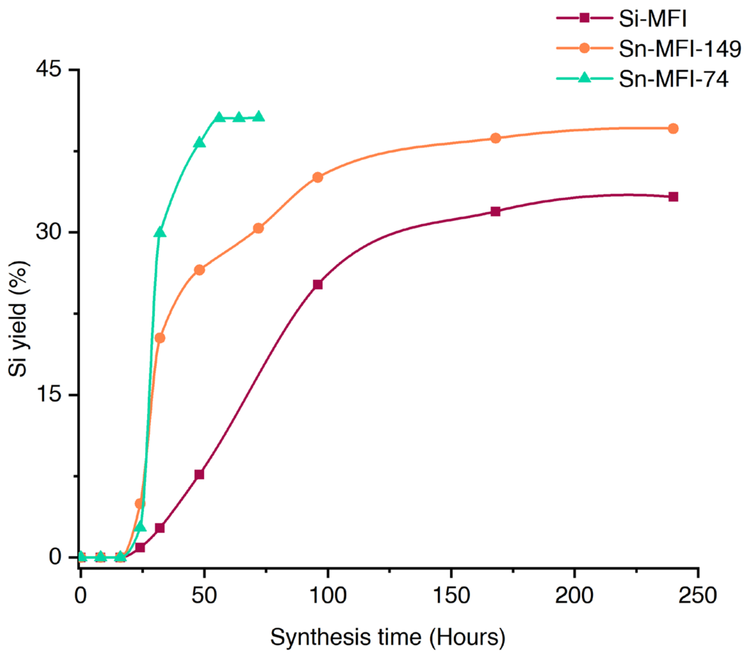

Figure S3. Comparison of crystallization kinetics of Si- and Sn-MFI zeolites in classic batch conditions at 90 °C. Si/Sn ratio of the mixture indicated (not the final product).

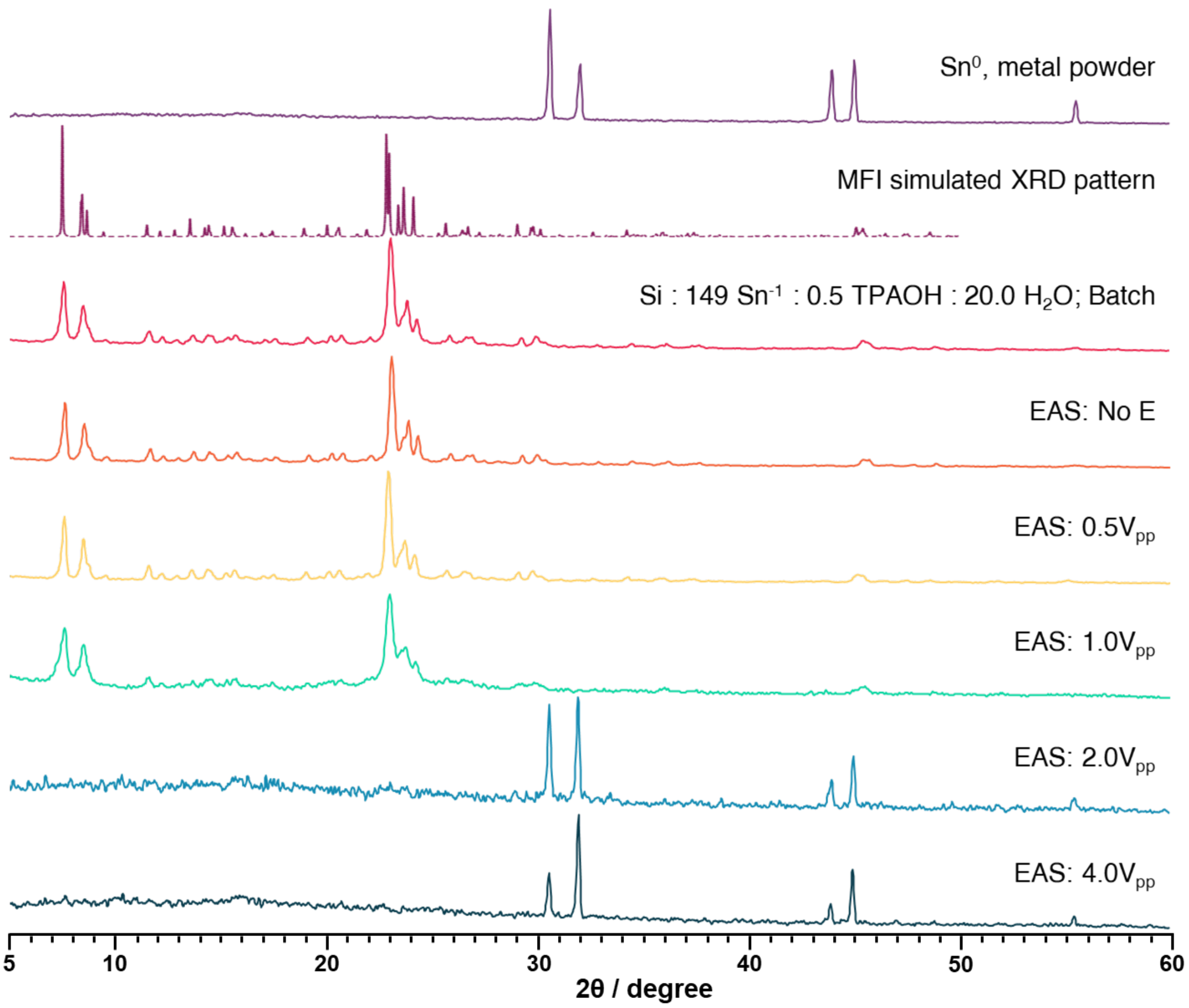

Figure S4. PXRD patterns of as-made Sn-MFI zeolites crystallized in batch and EAS conditions at 90 °C for 72 hours.

MFI EAS experiments had the same starting batch composition of Si:0.5TPAOH:20.0H_2O and used applied electricity of specified magnitude for 72 hours with sine waveform at 50 μHz frequency; immersed area of electrodes was set as 30.8 cm^2. No E means no voltage (potential) was applied.

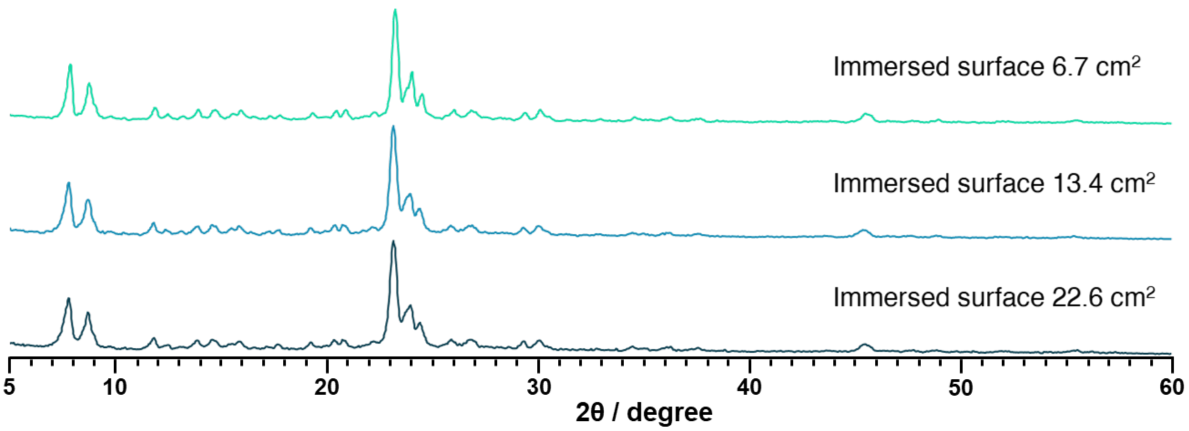

Figure S5. PXRD patterns of as-made Sn-MFI zeolites synthesized with EAS with a different electrode surface: 6.7, 13.4, and 22.6 cm^2.

The following dimensions were used to create electrode couples of different sizes: length – 4.0 cm, and width – 0.5, 1.0, or 1.3 cm, respectively. All experiments had the same starting batch composition of Si:0.5TPAOH:20.0H_2O. Syntheses were conducted at 90 °C for 72 hours with the following characteristics of applied electricity: 0.8 V_{PP}; 50 µHz; 72 hours; sine wave.

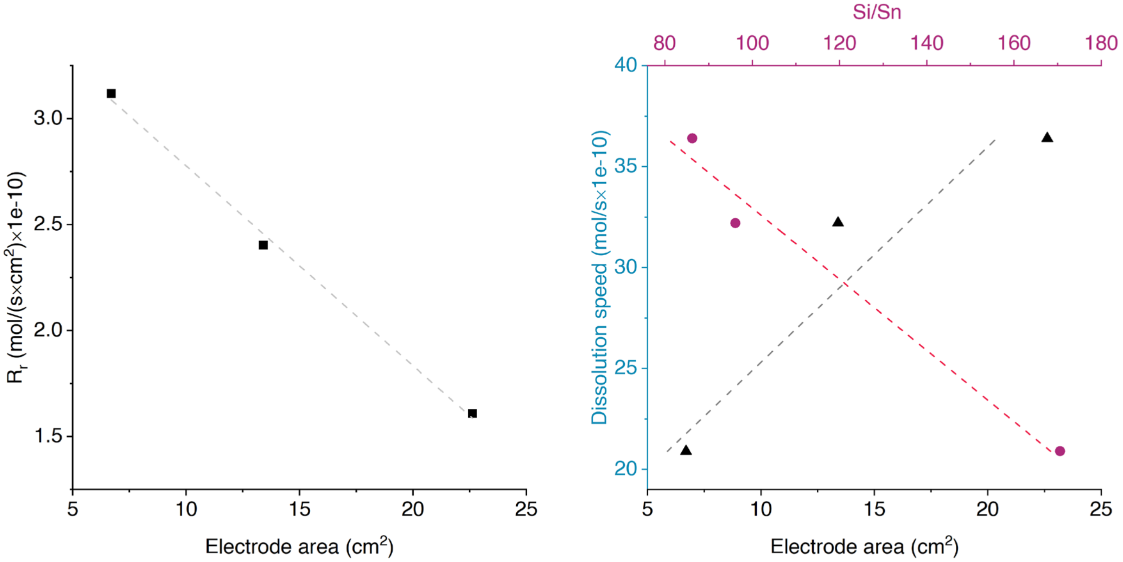

Figure S6. Correlation of tin anodic oxidation speeds (R_r on the left, dissolution speed on the right) with electrode area.

The linear correlation of tin release rate (**left**) and dissolution speed (**right**) from electrode area in correspondence with Faraday's law of electrolysis (ST2.4). Right: Dissolution speed versus electrode area (black triangles) and Dissolution speed versus Si/Sn obtained (purple dots).

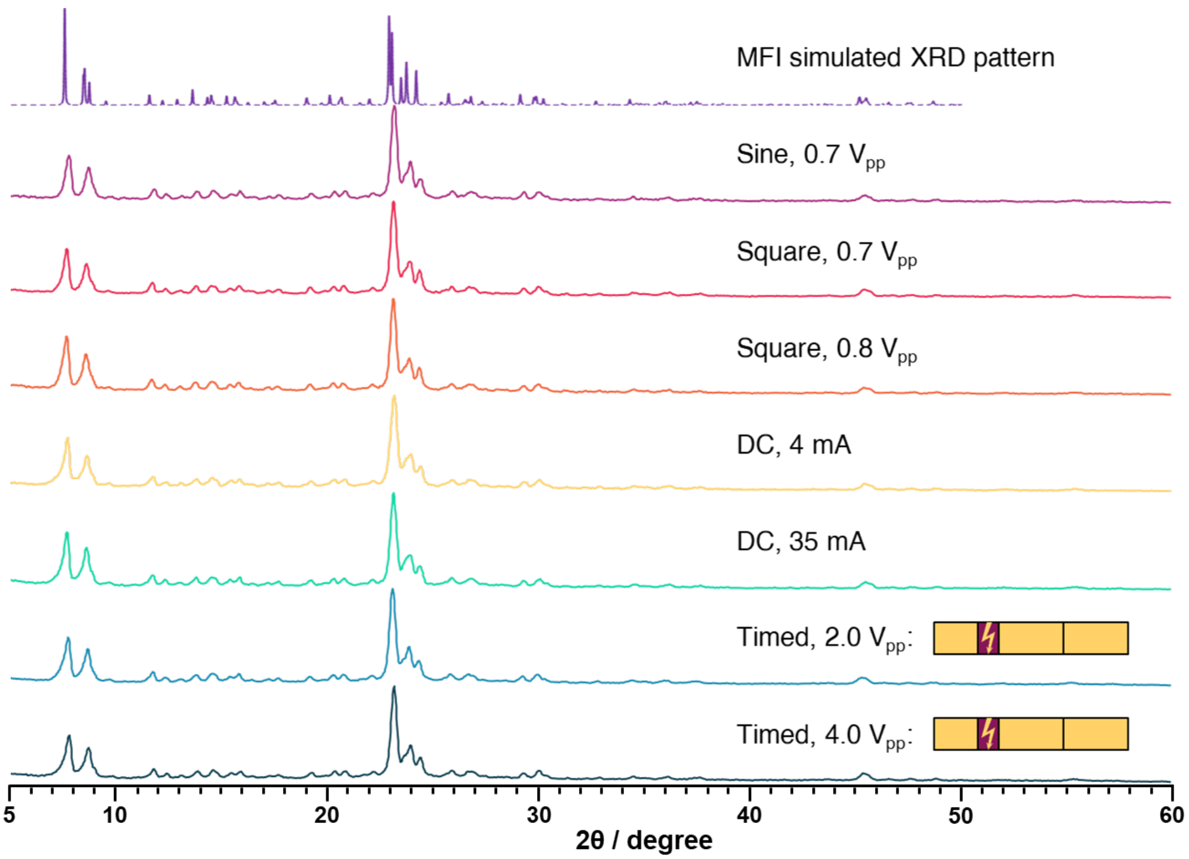

Figure S7. PXRD patterns of as-made Sn-MFI zeolites crystallized in EAS conditions at 90 °C for 72 hours.

All experiments had the same starting batch composition of Si:0.5TPAOH:20.0H_2O. Used applied electricity had the following characteristics: specified magnitude and waveform; 50 μHz frequency (except DC samples); 72 hours for sine and square wave, and 8 hours for DC and timed tin release (ST2.8); immersed area of electrodes equals 13.4 cm^2.

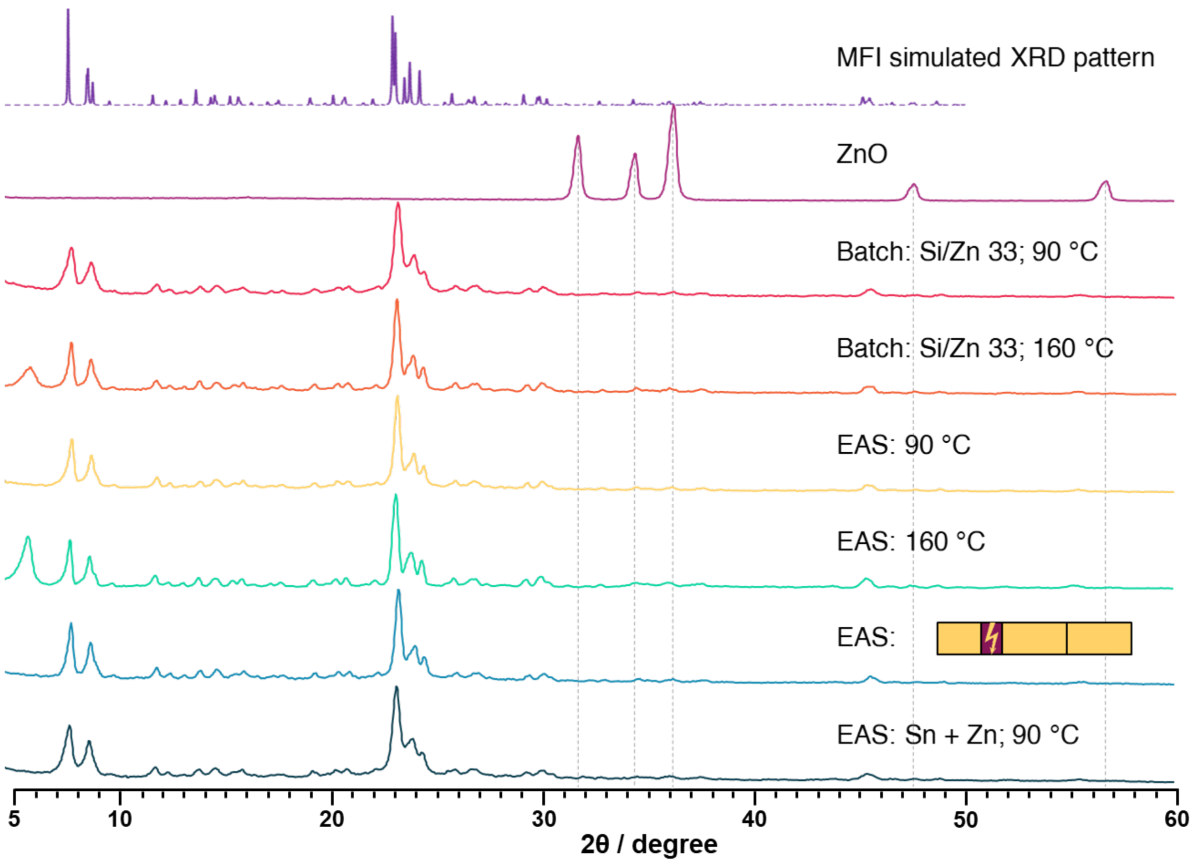

Figure S8. PXRD patterns of as-made Zn-MFI zeolites.

All samples were synthesized from siliceous starting gel with the batch composition of Si:0.5TPAOH:20.0H_2O for 72 hours. Both comparison batch samples had a starting Si/Zn ratio of 33. Classic EAS samples were run with the following characteristics: 0.8 V_{PP}; 50 µHz; 72 hours; sine wave; immersed area of electrodes equals 13.4 cm^2; the timed EAS sample was synthesized with the amplitude of 5 V_{PP} during 8 hours (ST2.8); the bottom sample with the simultaneous combination of tin and zinc electrodes was synthesized with the amplitude of 1 V_{PP}; sine wave (detailed description ST2.9). Zn-MFI zeolites synthesized at 160 °C have a reflex at 5.8 2θ degrees corresponding to the formation of layered material. This reflection disappears after calcination.

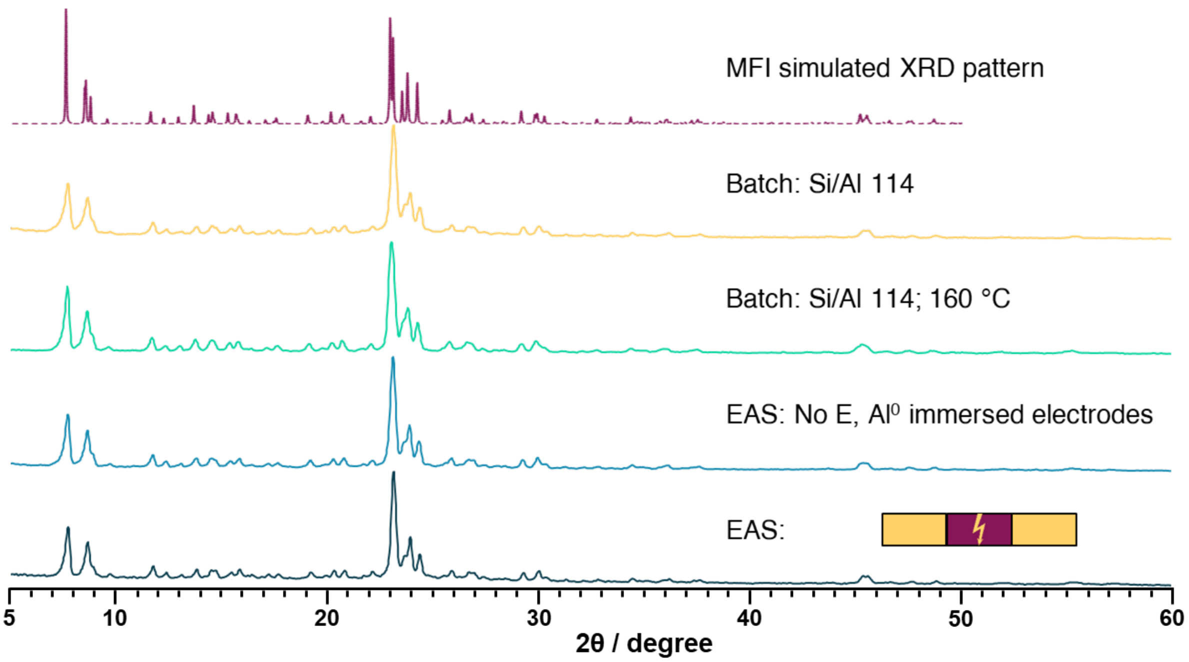

Figure S9. PXRD patterns of as-made Al-MFI zeolites.

All samples were synthesized from siliceous starting gel with the batch composition of Si:0.5TPAOH:20.0H_2O for 72 hours at 90 °C unless otherwise labeled. Both comparison batch samples had starting Si/Al ratio of 114. The EAS sample was run with the following characteristics: 5 V_{PP}; 50 µHz; 24 hours timed after the first 24 hours (72 hours of crystallization in total); sine wave; immersed area of electrodes equals 13.4 cm^2.

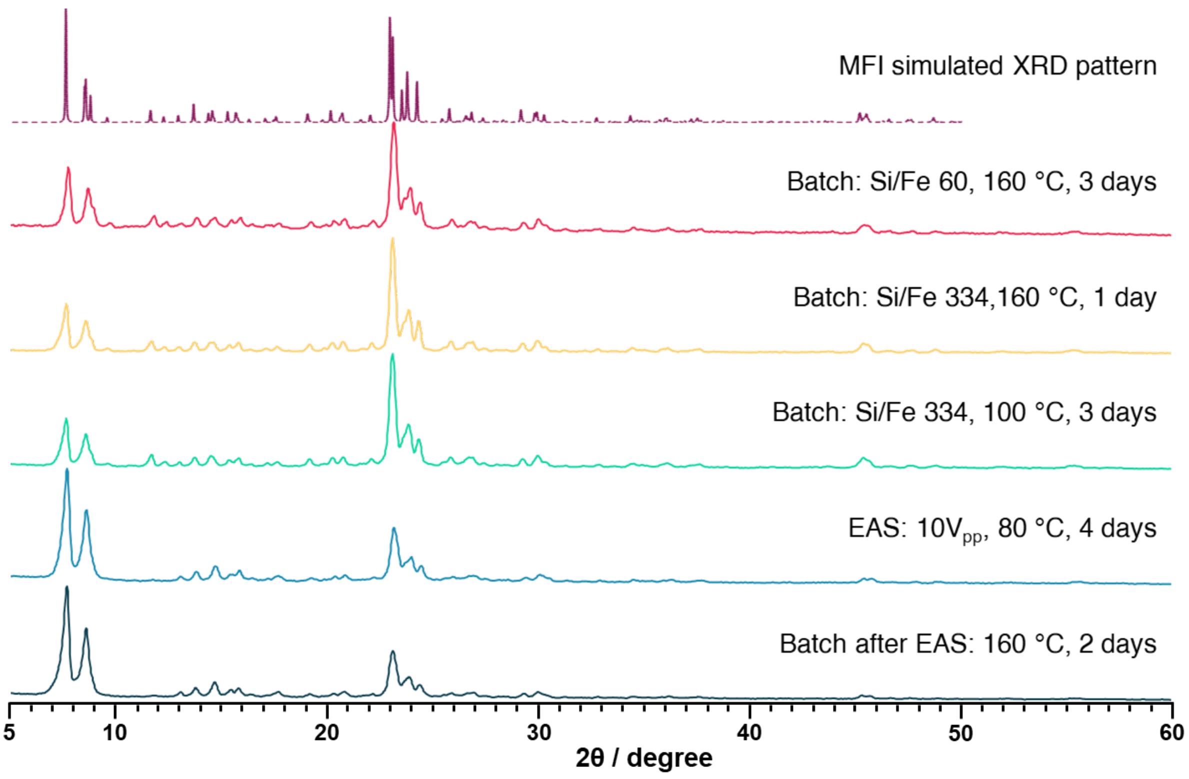

Figure S10. PXRD patterns of as-made Fe-MFI zeolites.

Samples were crystallized in batch and EAS conditions. All EAS experiments had the same batch composition of Si:0.5TPAOH:20.0H_2O However, a lower amount of OSDA was used for batch comparison samples: Si/TPAOH 0.2. A detailed description of the 'batch second crystallization' round after the EAS experiment is given in ST2.11.

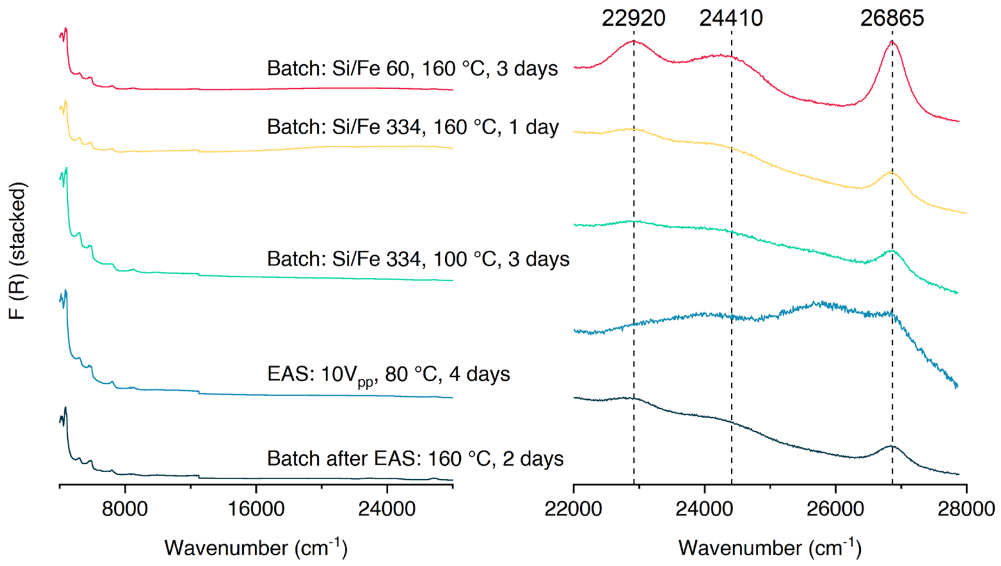

Figure S11. UV-vis-NIR spectra of as-made Fe-MFI zeolites.

Full spectrum (**left**), the specified region with three characteristic bands for zeolite-incorporated iron atoms (**right**). PXRD is demonstrated in Figure S10.

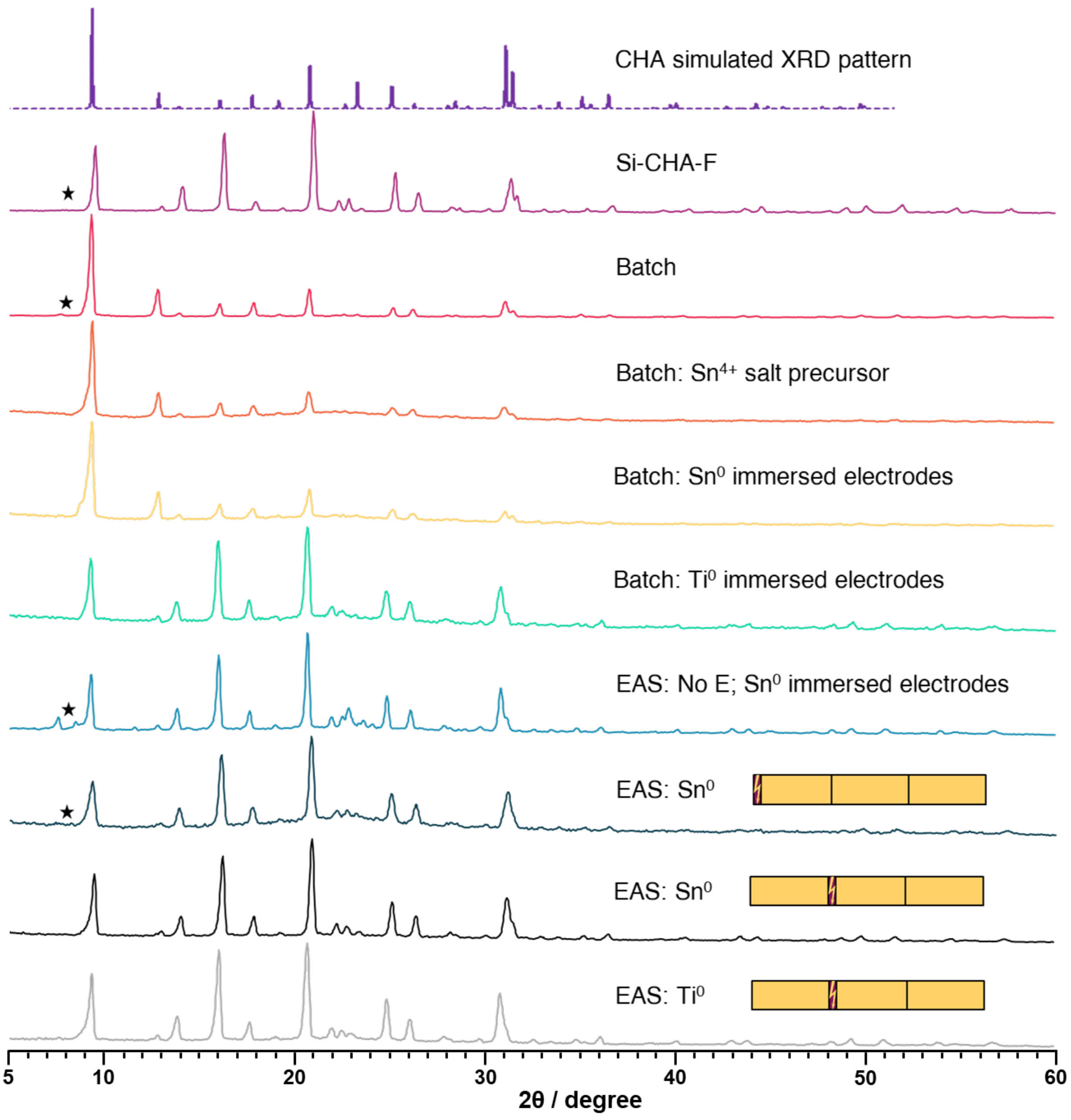

Figure S12. PXRD patterns of as-made CHA zeolites

All samples were synthesized from siliceous starting gel with the batch composition of Si:0.5TMAdamOH:4.0EDA:30.0H_2O at 120 °C for 72 hours. The Si-CHA-F sample shows the crystalline structure of siliceous seeds, which were synthesized separately. The spectrum of a sample made with Sn^{4+} precursor had the batch molar ratio of Si/Sn of 74. Three spectra with immersed electrodes demonstrate benchmark experiments in which electrode material was thermochemically dissolved due to synthesis conditions. Three latter samples were synthesized via the EAS route with applied electricity in the starting point of the first or the second 24 hours of a 72 hours experiment with the following characteristics: 2 V_{PP}; 25 µHz; 2 hours; square wave; immersed area of electrodes equals 13.4 cm^2. Patterns marked with the star symbol at 7.8 2θ degrees were found to have a trace amount of STT zeolite phase after the calcination procedure.

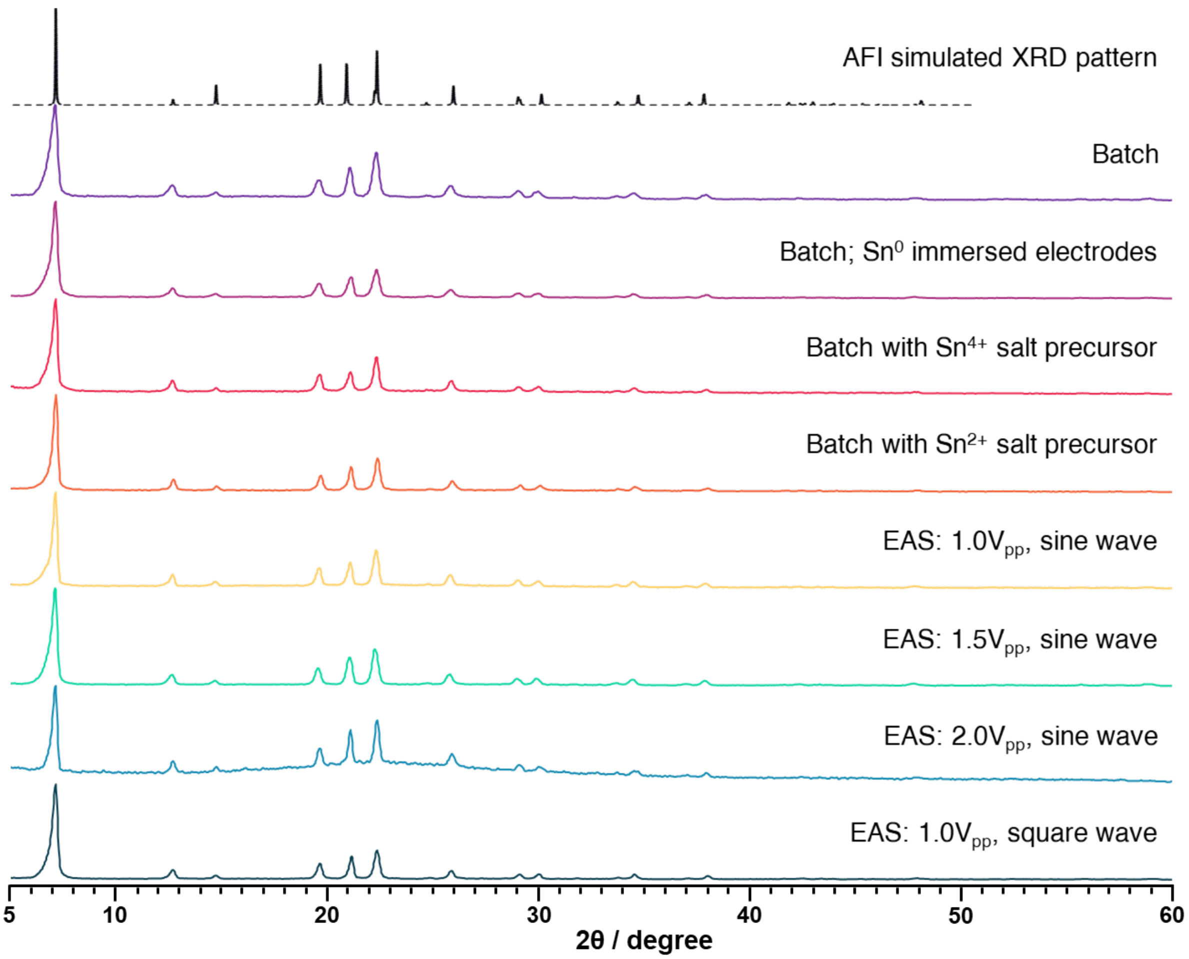

Figure S13. PXRD patterns of (Sn-)APO-5 after calcination.

Syntheses were performed from a gel with the batch composition of Al:(1-X)P:(XSn):0.8OSDA:50.0H_2O at 160 °C for 24 hours. The value of X was equaled to 0.04 for both syntheses using tin salt. Synthesizes conducted via the EAS route had applied electricity with the following characteristics: 50 μHz; 24 hours; immersed area of electrodes equals 13.4 cm^2.

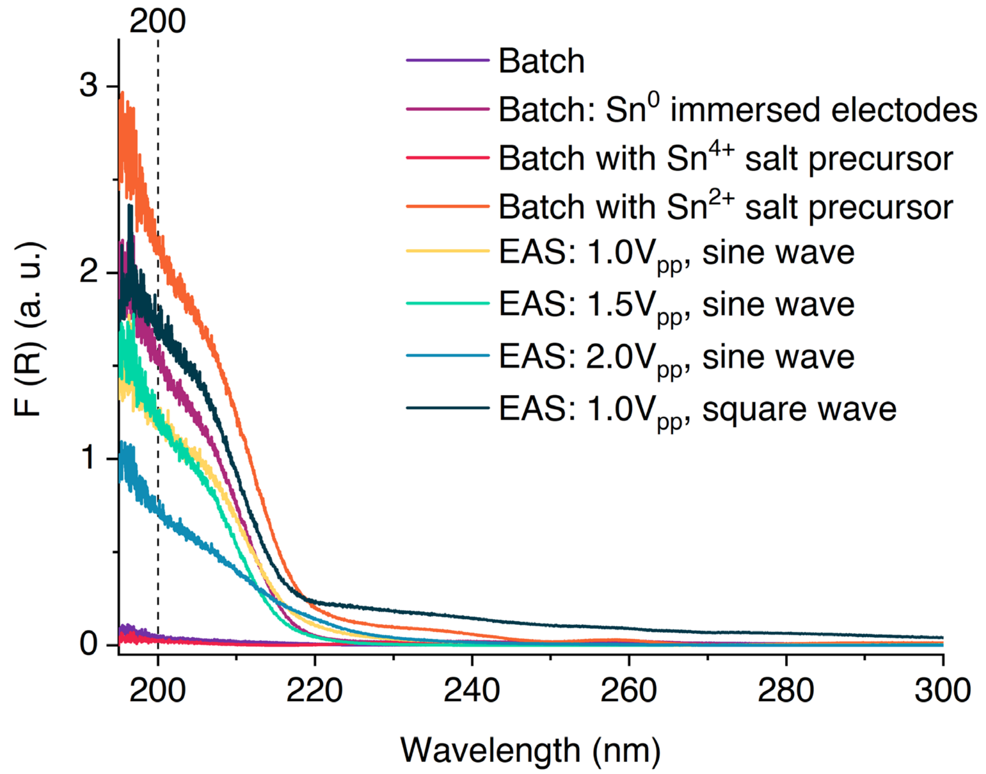

Figure S14. UV-vis-NIR spectra of as-made (Sn-)APO-5 materials.

Syntheses were performed from a gel with the batch composition of Al:(1-X)P:(XSn):0.8OSDA:50.0H_2O at 160 °C for 24 hours. The value of X was equaled to 0.04 for both syntheses using tin salt. Synthesises conducted via the EAS route had applied electricity with the following characteristics: 50 µHz; 24 hours; immersed area of electrodes equals 13.4 cm^2.

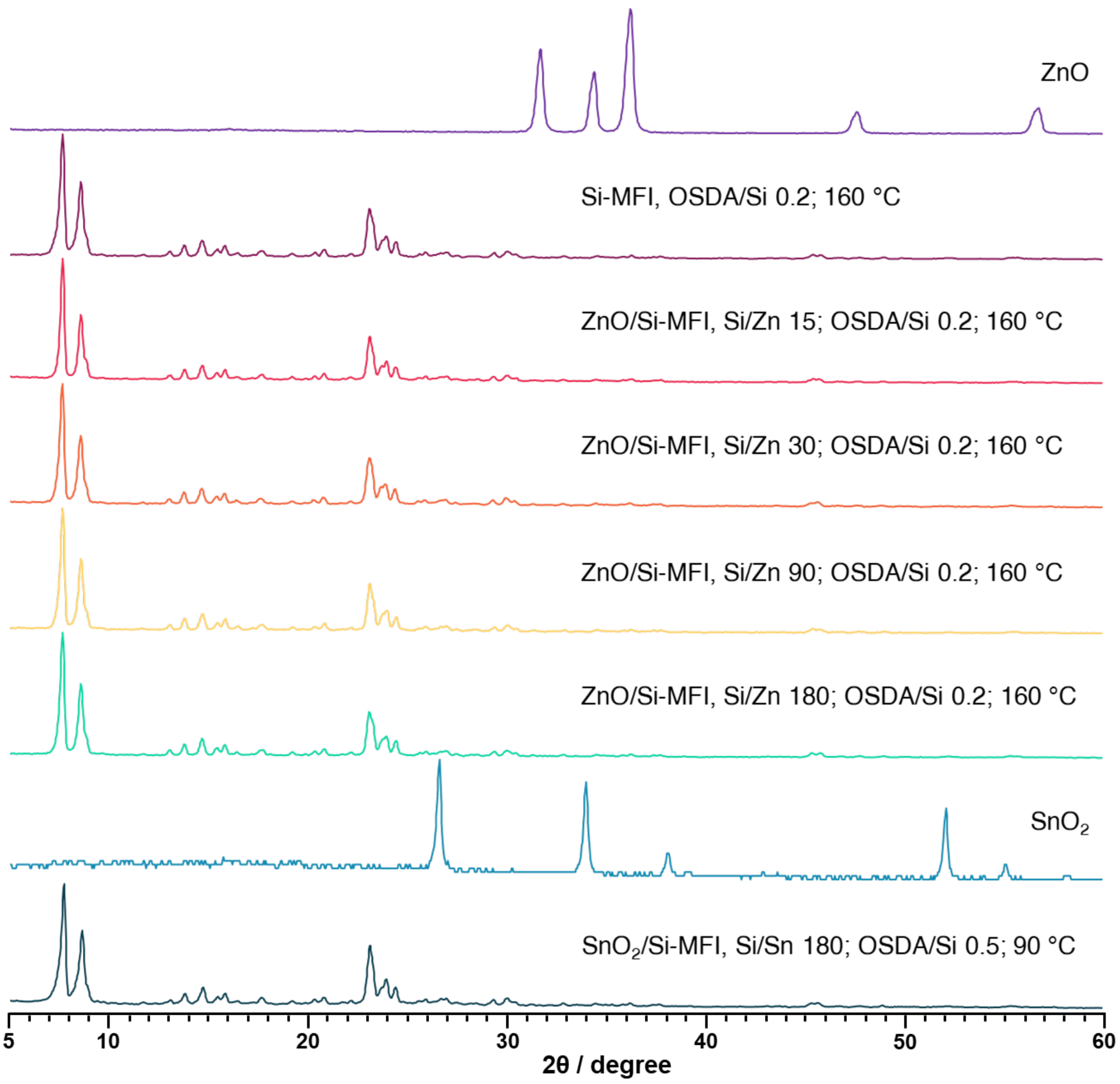

Figure S15. PXRD patterns of Si-MFI zeolites impregnated with Zn^{2+} or Sn^{4+} salts after calcination.

Siliceous supports were synthesized with a specified batch composition of Si:0.2-0.5TPAOH:20.0H_2O at 90 or 160 °C for 72 hours. No oxide phase formation was denoted for impregnated samples. Chemical composition and N_2 physisorption analysis data is given in Table S10.

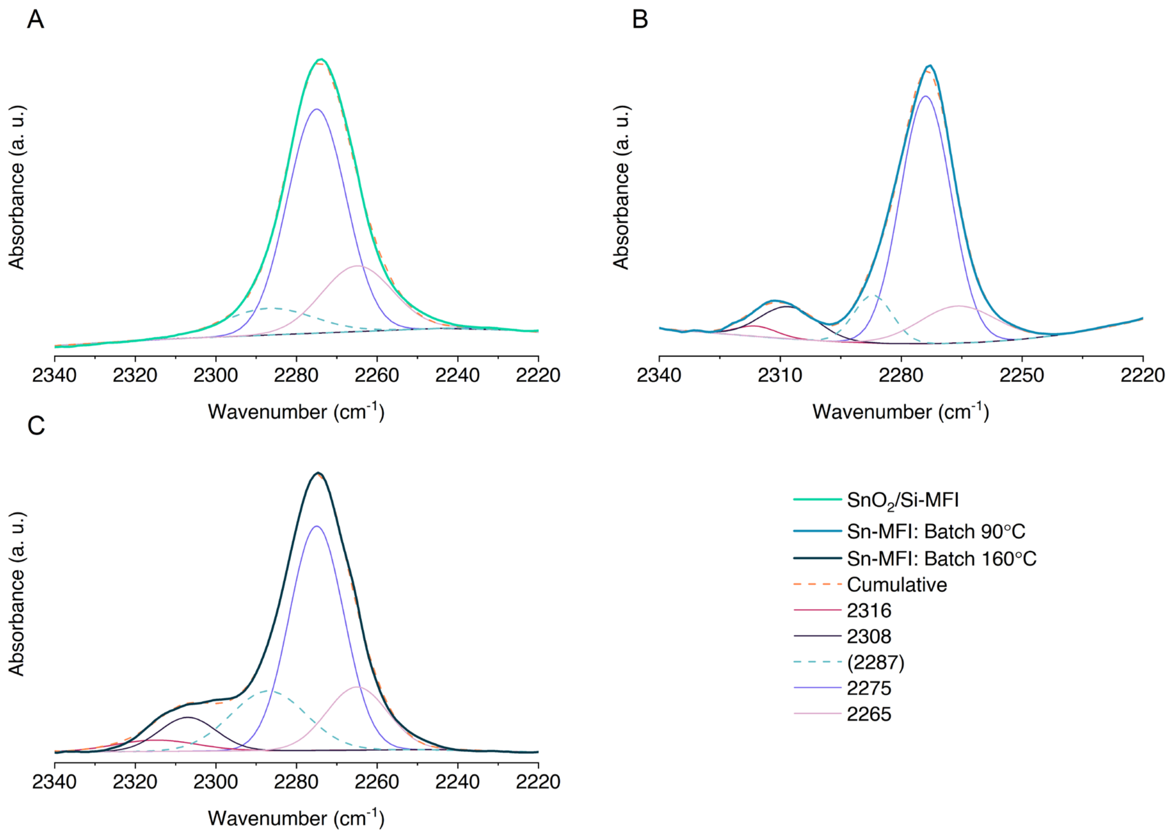

Figure S16. FT-IR spectra of adsorbed CD_3CN on tin-containing materials and deconvolution of their characteristic bands.

(**A**) SnO_2/Si-MFI, which was made by impregnation of Si-MFI zeolite (OSDA/Si 0.5) with $SnCl_4$ solution and later calcination (as was done for ZnO/Si-MFI – ST2.9); (**B**) classic batch Sn-MFI synthesized with the starting Si/Sn ratio 149 at 90 °C for 3 days; and (**C**) batch Sn-MFI was synthesized with the starting Si/Sn ratio 37 at 160 °C for 10 days.

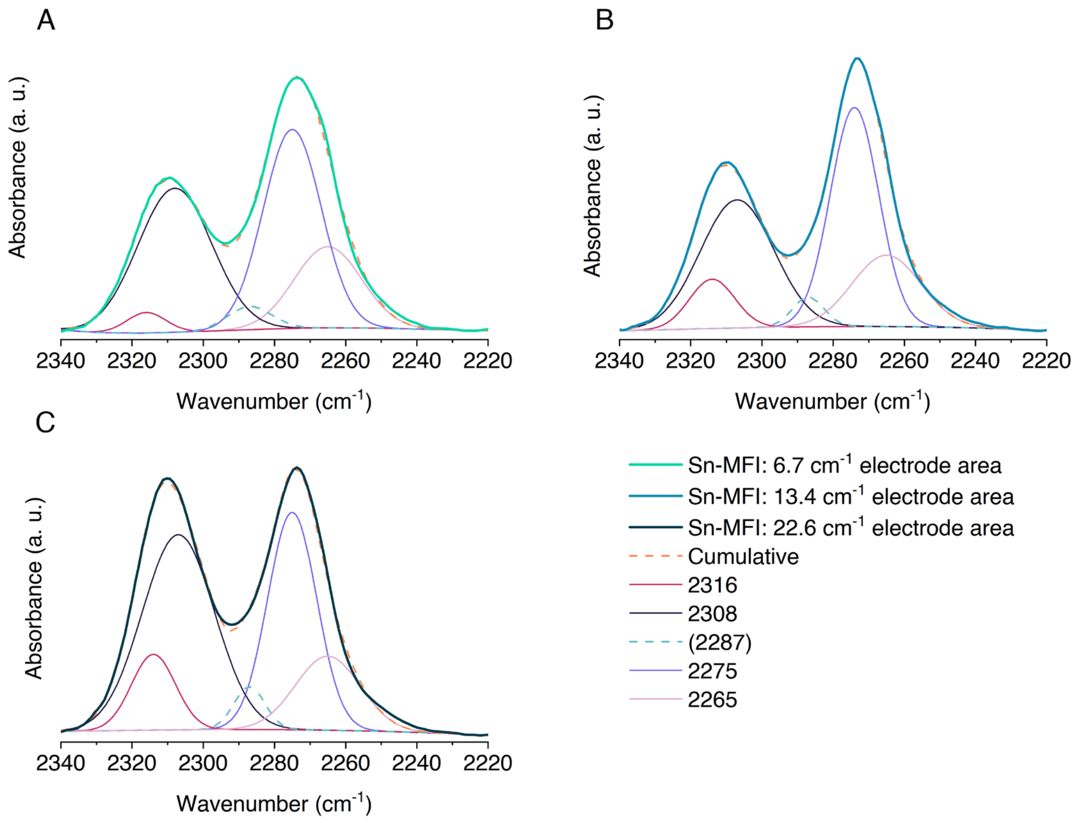

Figure S17. FT-IR spectra of adsorbed CD_3CN on EAS Sn-MFI with different electrode areas and deconvolution of their characteristic bands.

EAS Sn-MFI was synthesized at 90 °C for 3 days with different electrode areas: (**A**) 6.7, (**B**) 13.4, and (**C**) 22.6 cm^2. EAS parameters: 0.8 V_{PP}, 72 hours.

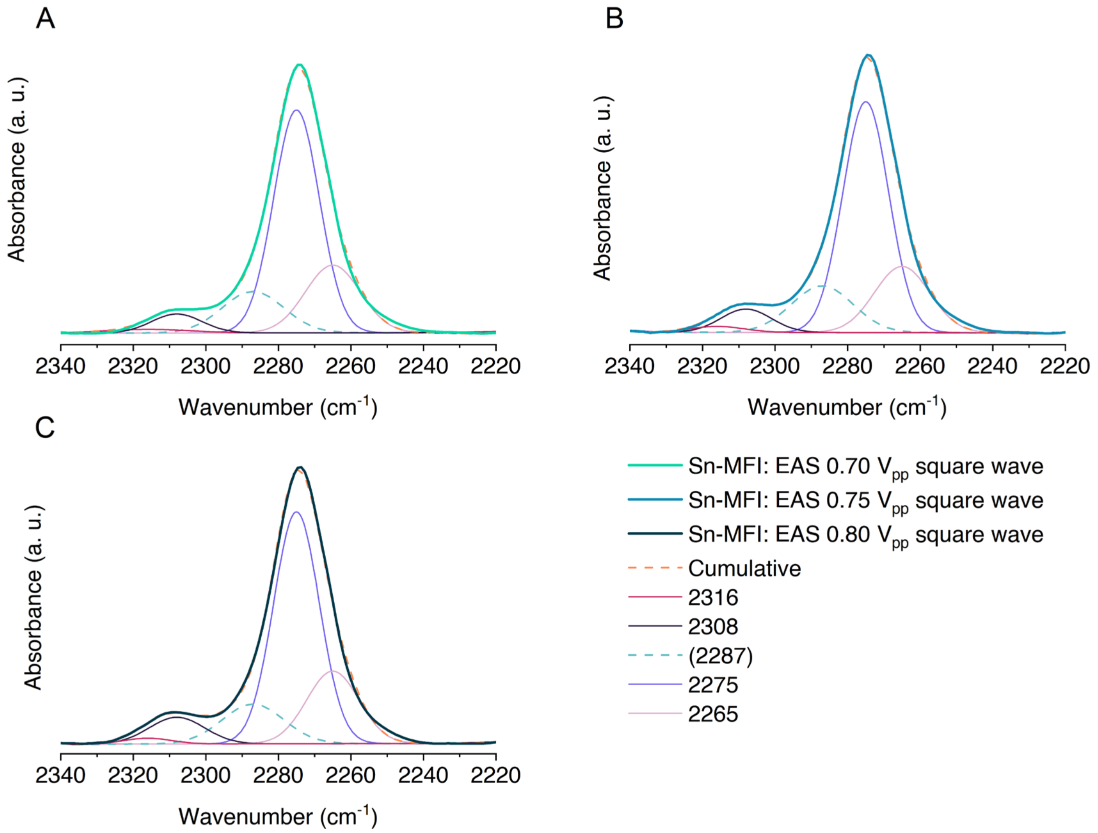

Figure S18. FT-IR spectra of adsorbed CD_3CN on EAS Sn-MFI zeolites using square waveform signal and deconvolution of their characteristic bands.

EAS Sn-MFI zeolites were synthesized using a square waveform signal with an amplitude of (**A**) 0.70, (**B**) 0.75, and (**C**) 0.80 V_{PP} for 3 days.

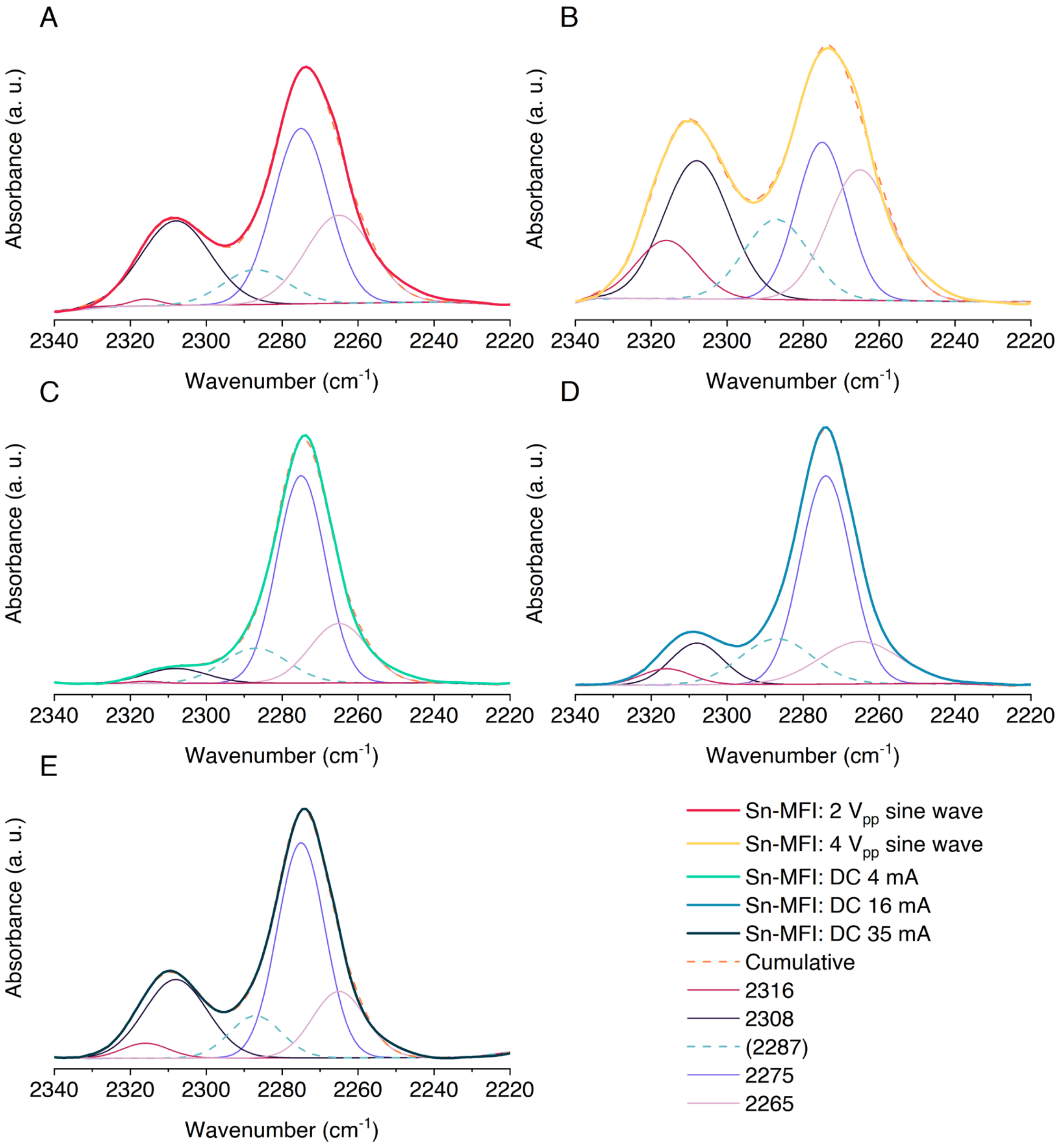

Figure S19. FT-IR spectra of adsorbed CD_3CN on EAS Sn-MFI zeolites synthesized in the timed mode and constant current experiments and deconvolution of their characteristic bands.

All syntheses were run for 16 hours, then electricity was applied for 8 hours, after which crystallization was completed with a total synthesis time of 72 hours (ST2.8). EAS was conducted at (**A**) 2 and (**B**) 4 V_{PP} amplitude of sine wave generated by SRS, as well as at (**C**) 4, (**D**) 16, and (**E**) 35 mA amplitude of constant current for the experiments in the DC mode.

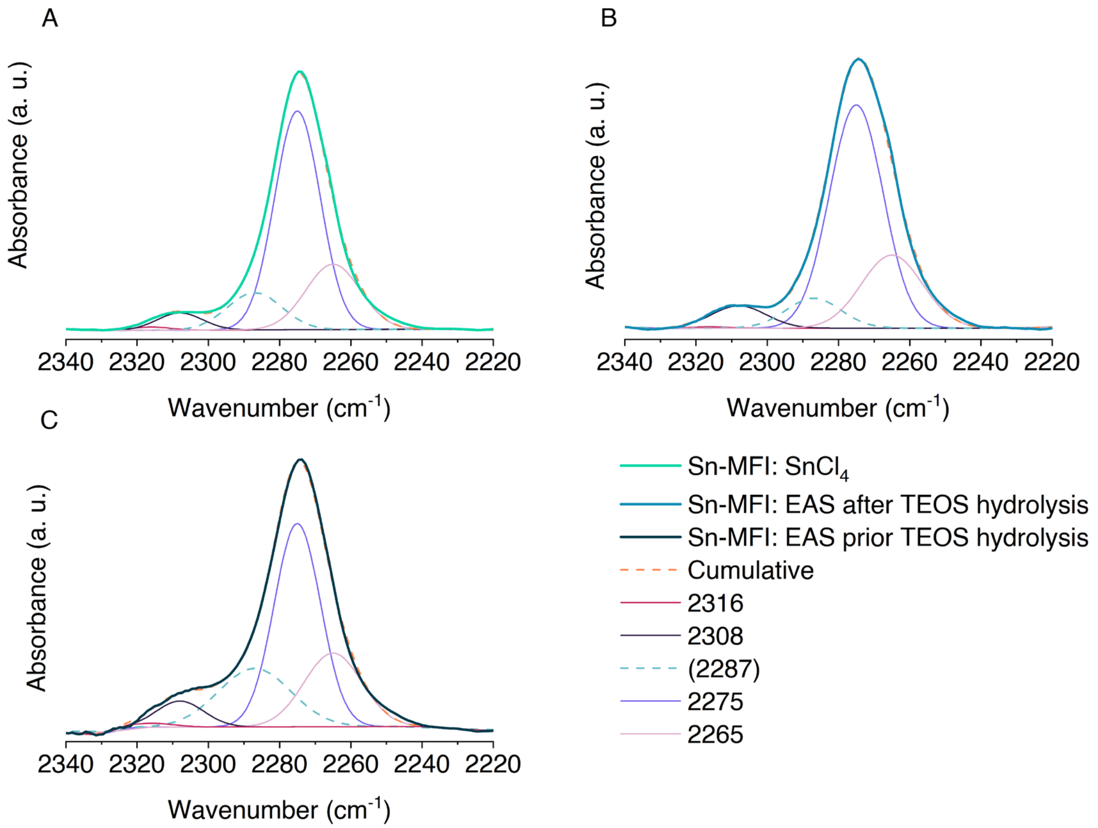

Figure S20. FT-IR spectra of adsorbed CD_3CN on Sn-MFI zeolites synthesized with different tin precursors (SI S3.7) and deconvolution of their characteristic bands.

Sn-MFI zeolites were synthesized with different tin precursors in classic batch synthesis conditions (SI S3.7): (**A**) tin chloride, (**B**) pre-released anodic tin release, and (**C**) anodic tin release prior to TEOS hydrolysis.

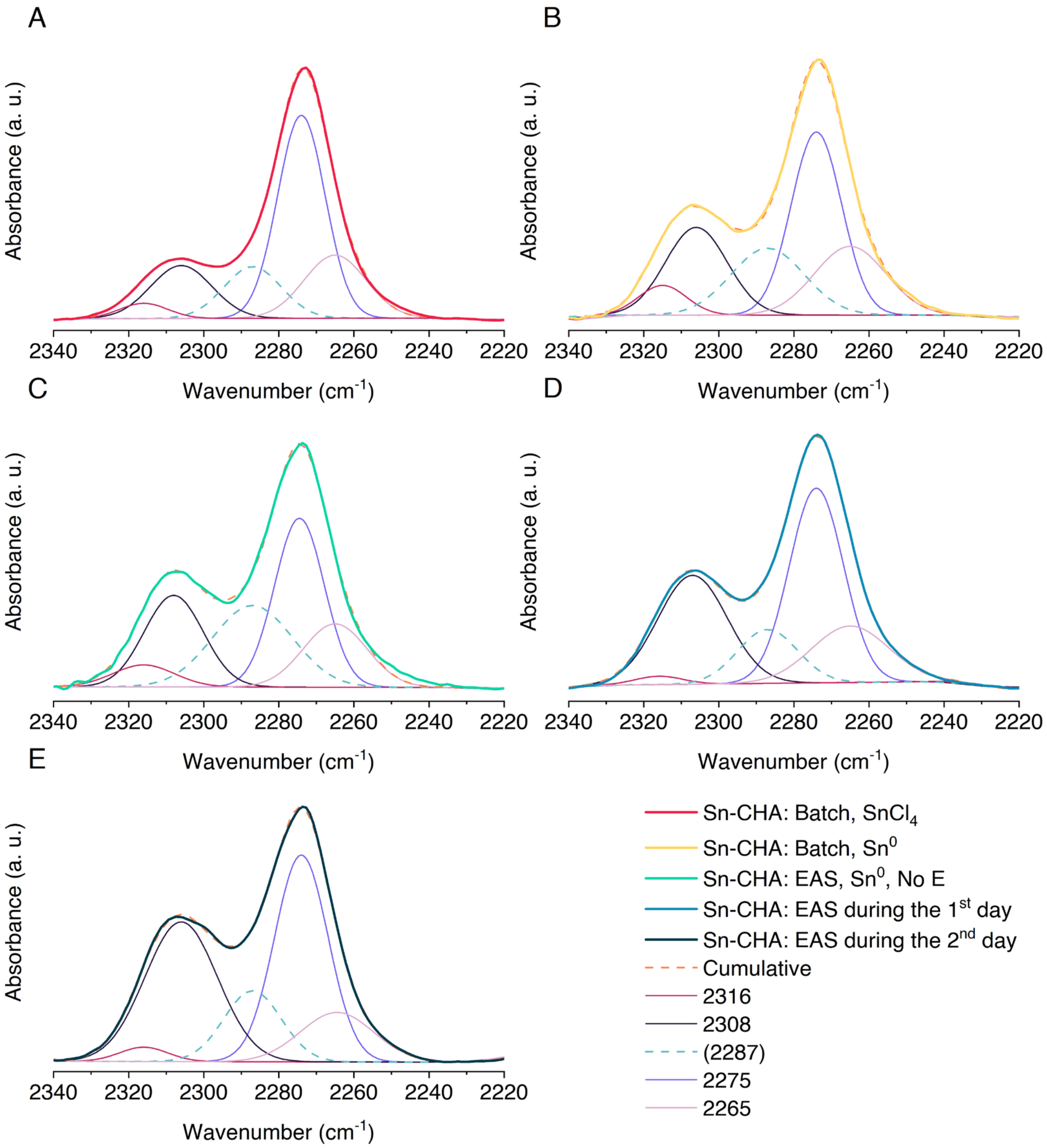

Figure S21. FT-IR spectra of adsorbed CD_3CN on Sn-CHA zeolites and deconvolution of their characteristic bands.

(**A**) Sn-CHA synthesized in the classic batch conditions with the starting Si/Sn ratio of 74 using $SnCl_4$ as the tin source; **(B)** blank sample synthesized in batch and (**C**) blank sample synthesized in electroreactor conditions using immersed Sn^0 electrode in the pure siliceous media (ST2.2). (**D+E**) EAS route samples with applied electricity in the starting point of (**D**) the first or (**E**) the second 24 hours of a 72 hours experiment with the following characteristics: 2 V_{PP}; 25 µHz; 2 hours; square wave; immersed area of electrodes equals 13.4 cm^2.

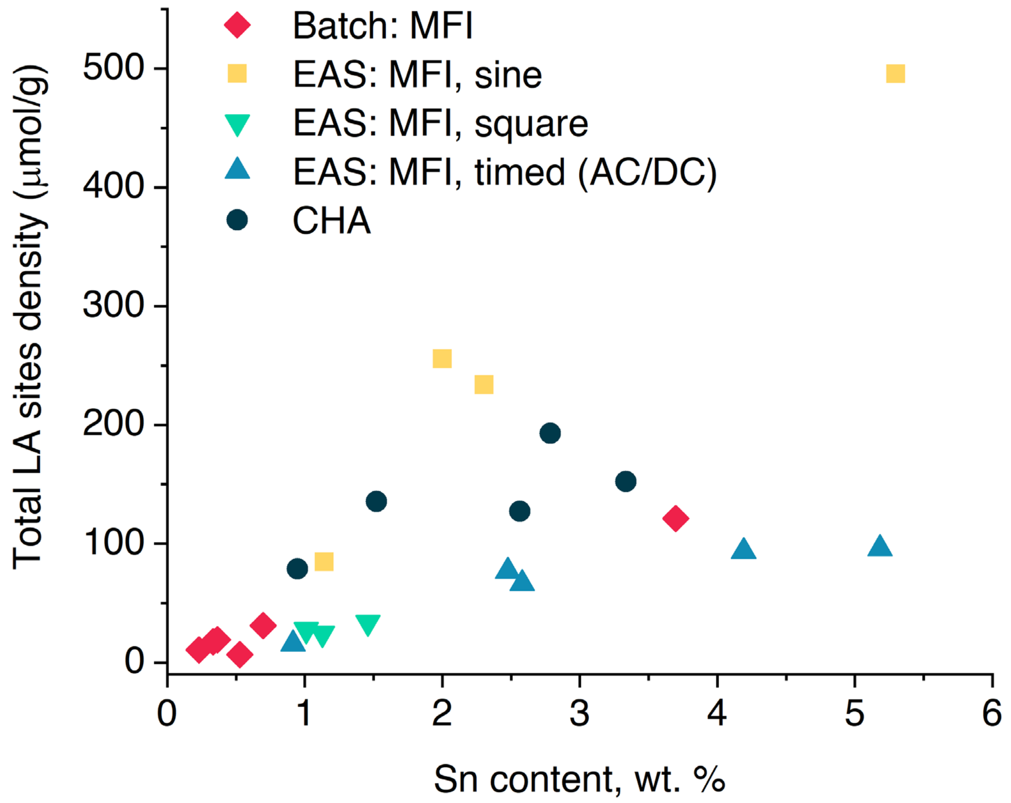

Figure S22. Concentration of Lewis acid sites in Sn-containing zeolites.
The data of adsorbed CD_3CN FT-IR related to the framework tin bands (LAS): 2316 and 2308 cm^{-1} (Table S11). For EAS CHA, blanks and classic batch controls are included.

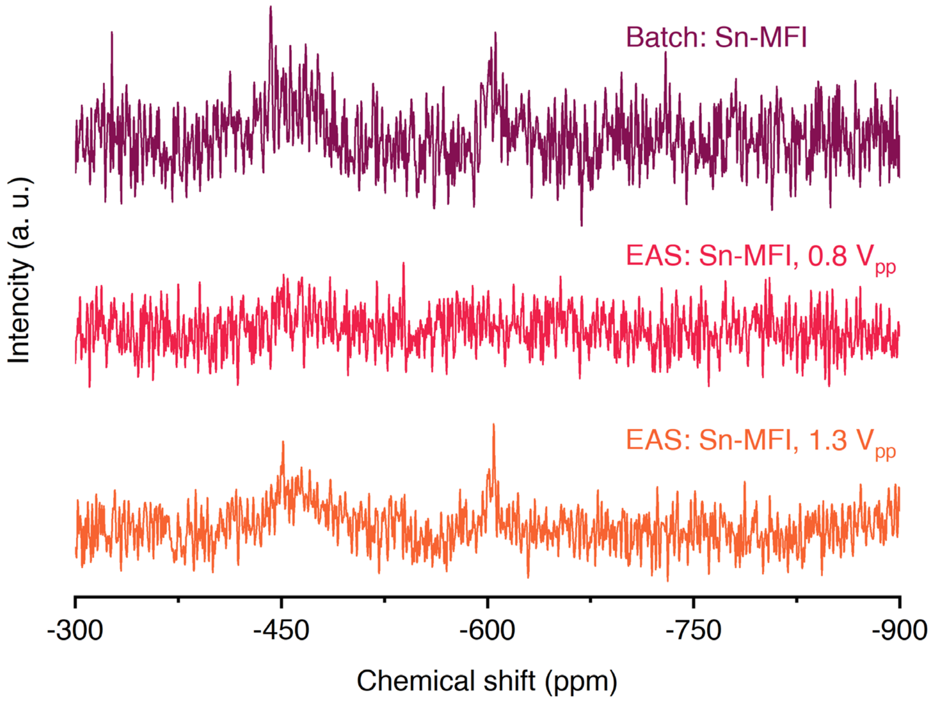

Figure S23. ^{119}Sn Solid-State NMR spectra of dehydrated tin-containing zeolites.

Sn-MFI was made in the classic batch conditions with the starting batch composition Si:37Sn^{-1}:0.5TPAOH:20.0H$_2$O at 160 °C for 10 days to achieve a higher tin content in the final material. The EAS Sn-MFI zeolites were synthesized with applied electricity in the sine waveform at 50 μHz frequency for 72 hours. Immersed area of electrodes equals 13.4 cm^2 for all EAS experiments.

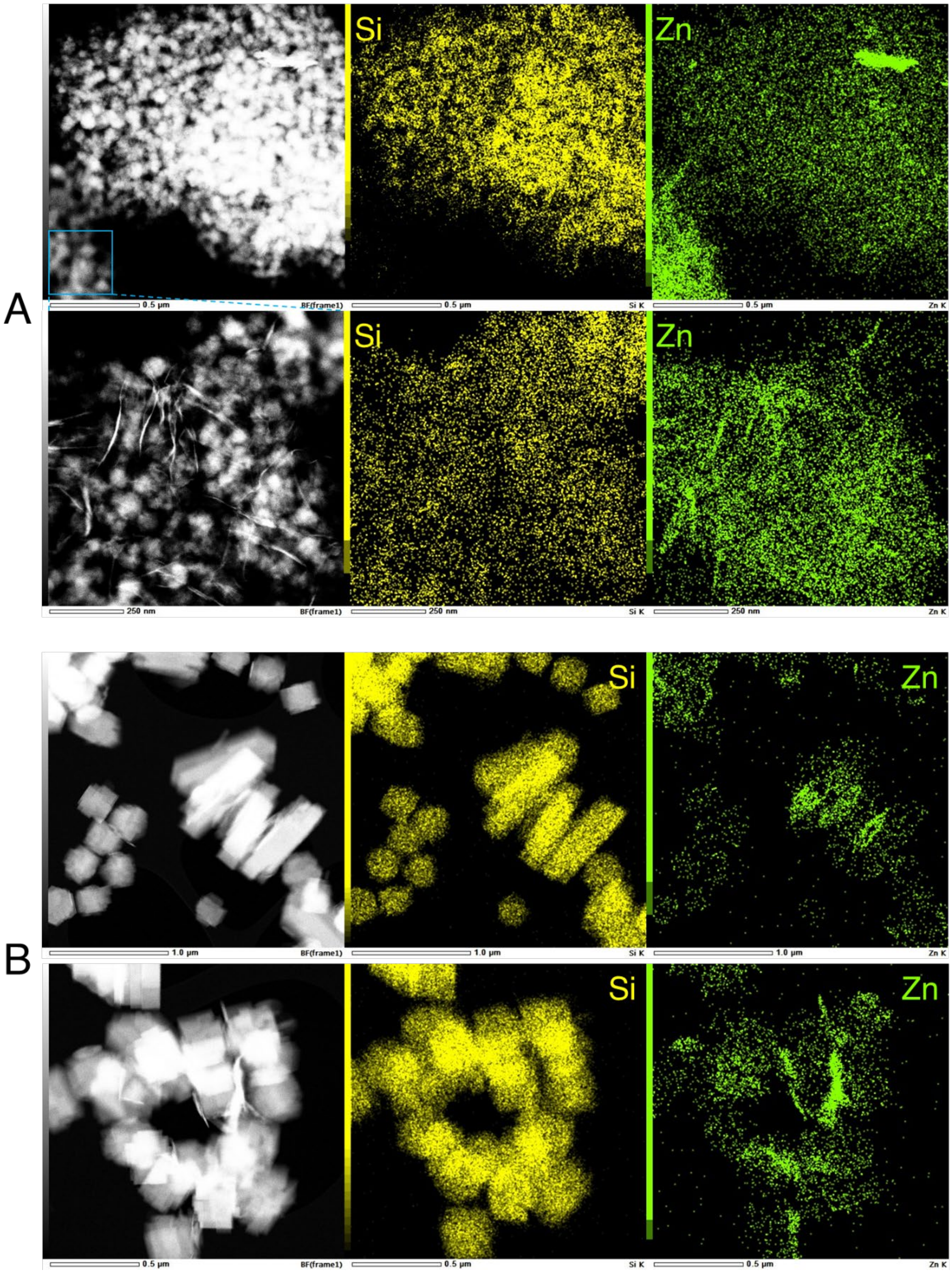

Figure S24. TEM EDX images of Zn-MFI zeolite synthesized in the classic batch conditions. (**A**) The synthesis conducted at 90 °C, and (**B**) at 160 °C.

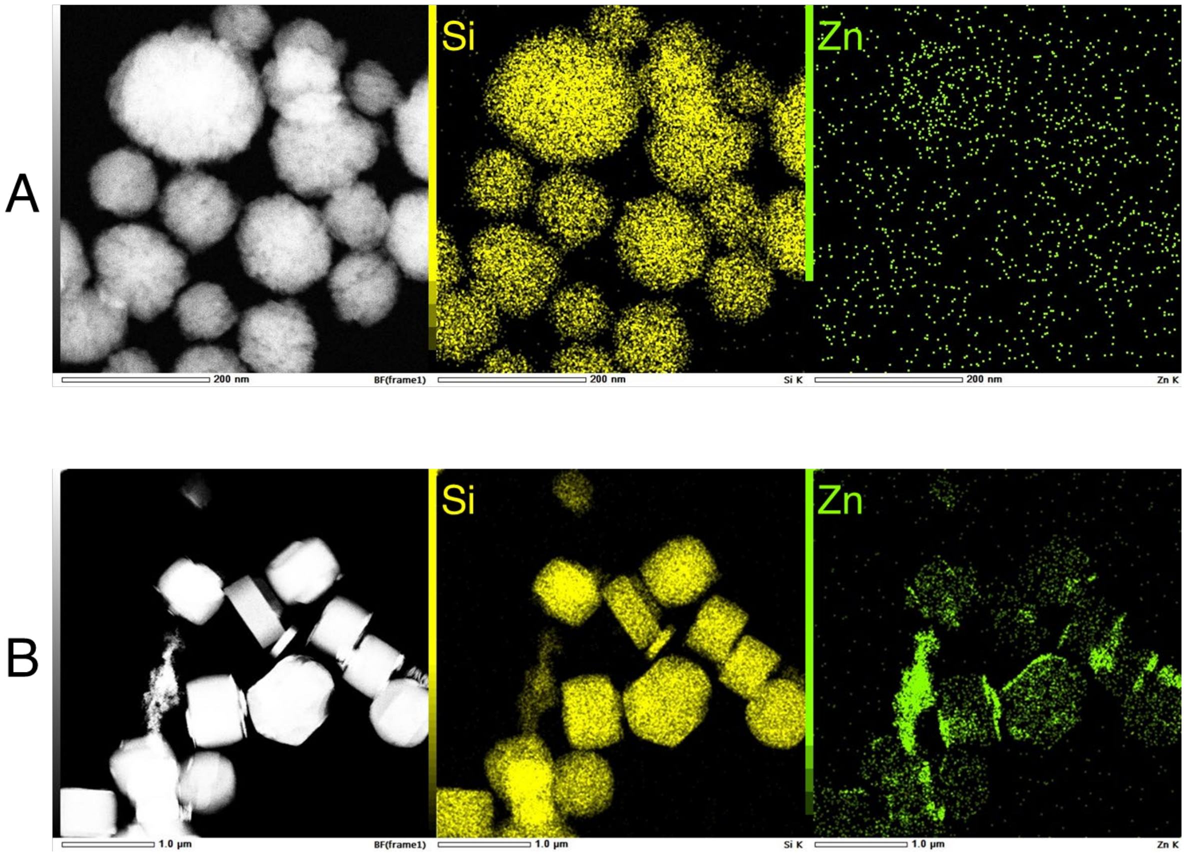

Figure S25. TEM EDX images of Zn-MFI zeolite synthesized in the EAS conditions. (**A**) The synthesis conducted at 90 °C, and (**B**) at 160 °C.

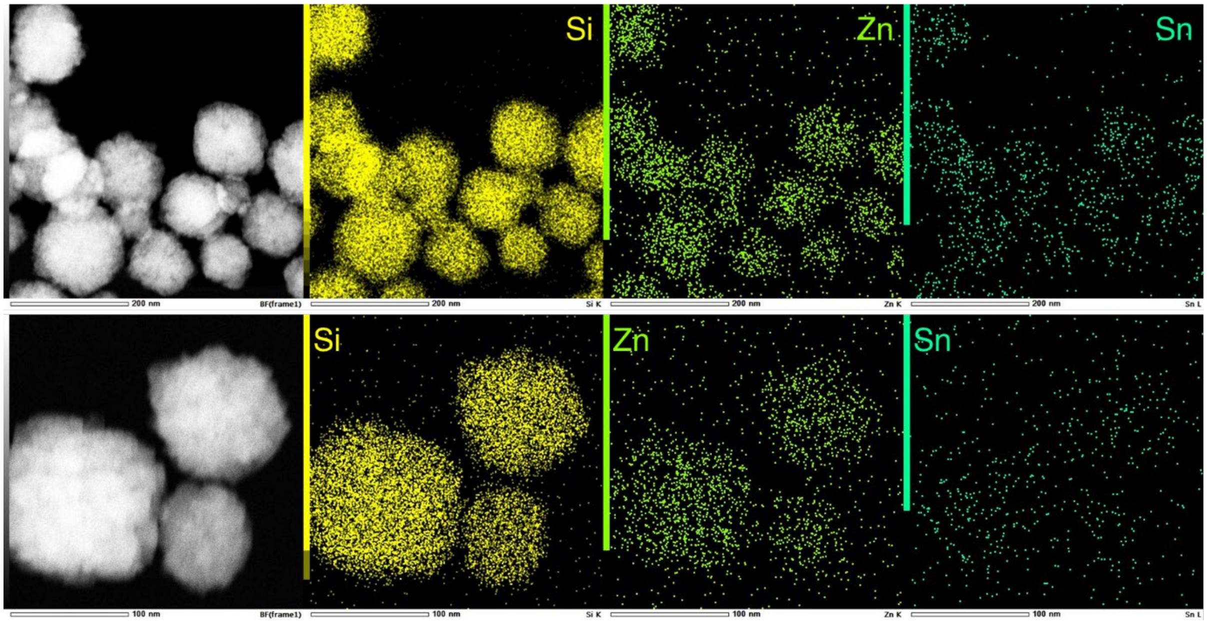

Figure S26. TEM EDX images of Sn,Zn-MFI zeolite synthesized in the EAS conditions.

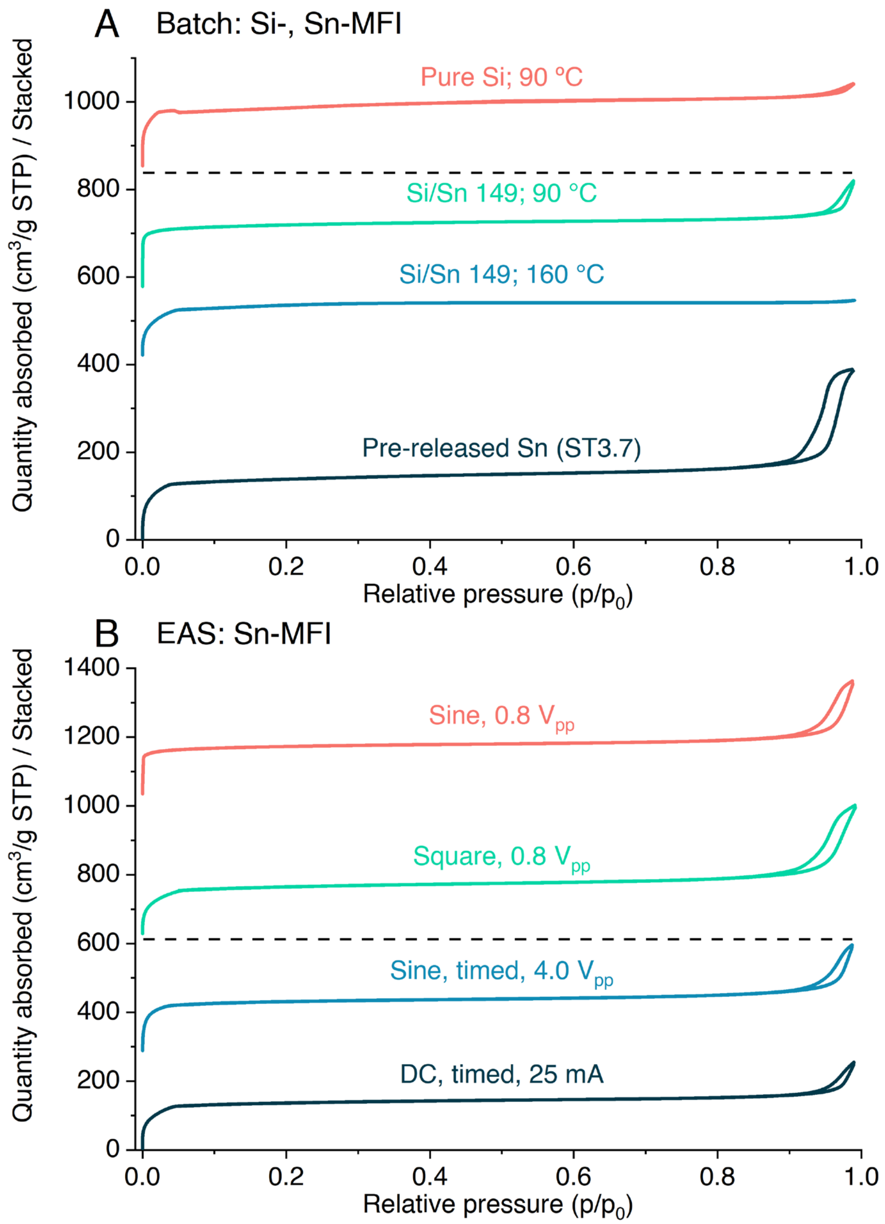

Figure S27. Typical isotherms of low-temperature N_2 adsorption on Si-, Sn-MFI zeolites (stacking on Y-axis begins each time from the maximum value of the last one; dashed line example for first stacking).

(**A**) Si- and Sn-MFI zeolites made in the classic batch conditions, (**B**) Sn-MFI synthesized in the EAS conditions, examples given for the different types of applied electricity.

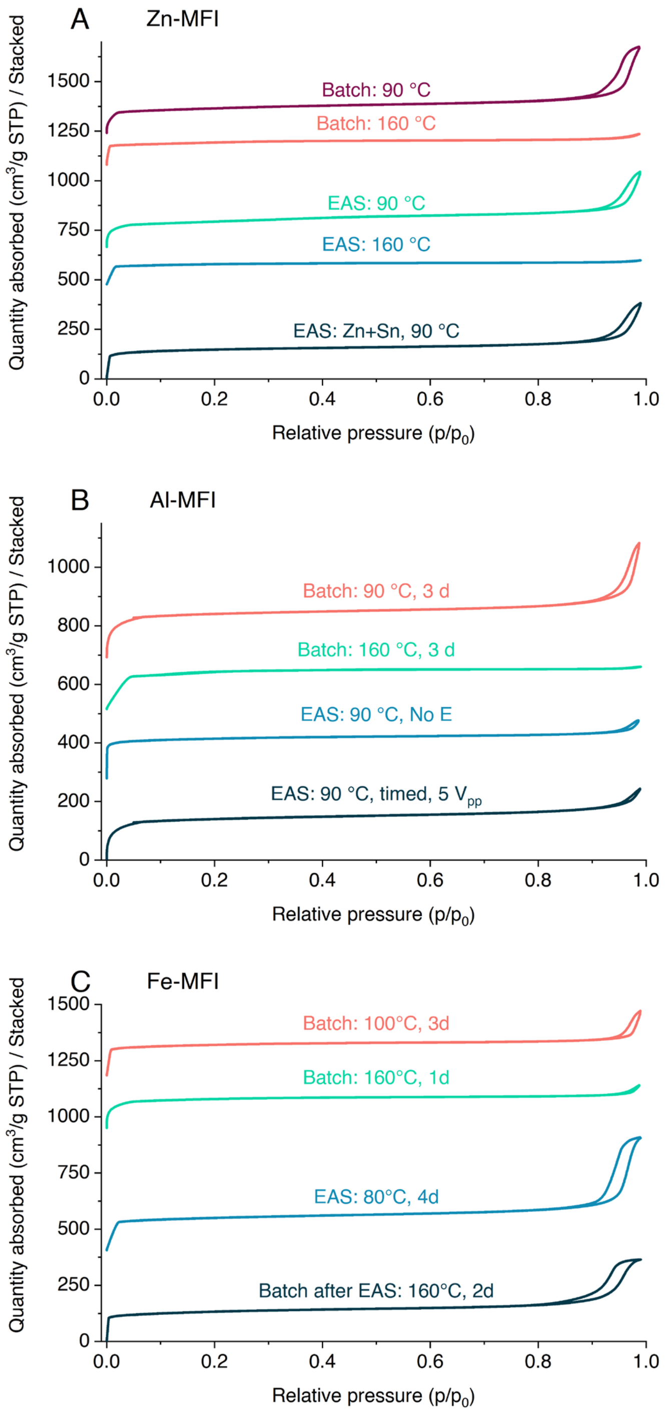

Figure S28. Typical isotherms of low-temperature N_2 adsorption on Zn-, Sn,Zn-, Al- and Fe-MFI zeolites (stacked Y-axis).

(**A**) Zn-containing MFI zeolites synthesized in the classic batch and EAS conditions at various temperatures, (**B**) Al-MFI synthesized in the classic batch and EAS conditions, (**C**) Fe-MFI synthesized in the classic batch and EAS conditions.

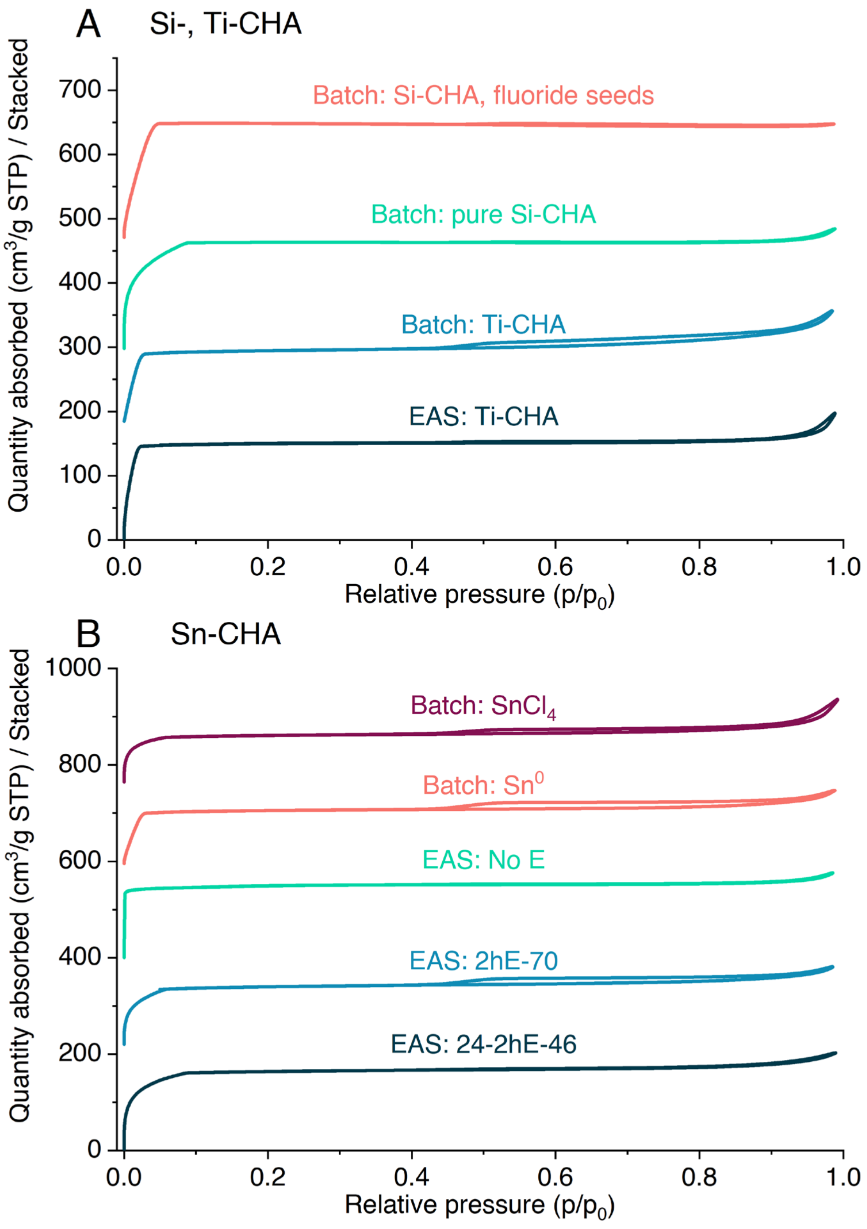

Figure S29. Typical isotherms of low-temperature N_2 adsorption on Si-, Ti-, and Sn-CHA zeolites (stacked Y-axis).

(A) Synthesis of Si- and Ti-CHA zeolites conducted in the classic batch and EAS conditions, (**B**) Sn-CHA zeolites synthesized in the classic batch and EAS conditions.

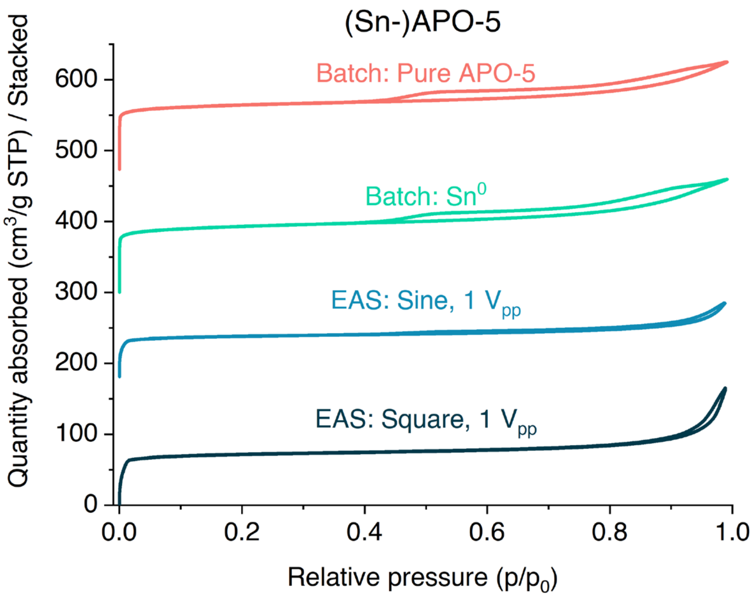

Figure S30. Typical isotherms of low-temperature N_2 adsorption on (Sn-)APO-5 (stacked Y-axis).

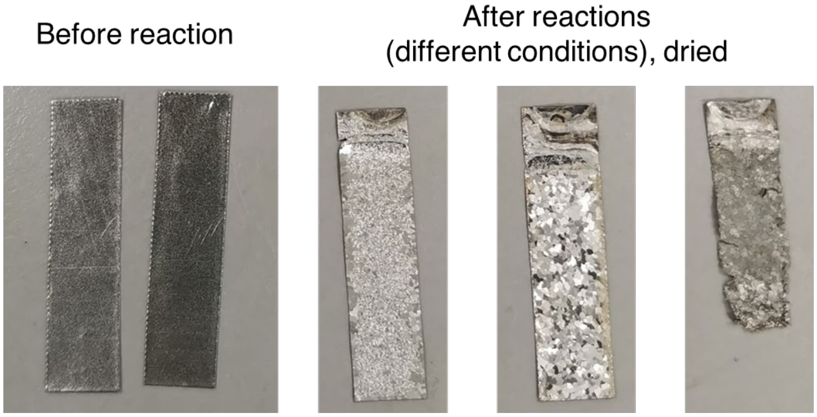

Figure S31. Tin electrodes before and after EAS at different conditions of applied electricity.

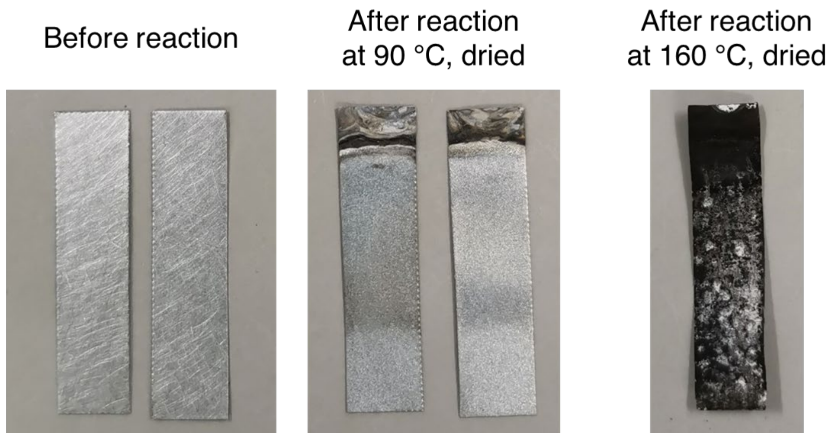

Figure S32. Zinc electrodes before and after EAS at 90 and 160 °C.

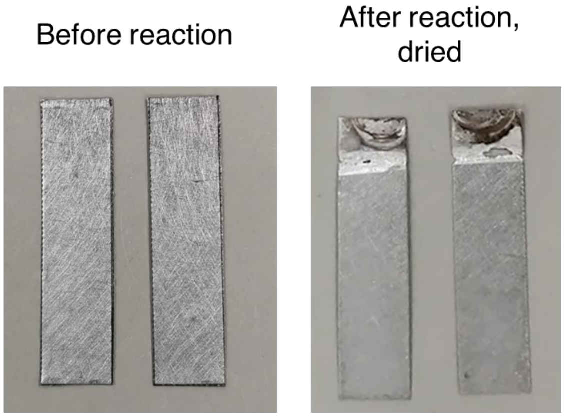

Figure S33. Aluminum electrodes before and after EAS.

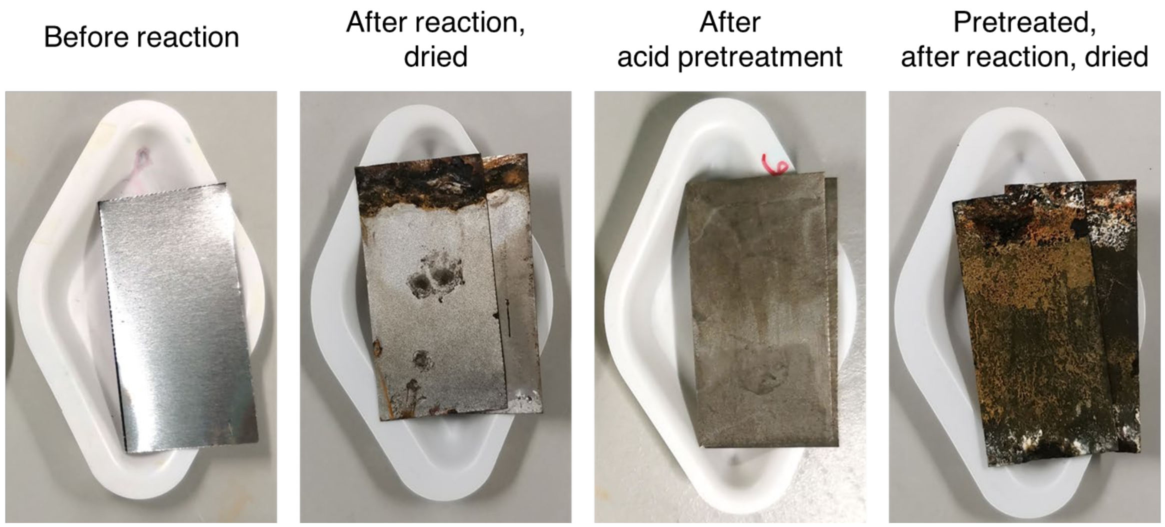

Figure S34. Iron electrodes before, after acid pretreatment, and dried after EAS.

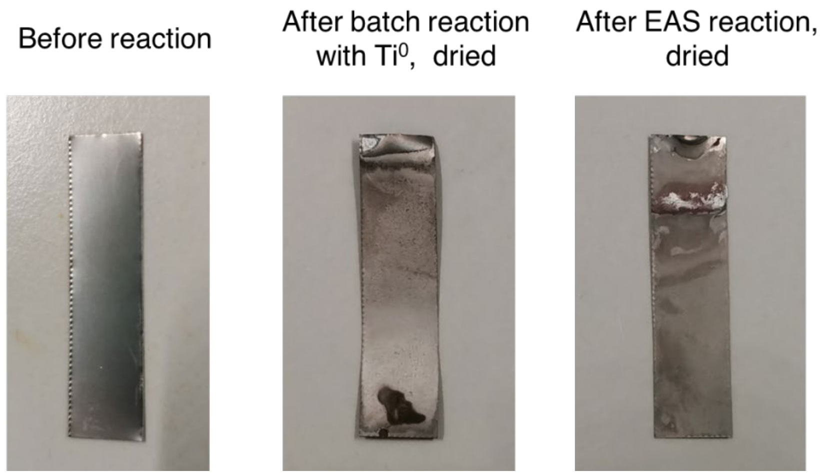

Figure S35. Titanium electrodes before and after EAS.

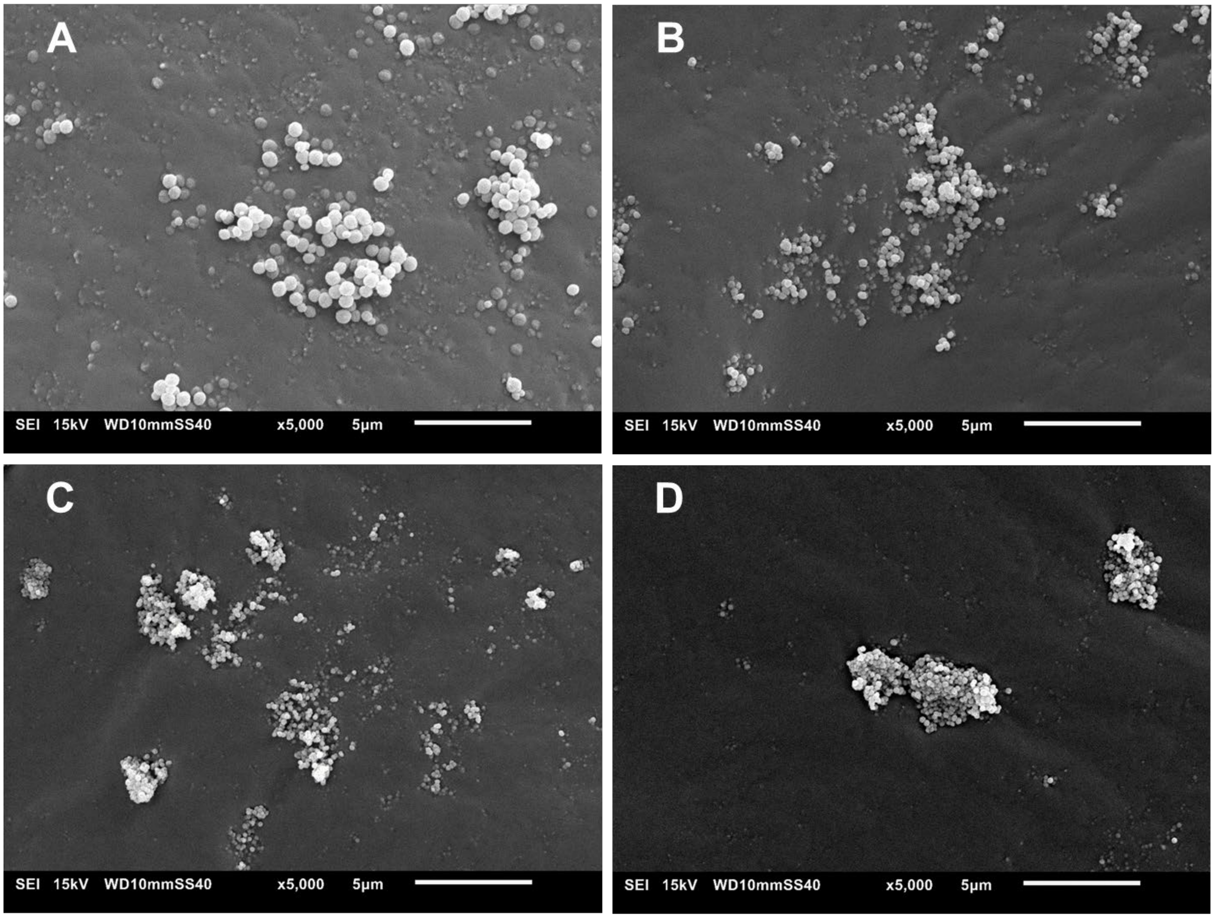

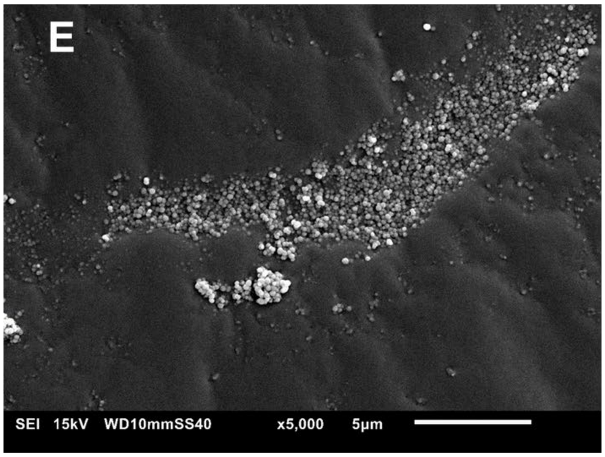

Sample	Crystal size, μm
(**A**) SnO_2/Si-MFI	0.60
(**B**) Batch: Sn-MFI	0.40
(**C**) EAS: Sn-MFI, 0.75V_{pp}	0.25
(**D**) EAS: Sn-MFI, 0.80V_{pp}	0.25
(**E**) EAS: Sn-MFI, 1.30V_{pp}	0.25

Figure S36. SEM images of as-synthesized tin-containing MFI zeolites.

(**A**) SnO_2/Si-MFI, which was made by impregnation of Si-MFI zeolite (OSDA/Si 0.5) with $SnCl_4$ solution and later calcination (as was done for ZnO/Si-MFI – ST2.9); (**B**) Sn-MFI was made in the classic batch conditions with the starting batch composition Si:37Sn^{-1}:0.5TPAOH:20.0H_2O at 160 °C for 10 days to achieve a higher tin content in the final material. The EAS Sn-MFI zeolites were synthesized with applied electricity in the sine waveform at (**C**) 0.75, (**D**) 0.80, and (**E**) 1.3 V_{PP} with 50 μHz frequency for 72 hours; immersed electrode area equals 13.4 cm^2.

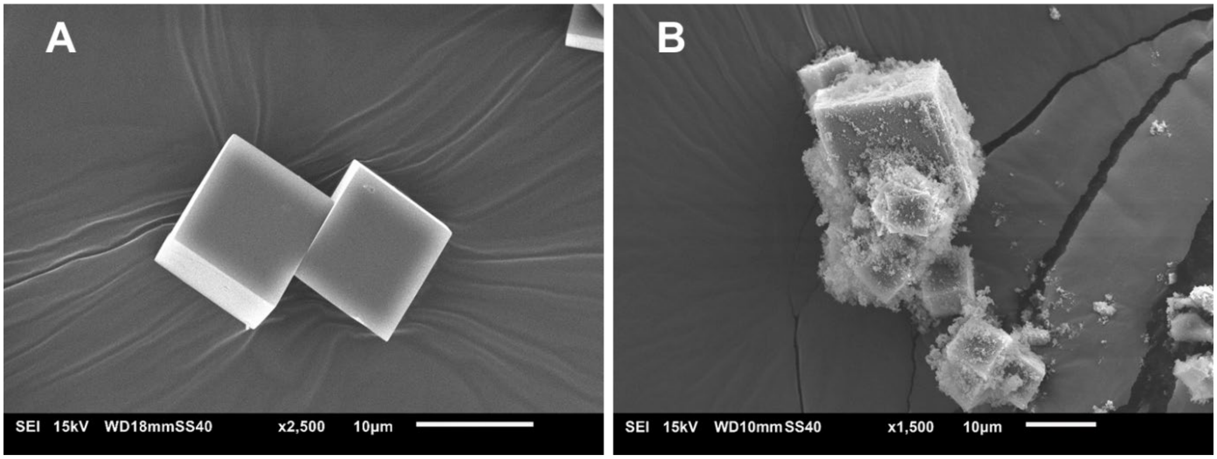

Figure S37. SEM images of as-synthesized Si-CHA zeolites.

(**A**) Si-CHA zeolite synthesized in fluoride media at 160 °C for 48 hours and used as seeds material; (**B**) Si-CHA zeolite synthesized in the classic batch conditions with seeds, from a siliceous gel with the starting batch composition of Si:0.5TMAdamOH:4.0EDA:30.0H_2O at 120 °C for 72 hours. Average crystal size: 10 μm for **A**, and 5 – 15 μm for **B**.

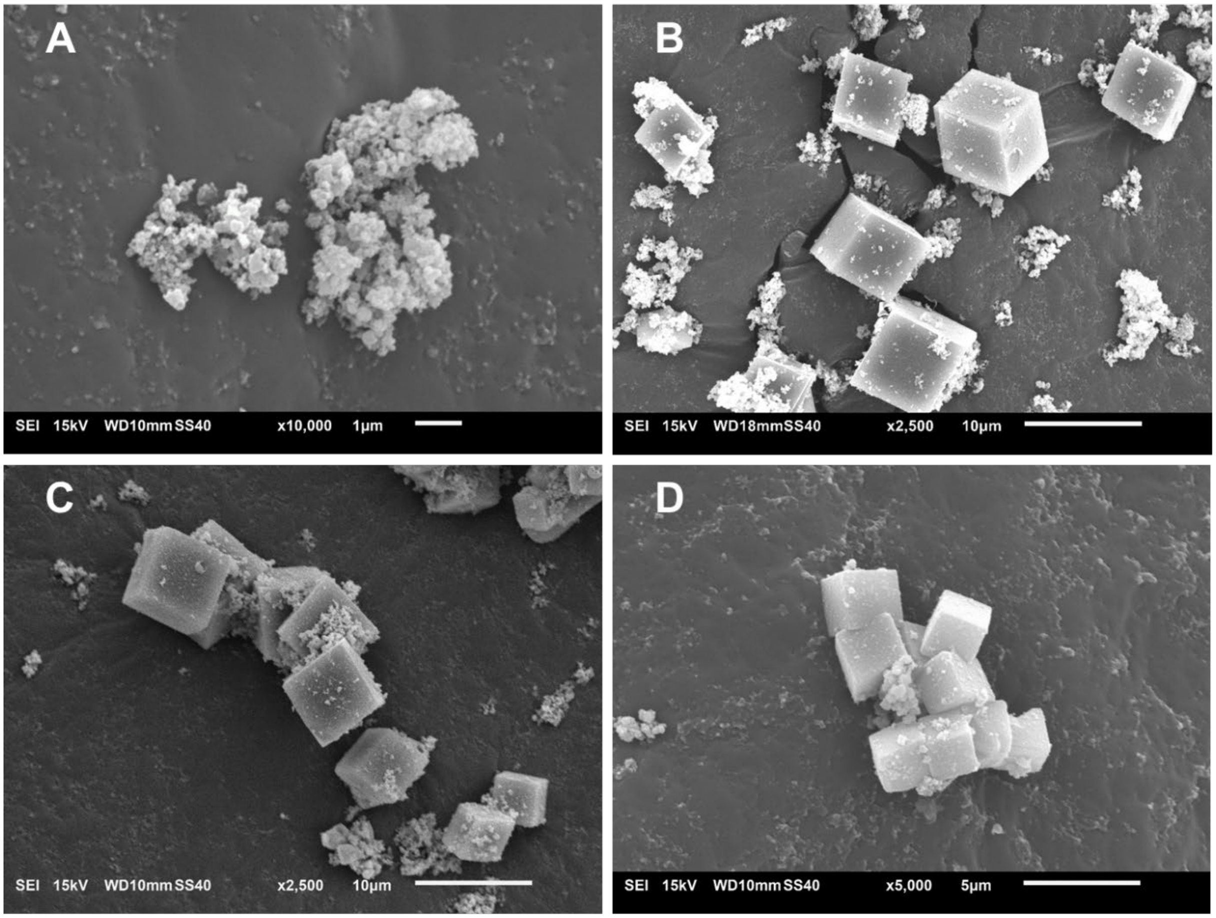

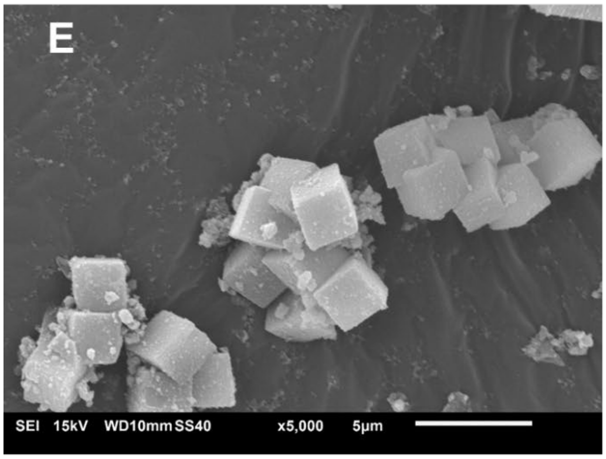

Sample	Crystal size, μm
(**A**) Batch: Sn-CHA, $SnCl_4$	0.1 – 0.3
(**B**) Batch: Sn-CHA, Sn^0	5.0 – 15.0
(**C**) EAS: Sn-CHA, No E	5.0 – 12.0
(**D**) EAS: Sn-CHA, 2hE-70	2.5
(**E**) EAS: Sn-CHA, 24-2hE-46	2.0

Figure S38. SEM images of as-synthesized Sn-CHA zeolites.

(**A**) Sn-CHA synthesized in the classic batch conditions with the starting Si/Sn ratio of 74 using $SnCl_4$ as the tin source; blank samples synthesized in (**B**) batch and (**C**) electro reactor conditions using immersed Sn^0 electrode in the pure siliceous media (ST2.2); **(D+E)** EAS samples with applied electricity in the starting point of (**D**) the first or (**E**) the second 24 hours of a 72 hours experiment with the following characteristics: 2 V_{PP}; 25 μHz; 2 hours; square wave; immersed area of electrodes equals 13.4 cm^2.

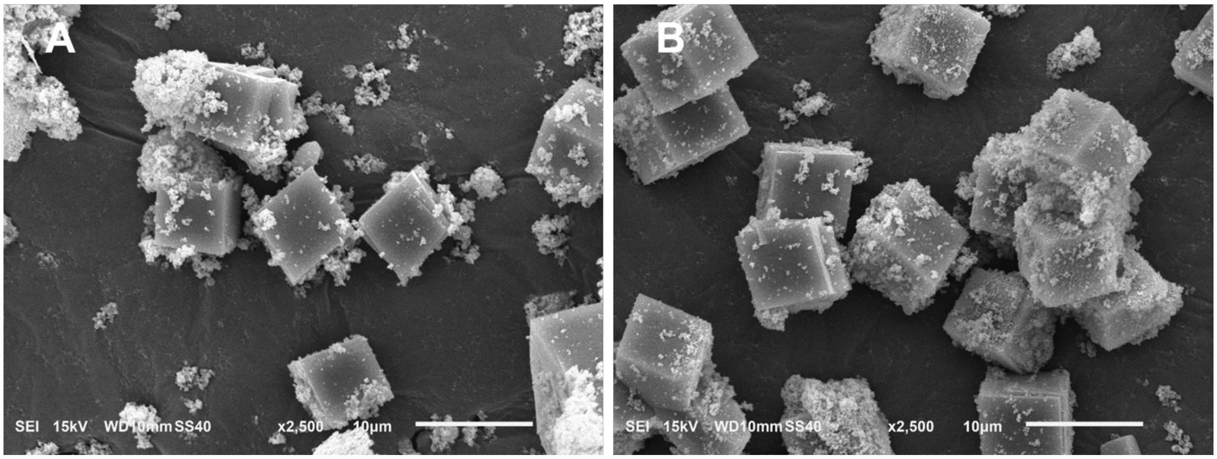

Figure S39. SEM images of as-synthesized Ti-CHA zeolites.

(**A**) Ti-CHA zeolite sample synthesized in the classic batch conditions with immersed Ti^0 electrode; (**B**) Ti-CHA zeolite synthesized via the EAS route with applied electricity at the starting point of the second 24 hours of a 72 hours experiment with the following characteristics: 2 V_{PP}; 25 µHz; 2 hours; square wave; immersed area of electrodes equals 13.4 cm^2. Average crystal size: 5 – 15 µm for **A**, and 5 – 10 µm for **B**.

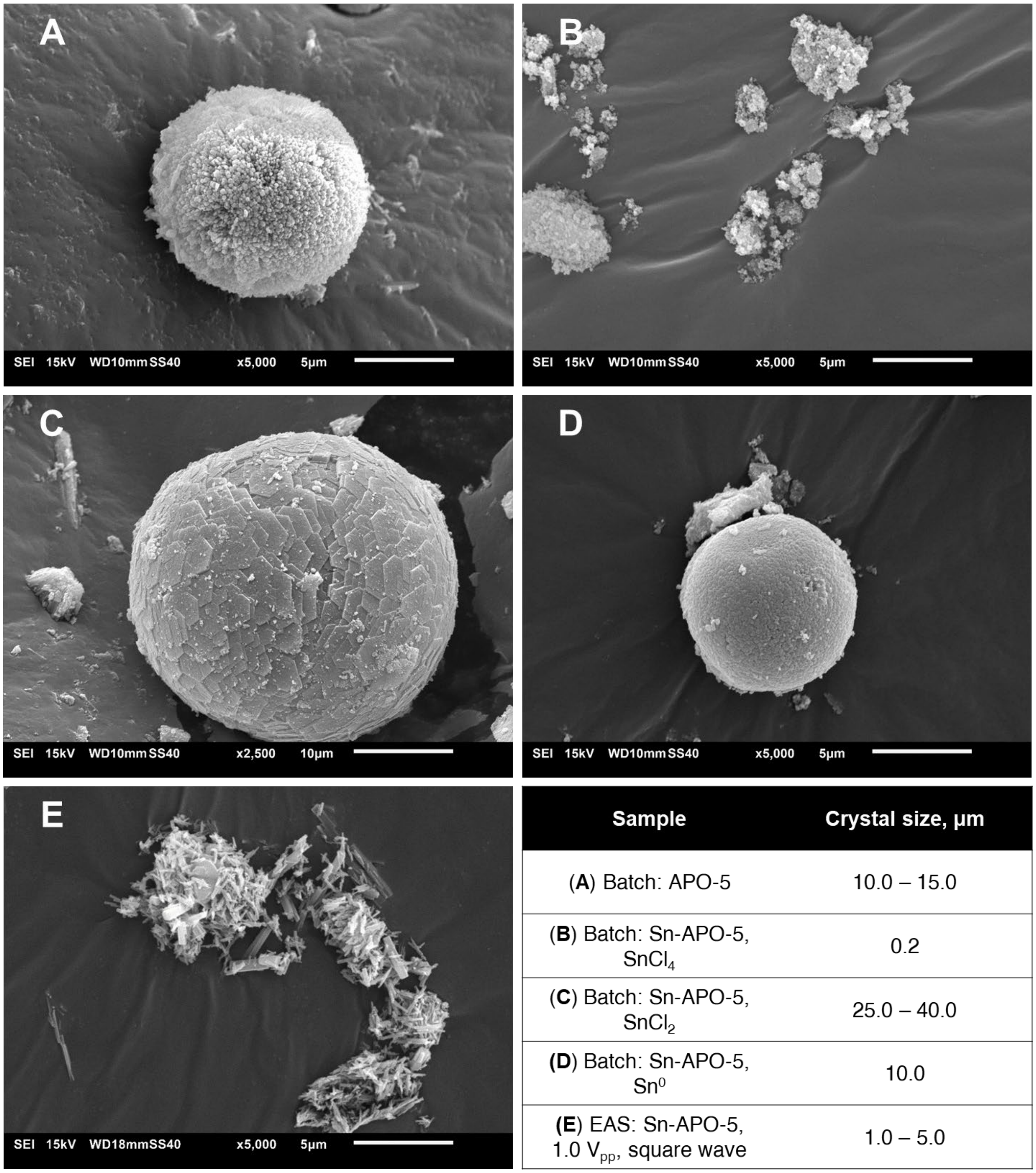

Sample	Crystal size, µm
(**A**) Batch: APO-5	10.0 – 15.0
(**B**) Batch: Sn-APO-5, $SnCl_4$	0.2
(**C**) Batch: Sn-APO-5, $SnCl_2$	25.0 – 40.0
(**D**) Batch: Sn-APO-5, Sn^0	10.0
**(E)** EAS: Sn-APO-5, 1.0 V_{pp}, square wave	1.0 – 5.0

Figure S40. SEM images of as-synthesized (Sn-)APO-5.

(**A**) Pure aluminophosphate APO-5; Sn-APO-5 were synthesized in the classic batch conditions with the addition of (**B**) $SnCl_4$ and (**C**) $SnCl_2$ salts as the metal precursors; (**D**) blank Sn-APO-5 sample was made in the batch conditions with immersed Sn^0 electrode. (**E**) The EAS Sn-APO-5 was synthesized with applied electricity in the square waveform at 1.0 V_{PP} with 50 µHz frequency for 24 hours; immersed electrode area equals 13.4 cm^2.

Tables

Table S1. Synthesis conditions and characteristics of batch synthesized Sn-MFI zeolites.
Starting gel composition: Si:(XSn):0.5TPAOH:20.0H_2O.

Synthesis temperature, °C	Synthesis duration, h	Metal source	Theoretical Si/Sn ratio	Final material Si/Sn ratio [a]	Surface area (S_{BET}), m^2 g^{-1}	Microporous volume, cm^3 g^{-1}
			Classic batch synthesis			
90	72	-	∞		517	0.14
90	72	$SnCl_4$	446	1787 ± 124	526	0.17
90	72	$SnCl_4$	149	852 ± 32	561	0.15
90	72	$SnCl_4$	74	281 ± 9	557	0.16
160	72	-	∞		443	0.13
160	72	$SnCl_4$	149	275 ± 6	441	0.12
160	72	$SnCl_4$	37	87	476	0.12
160	240	$SnCl_4$	37	51	455	0.12
90	72	$SnCl_4$	125 [b]	538	499	0.15
90	72	Sn^0	125 [b]	372	532	0.16
90	72	Sn^0	142 [b]	590	508	0.13

[a] The error is based on ICP-AES analysis performed twice

[b] Samples made with speciation theory of released tin (ST2.7)

Table S2. Synthesis conditions, AC-mode EAS parameters and characteristics of electro-synthesized Sn-MFI zeolites.

Synthesis conditions: 90 °C, 72 hours, starting gel composition: Si:0.5TPAOH:20.0H_2O. Metal tin (Sn^0) was used as an electrode material for all EAS experiments, frequency was set to 50 μHz.

Electrode area, cm^2	Voltage, V_{PP}	Timing of applied electricity, h	Waveform	R_r [a]	Final material Si/Sn ratio [b]	Surface area (S_{BET}), m^2 g^{-1}	Microporous volume, cm^3 g^{-1}
			Electro-assisted synthesis				
30.8		No E			877	542	0.16
13.4	0.60	72	Sine	0.7	927	549	0.17
13.4	0.65	72	Sine	1.3	206	535	0.14
13.4	0.70	72	Sine	1.6	139 ± 6	526	0.14
13.4	0.75	72	Sine	1.5	173 ± 6	523	0.13
6.7	0.80	72	Sine	3.1	171	524	0.16
13.4	0.80	72	Sine	2.4	96	539	0.13
22.6	0.80	72	Sine	1.6	83 ± 3	536	0.12
13.4	0.85	72	Sine	1.4	130	543	0.18
13.4	0.90	72	Sine	1.7	134	541	0.17
13.4	0.95	72	Sine	1.8	148	549	0.14
13.4	1.00	72	Sine	3.1	54	638	0.12
30.8	1.00	72	Sine	1.7	42	589	0.12
13.4	1.10	72	Sine	2.4	71 ± 2	550	0.14
13.4	1.20	72	Sine	3.0	74	541	0.16
13.4	1.30	72	Sine	4.3	32	609	0.13
13.4	1.50	72	Sine	5.9	35	599	0.12

Electrode area, cm^2	Voltage, V_{PP}	Timing of applied electricity, h	Waveform	R_r [a]	Final material Si/Sn ratio [b]	Surface area (S_{BET}), m^2 g^{-1}	Microporous volume, cm^3 g^{-1}
13.4	0.70	72	Square	2.3	173	542	0.16
13.4	0.75	72	Square	2.5	193	549	0.16
13.4	0.80	72	Square	3.5	133	522	0.16
13.4	2.00	16-8E-48	Sine	16.2	77	538	0.14
13.4	4.00	16-8E-48	Sine	34.9	45	554	0.17

[a] Release rate (R_r), (mol cm^{-2} s^{-1})×1e-10

[b] The error is based on ICP-AES analysis performed twice

Table S3. DC-mode EAS parameters and characteristics of Sn-MFI zeolite.

Synthesis conditions: 90 °C, 72 hours, starting gel composition: Si:0.5TPAOH:20.0H_2O. Electrode material – Sn^0, immersed electrode surface area 13.4 cm^2, electricity was applied in the timed regime (ST2.8), in the direct (constant) current mode for 8 hours: 16-8hE-48.

Current, mA	Release rate (R_r), (mol cm^{-2} s^{-1})×1e-10	Final material Si/Sn ratio	Surface area (S_{BET}), m^2 g^{-1}	Microporous volume, cm^3 g^{-1}	Faradaic efficiency, %
2	22.5	60	554	0.17	930 [a]
4	13.6	214	550	0.16	190 [a]
8	12.3	325	547	0.14	80
16	27.5	74	528	0.16	85
25	29.7	72	528	0.14	53
30	37.3	38	549	0.14	66
35	34.1	36	520	0.16	57

[a] These unrealistic values were not accounted for the average Faradaic efficiency calculation. The error can possibly come from the low preciseness of the DC power supply on low currents (machine threshold), and side reactions caused by relatively high voltage (needed to maintain a set current level: Figure S2) compared to general electrochemical values

Table S4. Synthesis conditions, AC-mode EAS parameters and characteristics of Zn-MFI zeolite.

Synthesis conditions: 72 hours, starting gel composition: Si:(33Zn):0.5TPAOH:20.0H_2O. Sine wave with 50 μHz frequency was used for all EAS experiments.

°C [a]	Metal source	Starting Si/Zn ratio	Electrode area, cm^2	Voltage, V_{PP}	Time of applied electricity, h	R_r [b]	Final material Si/Zn ratio	Surface area (S_{BET}), $m^2\ g^{-1}$	Microporous volume, $cm^3\ g^{-1}$
				Classic batch synthesis					
90	$Zn(OAc)_2$	33					25	465	0.10
160	$Zn(OAc)_2$	33					23	420	0.12
90	Zn^0	∞	13.4		No E		296	561	0.15
				Electro-assisted synthesis					
90	Zn^0	∞	13.4	0.80	72	7.4	45	480	0.11
160	Zn^0	∞	13.4	0.80	72	7.4	21	401	0.11
90	Zn^0	∞	13.4	5.00	16-8E-48	117.1	44	541	0.12
90	Zn^0	∞	6.7	1.00	36	17.9	37	564	0.11
	Sn^0	∞	6.7		36	3.1	107		

[a] Synthesis temperature

[b] Release rate (R_r), (mol cm^{-2} s^{-1})×1e-10

Table S5. Ion exchange performance of Zn-containing MFI zeolites.

Reactor type and EAS specifics		Temperature, °C	Si/Zn (Si/Sn) before ion exchange	Si/Zn (Si/Sn [a]) after ion exchange [b]	Loss of Zn atoms, %	K/2Zn (K/2Sn [a])	Ni/Zn (Ni/Sn [c])
Impregnated		160	19	20 ± 1	5	0.06	0.01
			38	41 ± 1	10	0.06	0.01
			88	97 ± 1	10	0.09	0.02
			218	222 ± 3	1	0.12	0.02
Batch		90	25	42 ± 5	39	0.19	0.73
		160	23	27 ± 1	14	0.07	0.03
EAS	Classic	90	46 ± 1 [d]	74 ± 12	37	0.26	0.38
	Timed	90	44	63 ± 9	31	0.18	0.29
	Zn+Sn	90	37 (107)	55 ± 9 (96 ± 13)	32	0.41	0.77
	Classic	160	21	28 ± 2	24	0.23	0.18
	Sn	90	(94)	(81 ± 1)		(0.14)	(0.10)

[a] Measured by the HF ICP-AES method, which is not reliable for Sn concentration. Therefore, through coefficients between Si/Sn values measured by HF and oven methods, an approximation was applied for Sn

[b] The error is based on ICP-AES analysis performed separately on Zn-MFI samples after Ni^{2+} and K^{+} ion-exchange

[c] The error is based on ICP-AES analysis performed for 2 separate and identical Zn-MFI made by EAS

Table S6. Synthesis conditions, AC-mode EAS parameters and characteristics of Al-MFI zeolite.

Synthesis conditions: 72 hours, starting gel composition: Si:(114Al):0.5TPAOH:20.0H_2O. Sine wave with 50 µHz frequency was used for the EAS experiment.

°C [a]	Metal source	Theoretical Si/Zn ratio	Electrode area, cm^2	Voltage, V_{PP}	Time of applied electricity, h	R_r [b]	Final material Si/Al ratio	Surface area (S_{BET}), m^2 g^{-1}	Microporous volume, cm^3 g^{-1}
				Classic batch synthesis					
90	$Al(NO_3)_3$	114					76	569	0.17
160	$Al(NO_3)_3$	114					108	504	0.12
				Electro-assisted synthesis blank control in electro reactor					
90	Al^0	∞	13.4		No E		471	532	0.14
90	Al^0	∞	13.4	5.00	24-24E-24	5.1	143	534	0.16

[a] Synthesis temperature

[b] Release rate (R_r), (mol cm^{-2} s^{-1})×1e-10

Table S7. Synthesis conditions, AC-mode EAS parameters and characteristics of Fe-MFI zeolite.

Starting gel composition: Si:(XFe):0.5TPAOH:20.0H_2O. Sine wave with 50 µHz frequency was used for the EAS experiment, immersed electrode area was set to 13.4 cm^2.

°C [a]	Synthesis duration, h	Metal source	Theoretical Si/Zn ratio	Voltage, V_{PP}	Time of applied electricity, h	R_r [b]	Final material Si/Fe ratio	Surface area (S_{BET}), $m^2\ g^{-1}$	Microporous volume, $cm^3\ g^{-1}$
Classic batch synthesis									
100	72	$Fe(NO_3)_3$	334				210	528	0.13
160	24	$Fe(NO_3)_3$	334				200	488	0.14
160	72	$Fe(NO_3)_3$	60				36	477	0.11
Electro-assisted synthesis and blank control in electro reactor									
80	96	Fe^0	∞	10.00	96	0.3	75	554	0.14
160	48	Fe^X					162	497	0.12

[a] Synthesis temperature

[b] Release rate (R_r), (mol $cm^{-2}\ s^{-1}$)×1e-10

Fe^x = pre-released iron of unknown oxidation state

Table S8. Synthesis conditions, AC-mode EAS parameters and characteristics of Sn-, Ti-CHA zeolite.

Starting gel composition: Si:(74Sn):0.5TMAdamOH:4.0EDA:6.0F^-:20.0H_2O. Square wave with 25 μHz frequency was used for EAS experiments.

°C [a]	Synthesis duration, h	Metal source	Theoretical Si/Zn ratio	Electrode area, cm^2	Voltage, V_{PP}	Time of applied electricity, h	R_r [b]	Final material Si/Me ratio	Surface area (S_{BET}), $m^2\ g^{-1}$	Microporous volume, $cm^3\ g^{-1}$
					Classic batch synthesis					
160 [c]	48	-							767	0.28
120	72	$SnCl_4$	74					206	381	0.13
120	72	-							660	0.26
120	72	Sn^0	∞	13.4		No E		75	472	0.15
120	72	Ti^0	∞	13.4		No E		21	457	0.15
					Electro-assisted synthesis					
120	72	Sn^0	∞	13.4		No E		127	612	0.20
120	72	Sn^0	∞	13.4	2.00	2E-70	83.4	57	485	0.16
120	72	Sn^0	∞	13.4	2.00	24-2E-46	98.5	68	642	0.24
120	72	Ti^0	∞	13.4	2.00	24-2E-46	94.8	62	639	0.21

[a] Synthesis temperature

[b] Release rate (R_r), (mol $cm^{-2}\ s^{-1}$)×1e-10

[c] Fluoride synthesis of Si-CHA (used as seeds)

Table S9. Synthesis conditions, AC-mode EAS parameters and characteristics of Sn-APO-5 aluminophosphate.

Synthesis conditions: 160 °C, 24 hours, starting gel composition: Al:(1-X)P:(XSn):0.8OSDA:50.0H_2O. For EAS experiments, voltage difference was applied during all synthesis time (24 hours) with 50 μHz frequency.

Metal source	Theoretical Al/Sn ratio	Electrode area, cm^2	Voltage, V_{PP}	Waveform	R_r [a]	Final material Al/Sn ratio [b]	Surface area (S_{BET}), m^2 g^{-1}	Microporous volume, cm^3 g^{-1}
Classic batch synthesis								
-							367	0.10
$SnCl_4$	34					36 ± 1	290	0.05
$SnCl_2$	30					27 ± 2	337	0.09
Sn^0	∞	13.4		No E		171 ± 14	371	0.10
Electro-assisted synthesis								
Sn^0	∞	13.4	1.00	Sine	8.6	37 ± 1	223	0.06
Sn^0	∞	13.4	1.00	Square	7.2	81 ± 1	279	0.07
Sn^0	∞	13.4	1.50	Sine	6.8	63 ± 1	270	0.08
Sn^0	∞	13.4	2.00	Sine	9.6	59 ± 1	100	0.01

[a] Release rate (R_r), (mol cm^{-2} s^{-1})×1e-10

[b] The error is based on ICP-AES analysis performed twice

Table S10. Conditions of Si-MFI support synthesis, and characterization data of impregnated samples. The description of the impregnation procedure is given in ST2.9.

Synthesis conditions						
Temperature, °C	OSDA/Si ratio	Metal source	Theoretical Si/Zn ratio	Final material Si/Me ratio	Surface area (S_{BET}), $m^2\ g^{-1}$	Microporous volume, $cm^3\ g^{-1}$
90	0.5	-	-		517	0.14
160	0.2	-	-		476	0.13
160	0.2	$Zn(OAc)_2$	15	19	418	0.12
160	0.2		30	38	450	0.14
160	0.2		90	88	468	0.14
160	0.2		180	218	458	0.14
90	0.5	$SnCl_4$	180	198	528	0.17

Table S11. FT-IR adsorption of deuterated acetonitrile on Sn-containing zeolites.

Zeolite structure	Reactor type	Sn loading		Amount of acid sites (per band), µm g^{-1}				
		Final material Si/Sn ratio	wt. %	2316	2308	Total LAS	2287	2275
MFI	Batch	198 [a]	1.0	0.0	0.0	0.0	21.0	331.5
		852	0.2	3.3	7.3	10.6	7.1	138.8
		281	0.7	7.8	23.3	31.1	35.0	489.5
		538 [b]	0.4	4.4	14.7	19.1	36.6	517.4
		372 [b]	0.5	0.5	6.4	6.8	8.2	171.9
		590 [b]	0.3	3.8	13.7	17.5	44.7	285.3
		51 [c]	3.7	25.5	95.7	121.1	60.9	318.4
	EAS: sine	171	1.1	10.9	73.8	84.6	6.8	219.2
		96	2.0	81.2	174.3	255.5	16.7	542.9
		83	2.3	74.1	159.5	233.5	14.5	332.5
		35	5.3	78.4	417.0	495.4	56.7	684.2
	EAS: Square	173	1.1	9.6	14.8	24.4	37.7	433.5
		193	1.0	9.6	17.9	27.5	42.2	448.3
		133	1.5	8.9	24.8	33.7	39.3	490.8
	EAS: Timed	77	2.5	5.0	71.8	76.8	26.4	329.2
		45	4.2	40.1	53.4	93.4	29.1	129.8
	EAS: DC	214	0.9	1.9	13.8	15.7	32.8	417.3
		74	2.6	28.0	38.6	66.6	55.8	519.9
		36	5.2	20.7	75.2	95.9	31.7	427.4

Zeolite structure	Reactor type	Sn loading		Amount of acid sites (per band), μm g^{-1}				
		Final material Si/Sn ratio	wt. %	2316	2308	Total LAS	2287	2275
CHA	Batch	206	0.9	24.0	54.6	78.7	47.9	448.1
		75	2.6	43.4	84.0	127.3	72.8	394.3
	EAS	127	1.5	44.8	90.7	135.4	105.2	389.4
		57	3.3	14.4	138.0	152.4	56.3	528.2
		68	2.8	22.5	170.5	193.0	65.8	512.9

[a] The sample was made by wetness impregnation (Table S10)

[b] Samples made with speciation theory of pre-released tin (ST2.7)

[c] Sn-MFI sample made in the batch conditions at 160 °C for 10 days

Table S12. Used generators of an electric signal for synthesis of various materials.

Generator	Waveform	Synthesized material
MFG	Sine	Fe-MFI
		Zn-MFI
	Square	Fe-MFI
SRS	Sine	Al-MFI
		Sn-MFI
		Sn,Zn-MFI
		Sn-APO-5
	Square	Sn-MFI
		Sn-CHA
		Ti-CHA
DC Power supply	DC	Sn-MFI

References

(1) Koike, N.; Iyoki, K.; Keoh, S. H.; Chaikittisilp, W.; Okubo, T. Synthesis of New Microporous Zincosilicates with CHA Zeolite Topology as Efficient Platforms for Ion-Exchange of Divalent Cations. *Chemistry - A European Journal* **2018**, *24* (4), 808–812. https://doi.org/10.1002/chem.201705277.

(2) Beverskog, B. Revised Pourbaix Diagrams for Iron At 25-300 °C. *Corros Sci* **1996**, *38* (12), 2121–2135.

(3) Sánchez-Sánchez, M.; van Grieken, R.; Serrano, D. P.; Melero, J. A. On the Sn(II) and Sn(IV) Incorporation into the AFI-Structured AlPO 4-Based Framework: The First Significantly Acidic SnAPO-5. *J Mater Chem* **2009**, *19* (37), 6833–6841. https://doi.org/10.1039/b905057d.
